# Immunoinformatics and reverse vaccinology design of self-amplifying RNA vaccine against marburg virus with structural dynamics and immune simulation analysis

**DOI:** 10.1371/journal.pone.0361136

**Published:** 2026-10-23

**Authors:** Md. Aftabur Rahman, Lamyea Kabir, Nadia Ahmed, Atish Barua, Mohammad Hasan, Ahmad Abdullah Mahdeen

**Affiliations:** 1 Department of Biotechnology and Genetic Engineering, Jahangirnagar University, Dhaka, Bangladesh; 2 Department of Biochemistry and Molecular Biology, Jahangirnagar University, Dhaka, Bangladesh; 3 Department of Biochemistry and Biotechnology, School of Health and Life Sciences, North South University, Dhaka, Bangladesh; 4 Department of Zoology, Government City College, Chattogram, Bangladesh; 5 Department of Biochemistry and Microbiology, North South University, Dhaka, Bangladesh; 6 Department of Microbiology, Notre Dame University Bangladesh (NDUB), Dhaka, Bangladesh; Islamic Azad University, IRAN, ISLAMIC REPUBLIC OF

## Abstract

Marburg virus disease (MVD), a deadly zoonotic illness in humans, is caused by the Marburg virus (MARV), a single-stranded RNA virus belonging to the *Orthomarburgvirus* genus of the *Filoviridae* family. There are currently no approved vaccines or efficient antiviral therapies for MARV, despite high morbidity and fatality rates of the virus, particularly in Africa. Recognizing its epidemic potential and its inclusion on the WHO list of blueprint priority pathogens, an immunoinformatics approach was used to design novel multi-epitope saRNA vaccine candidates (MB-saVaxI and MB-saVaxII) targeting the viral GP, NP, VP40, and VP35 via CD8 + , CD4 + , and B cell epitopes. These epitopes were derived from target protein sequences and incorporated into MB-saVaxI and MB-saVaxII after validation for antigenicity, non-allergenicity, non-toxicity, and conservation. Additionally, the selected epitopes were conjugated to suitable adjuvants via various linkers, thereby enabling the design of a potentially stable vaccine. Additionally, the designed MB-saVaxI and MB-saVaxII were incorporated into the standard saRNA model, in which ORF-2 encodes the target vaccine peptide. Moreover, biophysical analyses unveiled that the developed vaccines are basic (pI < 7), soluble (GRAVY < 0), and stable (aliphatic index < 80). Subsequently, docking of MB-saVaxI to TLR2 (−938.3) and TLR4 (−902.4), and MB-saVaxII to TLR3 (−1485.4) and TLR7 (−1543.6) demonstrated significant affinity, with detailed hydrogen-bond interactions validated by MM-GBSA and molecular dynamics simulations. Furthermore, MB-saVaxI and MB-saVaxII were effectively cloned *in-silico* into *Escherichia coli*, and immune simulations indicated that the vaccines triggered robust innate and adaptive immunity, with a sustained memory response and TH cell activation, as evidenced by IFN-γ and IL-2 production. Additionally, the mRNA structure of MB-saVaxI and MB-saVaxII remained stable throughout the expression pathway in the host. However, extensive *in-vitro* and *in-vivo* studies are needed to establish the safety and efficacy of this vaccine candidate conclusively.

## 1. Introduction

Marburg virus disease (MVD) is a severe acute haemorrhagic fever caused by Marburg virus (MARV), a highly virulent zoonotic pathogen [1,2]. It is classified within the order *Mononegavirales* and the family *Filoviridae*, a lineage composed of highly virulent, filamentous viruses [3]. Transmission of MARV occurs primarily through prolonged exposure to infected animals, including bites or direct contact with bodily fluids, excreta, droplets, or blood. Additionally, human-to-human transmission arises from close contact with infected individuals or contaminated surfaces [4]. Most commonly, transmission to humans occurs through prolonged exposure to environments such as mines or caves inhabited by colonies of fruit bats, particularly the Egyptian fruit bat (*Rousettus aegyptiacus*), which serves as the natural reservoir host [5]. Once the virus enters humans, secondary transmission occurs through contact with the secretions, blood, organs, or other body fluids of infected individuals, primarily through broken skin or mucous membranes [5].

The incubation period of MVD ranges from 3 to 21 days, during which MARV initiates replication by targeting macrophages and dendritic cells [6]. Initially, the symptoms include sudden high fever, chills, headache, myalgia, and malaise [7,8]. Within a few days, gastrointestinal manifestations such as nausea, vomiting, watery diarrhea, and abdominal pain often develop, frequently leading to dehydration and severe weight loss [3,9]. Furthermore, patients may develop maculopapular rashes and conjunctivitis; involvement of the liver and spleen can result in hepatomegaly in the advanced stage of infection [9,10]. Notably, the nervous system is also affected, and symptoms such as confusion, irritability, and seizures or convulsions indicate central nervous system involvement [9,11]. In advanced stages, the disease turns into its hemorrhagic phase, characterized by bleeding from mucous membranes and the gastrointestinal tract, as well as uncontrolled bleeding from venipuncture sites [3,12]. Ultimately, multi-organ failure and shock, alongside disseminated intravascular coagulation (DIC), lead to death [9,13]. Historically, the first recorded outbreak occurred in 1967, resulting in 31 cases and seven deaths. Subsequently, outbreaks were reported in South Africa (1975; three cases, one death), Kenya (1980; two cases, one death), and again in Kenya in 1987 with a single fatal case [1,8]. The largest and most lethal outbreak occurred in Angola during 2004–2005, involving 252 cases and resulting in 227 deaths, corresponding to a case fatality rate (CFR) of ~90%, thereby underscoring the extreme lethality of the virus [14]. Thereafter, smaller outbreaks were reported in Uganda in 2012 (15 cases, four deaths) and 2017 (3 cases, all fatal) [10]. In recent years, cases have also been detected in Guinea (2021) and Rwanda (2024), with 66 confirmed cases, 15 deaths, and 51 recoveries, resulting in a CFR of ~23% [8,15]. The most recent outbreak occurred in the United Republic of Tanzania in March 2025, with 10 cases and 10 deaths, corresponding to a 100% case fatality rate [16]. The recurring nature of MARV outbreaks underscores the urgent need for improved zoonotic surveillance, rapid diagnostic tools, and coordinated global response mechanisms to mitigate the impact of the deadly disease [8]. Due to its high mortality rate, ranging from 24% to 88% depending on outbreak conditions and case management, the World Health Organization (WHO) designated MARV as a priority pathogen in 2018 [10,17].

At the genomic level, MARV is a non-segmented, negative-sense, single-stranded RNA virus approximately 19.1kb [12]. Its genome is flanked by noncoding leader and trailer regions that contain cis-acting elements essential for replication and transcription. Moreover, it encodes seven structural proteins, including glycoprotein (GP), nucleoprotein (NP), viral protein 40 (VP40), and viral protein 35 (VP35) [1,12,18]. Collectively, these components play critical roles in transcription, replication, nucleocapsid formation, budding, host cell attachment, and immune evasion, and they function synergistically to ensure efficient viral replication and pathogenesis [12]. Specifically, the GP complex forms spikes on the viral surface, mediating viral attachment to host cells and fusion of the viral envelope with the host cell membrane [11,19]. Importantly, GP is the sole envelope protein essential for viral attachment and entry during the early stages of the viral life cycle [10,20]. Similarly, the NP encapsidates the RNA genome, forming the ribonucleoprotein complex required for transcription and translation [11,12]. Additionally, virus matrix protein VP40, manages the assembly and budding of new virions by driving structural formation at the host membrane and also suppresses host interferon signaling pathways by blocking key signaling kinases, thereby weakening antiviral defenses; mutations in VP40 can further influence host specificity and severity of disease, highlighting its role in virulence and virus spread within and between hosts [21]. Furthermore, VP35 is a multifunctional viral protein that acts as a cofactor for viral RNA polymerase, enabling efficient replication and transcription of the viral genome, and it helps the virus to evade host innate immunity by binding and masking viral RNA, which inhibits interferon production and RIG-I-like receptor signalling, making it crucial for viral survival and pathogenicity in MVD [22]. Despite extensive research, developing an effective vaccine against MARV remains a major scientific and public health challenge [6]. Several approaches have been evaluated, including live-attenuated, inactivated, DNA, RNA (mRNA technology), and subunit vaccines [7,12]. Nevertheless, live-attenuated and inactivated vaccines are commonly referred to as conventional approaches to vaccines. DNA vaccines are relatively stable and generally safer, but may require an advanced delivery system such as intramuscular electroporation [23]. In contrast, the replicase of self-amplifying (saRNA) amplifies both genomic and subgenomic RNA, yielding very high antigen levels from minimal input RNA, often allowing 10–100‑fold lower doses than conventional mRNA to reach similar expression or protection [24,25]. Additionally, saRNA vaccines provide dose-sparing benefits and elicit more sustained and potent antibody responses, thereby improving vaccine efficiency while reducing production costs [26]. Significantly, based on reverse vaccinology (RV) or *in-silico* vaccine designing, there are some potential research areas where *in-silico* vaccines have undergone an *in-vitro* trial, and have shown experimental success in the *in-vitro* or *in-vivo* [27]. Since experimental validation is expensive and time-consuming, the majority of vaccine investigations are still restricted to *in-silico* design. In this context, advances in RV and immunoinformatics, complemented by vaccinomics strategies, enable the computational prediction of epitopes and the rational design of antigens without the immediate need for pathogen culturing [6,10,28]. These *in-silico* methods accelerate vaccine discovery, reduce costs, and enhance safety, making them ideal for high-risk pathogens like MARV [6,10]. Currently, no FDA-approved vaccine or specific antiviral therapy is available for MVD [29]. In the absence of a proper approved vaccine against MARV, this study aimed to design a novel candidate saRNA vaccine using an immunoinformatics-driven approach.

## 2. Methods and materials

### 2.1. Acquisition of protein sequences

The National Center for Biotechnology Information (NCBI) (https://www.ncbi.nlm.nih.gov/) protein database was utilized to collect the amino acid sequences of the target proteins GP (accession number: YP_001531156.2), NP (accession number: YP_001531153.1), VP40 (accession number: YP_001531155.1), and VP35 (accession number: YP_001531154.1) and recorded in FASTA format [30]. These sequences were employed for the subsequent steps.

### 2.2. CD8+ and CD4 + epitopes identification

CD8+ and CD4 + epitopes from the target proteins GP, NP, VP40, and VP35 were identified using the Immune Epitope Database (IEDB) web server (https://tools.iedb.org/mhci/) (https://tools.iedb.org/mhcii/) [31]. Based on the results, 9-mer length epitopes with percentile ranks below 1 were selected as CD8 + epitopes, as low percentile ranks indicate enhanced binding affinity for Human Leukocyte Antigen (HLA) class I molecules [32]. Similarly, 15-mer-length epitopes were selected as CD4 + epitopes based on their binding strength to HLA class II molecules, as reflected by their percentile rank [33]. The IEDB is a comprehensive and curated data repository that is utilized for computational prediction and analysis of CD8 + epitopes, CD4 + epitopes [34]. Additionally, the antigenicity and allergenicity of the selected epitopes were evaluated employing VaxiJen v2.0 (https://www.ddg-pharmfac.net/vaxijen/VaxiJen/VaxiJen.html) and AllerTop v2.1 (https://www.ddg-pharmfac.net/allertop_test/) websites, respectively [35,36]. Subsequently, the ToxinPred tool (https://webs.iiitd.edu.in/raghava/toxinpred/algo.php) was implemented to assess the toxicity of the screened epitopes [37].

### 2.3. Linear B cell (LBC) epitopes identification

To identify the linear B cell epitopes of target proteins GP, NP, VP40, and VP35, the IEDB web server (https://tools.iedb.org/main/bcell/) was used [31]. Thereafter, the epitopes were evaluated for their antigenicity, allergenicity, and toxicity characteristics by implementing VaxiJen v2.0 (https://www.ddg-pharmfac.net/vaxijen/VaxiJen/VaxiJen.html), AllerTop v2.1 (https://www.ddg-pharmfac.net/allertop_test/), and ToxinPred (https://webs.iiitd.edu.in/raghava/toxinpred/algo.php) tools, respectively [35–37].

### 2.4. Analysis of epitope conservation

The IEDB Epitope Conservancy Analysis Tool (http://tools.iedb.org/conservancy/) was utilized to assess the conservancy of the chosen epitopes [38]. Each selected CD8 +, CD4 +, and linear B cell epitope was analyzed in comparison with homologous protein sequences obtained from UniProt (https://www.uniprot.org/) searches of GP, NP, VP40, and VP35 [39]. The analysis used a threshold of ≥100% sequence identity to ensure only fully conserved epitopes were chosen.

### 2.5. Population coverage analysis

HLA alleles exhibit extensive polymorphism due to their varying frequencies across geographically and ethnically distinct groups, making it crucial to determine their distribution when developing a potent vaccine [40,41]. Thus, the IEDB population coverage analysis tool (http://tools.iedb.org/population/) was employed to evaluate the HLA allele distribution for the selected CD8+ and CD4 + epitopes, both independently and in combination, among various populations [42].

### 2.6. Vaccine backbone preparation

The MB-saVaxI and MB-saVaxII were designed from the 5’ cap to the poly(A) tail features two open reading frames (ORFs) connected by a sub-genomic promoter (SGP) from the Venezuelan equine encephalitis virus. The construct begins with a 5’ m2 7,2′ − OGppS pG cap analog to improve RNA stability and translation. The 5’ untranslated region (5’ UTR) includes human β-globin (Gene ID: 3043) from NCBI. Then, the first open reading frame (ORF1) encoded the non-structural proteins nsP1-nsP4, constituting the RNA-dependent RNA polymerase (RdRp) complex (NCBI Gene ID: 2652925; Location: 1–7526), terminated by a stop codon. The VEEV 26S subgenomic promoter (SGP) was inserted downstream of ORF1 to drive expression of the Antigen cassette. A Kozak sequence was placed upstream of ORF2 to enhance translation. ORF2 consisted of the tPA signal peptide (UniProt ID: P0 process 0750) fused with the proteins GP, NP, VP40, and VP35. Apart from these, an adjuvant (sp|P9WHE3|RL7_MYCTU Large ribosomal subunit protein bL12 OS = *Mycobacterium tuberculosis*) was integrated into the N-terminus of the construct and helps generate a lasting immunological response and robust immunogenicity [43]. EAAAK, a rigid helical linker that facilitates hydrogen bonding and enables effective separation of functional regions, was used to link the adjuvant to the construct [44]. Similarly, the linkers AAY, GPGPG, and KK were used to ligate the CD8 +, CD4 +, and linear B cell epitopes, respectively, to construct the vaccines. The ORF2 was then followed by the MHC-I trafficking domain (MITD) (UniProt ID: Q8WV92), to enhance antigen presentation, and a stop codon. Besides, the vaccines were finalized by 3’ UTR (NCBI Gene ID: 3039) to boost mRNA stability and translation, and a poly(A) tail with about 120 adenine nucleotides for additional stabilization [45].

### 2.7. Post-synthetic protein modification analysis

In order to evaluate the modification of the MB-saVaxI and MB-saVaxII, various analyses were performed. Phosphorylation, Glycosylation, and acetylation were predicted using the NetPhos 3.1, NetNGlyc 1.0, and NetAct 1.0 tools, respectively (http://www.cbs.dtu.dk/services/) [45].

### 2.8. Biophysical features evaluation

The Expasy ProtParam website (https://web.expasy.org/protparam/) was employed to assess the biophysical features of the MB-saVaxI and MB-saVaxII construct [46]. The website calculated the instability index, isoelectric point (pI), aliphatic index, grand average of hydropathicity (GRAVY), total amino acid count, molecular mass, and the overall counts of positively and negatively charged amino acid residues. Moreover, the Scratch (SOLpro) (https://scratch.proteomics.ics.uci.edu/) and Protein-Sol (https://protein-sol.manchester.ac.uk/) tools were utilized to assess the solubility of the vaccines [47,48].

Afterwards, the ability of the vaccines to induce allergic response was predicted by the AllerTop v2.1 website (https://www.ddg-pharmfac.net/allertop_test/) [36]. Similarly, the VaxiJen v2.0 (https://www.ddg-pharmfac.net/vaxijen/VaxiJen/VaxiJen.html) and ToxinPred (https://webs.iiitd.edu.in/raghava/toxinpred/algo.php) websites were implemented to predict the antigenicity and toxicity of the vaccine, respectively [35,37].

### 2.9. Secondary (2˚) structure determination

The 2˚ structure of the MB-saVaxI and MB-saVaxII were modeled employing GOR4 (https://npsa-prabi.ibcp.fr/cgi-bin/npsa_automat.pl?page=/NPSA/npsa_gor4.html), SOPMA (https://npsa.lyon.inserm.fr/cgi-bin/npsa_automat.pl?page=/NPSA/npsa_sopma.html), and PSIPRED web servers (https://bioinf.cs.ucl.ac.uk/psipred/) [49–51]. The GOR4 server predicts secondary structure of proteins according to the information theory, as well as probability factors obtained from empirical studies of known 3˚ structures determined by X-ray crystallography [52,53]. Moreover, the SOPMA server utilizes consensus estimation among numerous alignments to predict secondary structures such as beta strands, random coils, and alpha helices, whereas PSIPRED predicts secondary structures by applying a neural network algorithm with an accuracy of 82% [52–54].

### 2.10. Tertiary (3˚) structure determination, stabilization, refinement, and quality evaluation

The I-TASSER server (https://zhanggroup.org/I-TASSER/) was utilized to determine the 3˚ structure of the MB-saVaxI and MB-saVaxII [55]. The server employs multiple threading alignment methods to develop a structure and an iterative fragment assembly simulation approach to obtain function by aligning the structure with the function of known proteins in the data repository [55]. Afterwards, disulfide engineering was performed using the Disulfide by Design 2.13 website (http://cptweb.cpt.wayne.edu/DbD2/), which uses native conformations and relies on methods developed for protein fold identification to enhance structural stability by identifying residue pairs that can be changed to cysteine and form new disulfide bonds in the structure [56,57]. Furthermore, to refine the MB-saVaxI and MB-saVaxII, the GalaxyWEB tool (https://galaxy.seoklab.org/) was employed [58]. In addition, the ProSA (https://prosa.services.came.sbg.ac.at/prosa.php), RamPlot (https://www.ramplot.in/), and SAVES v6.1 (https://saves.mbi.ucla.edu/) websites were used for validation and quality assessment [59–61]. The ProSA web server estimated and derived the Z score, whereas the SAVES v6.1 web server measured the ERRAT score, and RamPlot generated a Ramachandran plot [62].

### 2.11. Structural B cell epitope determination

Structural B cell epitopes resemble pathogen structural features, thereby eliciting enhanced immunogenic response [63]. The Ellipro website (http://tools.iedb.org/ellipro/) was used to identify structural B cell epitopes, as these are geometrically proximal to the 3˚ structure of the immunogen [64,65]. This server utilizes protein conformation approximated as an ellipsoid, proximal residues aggregation depending on their PI values, and estimation of the residue protrusion index (PI) methods to predict these epitopes [64].

### 2.12. Molecular docking within the vaccines and TLRs

Toll-like receptors (TLRs) are a major type of pattern recognition receptors (PRRs) that are expressed by numerous immune cells and recognize various pathogen-associated molecular patterns (PAMPs). They promote immune cell migration to the vaccine administration site and enhance antigen processing and presentation on HLA class I and II molecules, thereby eliciting both innate and adaptive immune responses [66,67]. Hence, the Protein Data Bank (PDB) website (www.rcsb.org) was employed to acquire 3˚ structures of human TLR2 (PDB ID: 2Z7X), TLR3 (PDB ID: 7C76), TLR4 (PDB ID: 3FXI), and TLR7 (PDB ID: 7CYN) receptors [68]. Moreover, the ClusPro 2.0 web server (https://cluspro.org/login.php?redir=/queue.php) was implemented to assess docking between TLR2 and TLR4 with MB-saVaxI, and TLR3 and TLR7 with MB-saVaxII, accordingly [69]. Afterwards, the visualization of complexes and determination of interactions between molecules were assessed using PyMOL software and PDBsum web (https://www.ebi.ac.uk/thornton-srv/databases/pdbsum/), correspondingly [70,71].

### 2.13. Molecular docking of T cell epitopes and HLA molecules

The peptides presented by HLA class I and II molecules are recognized by CD8+ and CD4 + T cells, respectively [72,73]. The PDB server (www.rcsb.org) was utilized to retrieve HLA class I (PDB ID: 1dlh) and HLA class II (PDB ID: 2xpg) molecules [68]. Thereafter, docking of CD8+ and CD4+ with HLA class I and II molecules, accordingly, was performed employing the HDOCK website (http://hdock.phys.hust.edu.cn/) [74].

### 2.14. Negative free energy calculation (MM-GBSA)

To assess the binding free energy of MB-mVaxI-TLR2, MB-mVaxI-TLR4, MB-saVaxII-TLR3, and MB-saVaxII-TLR7 complexes, the Molecular Mechanics and Generalized Born Surface Area (MM-GBSA) method was utilized, which comprises generalized born solvation energy (GB), Van der Waals forces (VdW), electrostatic interactions (ELE), and polar and nonpolar parts [75]. This was carried out by implementing the HawkDock V2 server (https://cadd.zju.edu.cn/hawkdock/) [76].

### 2.15. Molecular dynamics (MD) simulation

MD simulation was conducted to assess the dynamic features, behavior, intermolecular interaction profiles, and stability of the MB-saVaxI-TLR complexes [77]. This was carried out using the Maestro 13.5 software program of the Schrödinger suite 2023−1. Furthermore, the Protein Preparation Wizard was utilized to preprocess the docked MB-saVaxI-TLR complexes [78]. A 200 ns simulation was run using the Desmond software of Schrödinger to evaluate the interaction of MB-saVaxI-TLR complexes under a physiological environment [79]. To maintain a fixed volume, an orthorhombic periodic boundary box shape (10 × 10 × 10 **Å**³) was used within the TIP3P water model, and a physiological salt concentration of 0.15 M was also maintained by inclusion of Na+ and Cl^-^ ions to neutralize the solvated system [80]. Moreover, to run the simulation, NTP conditions at 1.01325 bar and 310 K were implemented along with the OPLS-4 force field to minimize and equilibrate the system [78]. Following the relaxation of the system, the last production cycle was performed at an energy and recording intervals of 1.2 and 200 picoseconds, respectively.

### 2.16. Codon optimization and computational cloning

Optimization of the DNA sequence of MB-saVaxI and MB-saVaxII by reverse translation using *E. coli* strain k12 was carried out employing the Java Codon Adaptation Tool (JCat) website (https://www.jcat.de/Start.jsp) [81]. From the result, GC content and Codon Adaptation Index (CAI) were also collected to assess expression levels. Then, the optimized sequence was integrated into the pcDNA™3.1(+) vector, and restriction sites (BstZ17I and EcoRV) were inserted for computational cloning employing the SnapGene software (https://www.snapgene.com/free-trial) [82].

### 2.17. Immunological response simulation

The simulation of the immunological response of the MB-saVaxI and MB-saVaxII was carried out using the C-IMMSIM tool (https://kraken.iac.rm.cnr.it/C-IMMSIM/index.php), where three doses of the vaccine were delivered with a 28-day interval [83]. Also, the simulation steps and volume were set to 1000 and 50, respectively, while the time-steps were fixed to 1, 84, and 168, where one time-step is the same as 8 hours. This tool predicts the antibody and cell-mediated immunological response of the host to the vaccine [84].

### 2.18. Modelling of the 2˚ structure of mRNA vaccine

Using the optimized DNA sequence, an RNA sequence of the MB-saVaxI and MB-saVaxII was produced utilizing the Biomodel website (https://biomodel.uah.es/en/lab/cybertory/analysis/trans.htm) [85]. Next, the RNAfold website (http://rna.tbi.univie.ac.at/cgi-bin/RNAWebSuite/RNAfold.cgi) was implemented to model the 2˚ structure with the retrieved sequence [86]. In addition, based on the thermodynamics principle, the minimum free energy (MFE) was also estimated by the website.

## 3. Results

### 3.1. Acquisition of protein sequences

The FASTA format of the amino acid sequence of target proteins GP, NP, VP40, and VP35 was obtained by implementing the NCBI Database (Fig 1).

**Fig 1:**
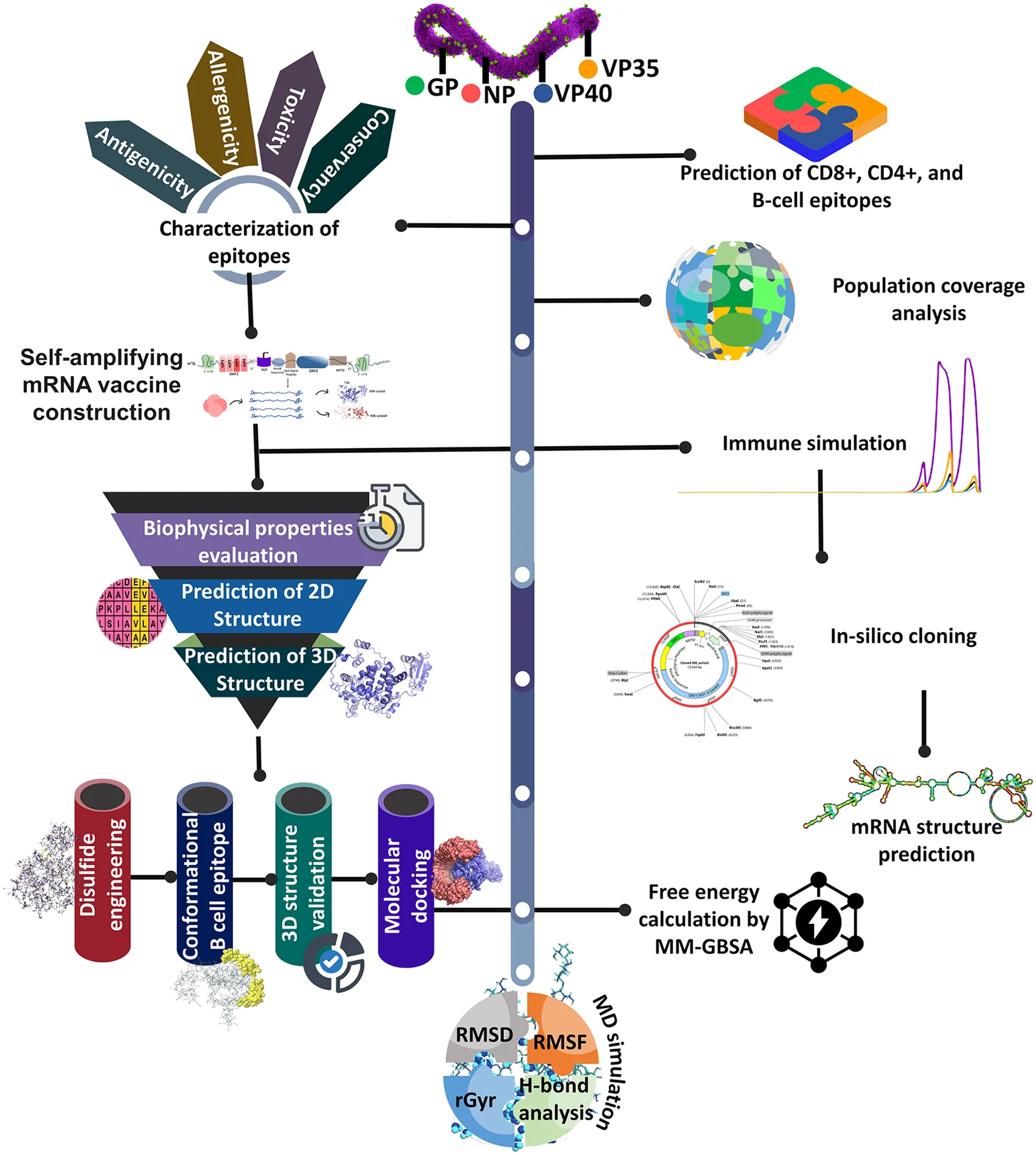
The schematic representation of the study methodology for designing the MB-saVaxI and MB-saVaxII against MARV.

### 3.2. CD8+ and CD4 + epitopes identification

Using the IEDB MHC I data repository for selected human HLA class I alleles, 9-mer length CD8 + epitopes were identified, and 6 epitopes were selected for MB-saVaxI and 8 epitopes for MB-saVaxII based on the percentile rank (<1.00) (Tables 1 and 2). In contrast, 15-mer length CD4 + epitopes were identified employing the IEDB MHC II database, and among them, 6 epitopes were chosen for MB-saVaxI and 8 epitopes for MB-saVaxII (Tables 3 and 4). Nevertheless, the most antigenic, non-allergenic, and non-toxic epitopes were used in the construction of the MB-saVaxI and MB-saVaxII. However, one CD4 + epitope from MB-saVaxII (GLYPQLSAI) was predicted to be allergenic despite demonstrating antigenicity.

**Table 1:** CD8 + epitopes from the protein sequences and their ability to induce immunity of the MB-saVaxI.

Proteins	Epitope	Percentile rank	Antigenic property	Allergenic property	Toxic property
GP	FGPGIEGLY	0.88	0.9714 (Yes)	No	No
	LLLLSIAVL	0.86	1.6465 (Yes)	No	No
NP	AIALGVATA	0.28	1.0957 (Yes)	No	No
	QLSAIALGV	0.3	1.4299 (Yes)	No	No
VP40	VAALLTGSY	0.08	0.4533 (Yes)	No	No
VP35	AQVLSKIAY	0.01	0.4700 (Yes)	No	No

**Table 2:** CD8 + epitopes from the protein sequences and their ability to induce immunity of the MB-saVaxII.

Proteins	Epitope	Percentile rank	Antigenic property	Allergenic property	Toxic property
GP	DDLAAGLSW	0.31	1.2586 (Yes)	No	No
	HAQGIALHL	0.45	0.5231 (Yes)	No	No
NP	APFARVLNL	0.12	0.6130 (Yes)	No	No
	GLYPQLSAI	0.08	0.9509 (Yes)	No	No
VP40	AYANPTLSA	0.87	0.9287 (Yes)	No	No
	DMFSVKEGM	0.91	1.0511 (Yes)	No	No
VP35	AYKSGKSGA	0.93	1.0199 (Yes)	No	No
	KSGKSGAFL	0.94	1.1583 (Yes)	No	No

**Table 3:** CD4 + epitopes from the protein sequences and their ability to stimulate the immunity of the MB-saVaxI.

Proteins	Epitope	Percentile rank	Antigenic property	Allergenic property	Toxic property
GP	TEERTFSLINRHAID	0.13	0.7221 (Yes)	No	No
	EERTFSLINRHAIDF	0.6	0.9379 (Yes)	No	No
NP	HEHQEIQAIAEDDEE	0.13	0.4223 (Yes)	No	No
	LNLSGINNLEHGLYP	0.85	0.4105 (Yes)	No	No
VP40	ITPNYVGDLNLDDQF	0.4743	1.1032 (Yes)	No	No
VP35	EGENAQAALTRLSRT	0.18	0.4164 (Yes)	No	No

**Table 4:** CD4 + epitopes from the protein sequences and their ability to stimulate the immunity of the MB-saVaxII.

Proteins	Epitope	Percentile rank	Antigenic property	Allergenic property	Toxic property
GP	SGQKVADSPLEASKR	0.52	0.6619 (Yes)	No	No
	AKTCYNISVTDPSGK	0.34	0.8031 (Yes)	No	No
NP	APFARVLNL	0.01	0.4252 (Yes)	No	No
	GLYPQLSAI	0.64	0.5531 (Yes)	Yes	No
VP40	AYANPTLSA	0.46	1.2177 (Yes)	No	No
	DMFSVKEGM	0.33	0.6950 (Yes)	No	No
VP35	AYKSGKSGA	0.34	0.9643 (Yes)	No	No
	KSGKSGAFL	0.46	1.0699 (Yes)	No	No

### 3.3. Linear B cell (LBC) epitopes identification

Using the IEDB web server, 6 epitopes from the proteins (GP, NP, VP40, and VP35) were determined and selected. Moreover, the epitopes were likely to be antigenic, non-toxic, and non-allergenic; however, two epitopes from MB-saVaxII (VGEQYQQL and LRAVPPNPTID) were predicted to be allergenic (Tables 5 and 6). These epitopes were then utilized for the design of MB-saVaxI and MB-saVaxII.

**Table 5:** LBC epitopes from the protein sequences and their immunogenicity of MB-saVaxI.

Protein	Epitope	Start	Antigenic property	Allergenic property	Toxic property
GP	IDFDPVPNTKTIFDESSSSGASAEEDQHASPN	456	0.9304 (Yes)	No	No
	QKVADSPLEAS	57	0.6368 (Yes)	No	No
NP	YQNPVSAKELQADWPDMSFD	667	0.9277 (Yes)	No	No
VP40	MASSSNYN	1	0.7270 (Yes)	No	No
VP35	YKSGKSG	240	1.4520 (Yes)	No	No

**Table 6:** LBC epitopes from the protein sequences and their immunogenicity of MB-saVaxII.

Protein	Epitope	Start	Antigenic property	Allergenic property	Toxic property
GP	IDFDPVPNTKTIFDESSSSGASAEEDQHASPN	456	0.9304 (Yes)	No	No
NP	TKPTAPHVR	11	0.8857 (Yes)	No	No
	VGEQYQQL	318	0.9002 (Yes)	Yes	No
VP40	PENFPLN	278	0.6244 (Yes)	No	No
VP35	LRAVPPNPTID	300	0.6947 (Yes)	Yes	No

### 3.4. Analysis of epitope conservation

The conservation analysis revealed that the selected CD8 + , CD4 + , and B cell epitopes from proteins GP, NP, VP40, and VP35 were highly conserved among homologous protein sequences. The conservancy percentage of epitopes ranges from 21.05% to 94.74% for GP, 50% to 100% for NP, 88.89% to 94.44% for VP40, and 87.50% to 100% for VP35 for MB-saVaxI. Additionally, the percentage of epitopes ranges from 21.05% to 94.74% for GP, 42.31% to 100% for NP, 94.44% to 100% for VP40, and 91.67% to 100% for VP35 for MB-saVaxII (Tables 1 and 2 in S1 File).

### 3.5. Population coverage analysis

The IEDB population coverage analysis tool was utilized to evaluate the possibility of binding between chosen epitopes and HLA alleles among various regions globally. Remarkably, the world population coverage of the combined alleles for MB-saVaxI and MB-saVaxII was predicted to be 98.98% and 96.21%, respectively. Also, the highest combined coverage of MB-saVaxI was found in Central America (99.75%). Furthermore, West Africa and North America also showed exceptional population coverage of 99.5%. On the other hand, East Africa (99.06%) had the highest coverage of MB-saVaxII. Although the lowest population coverage of MB-saVaxI and MB-saVaxII was in South Africa (64.63%) and Central America (55.01%), respectively (Fig 2, Tables 3 and 4 in S1 File).

**Fig 2:**
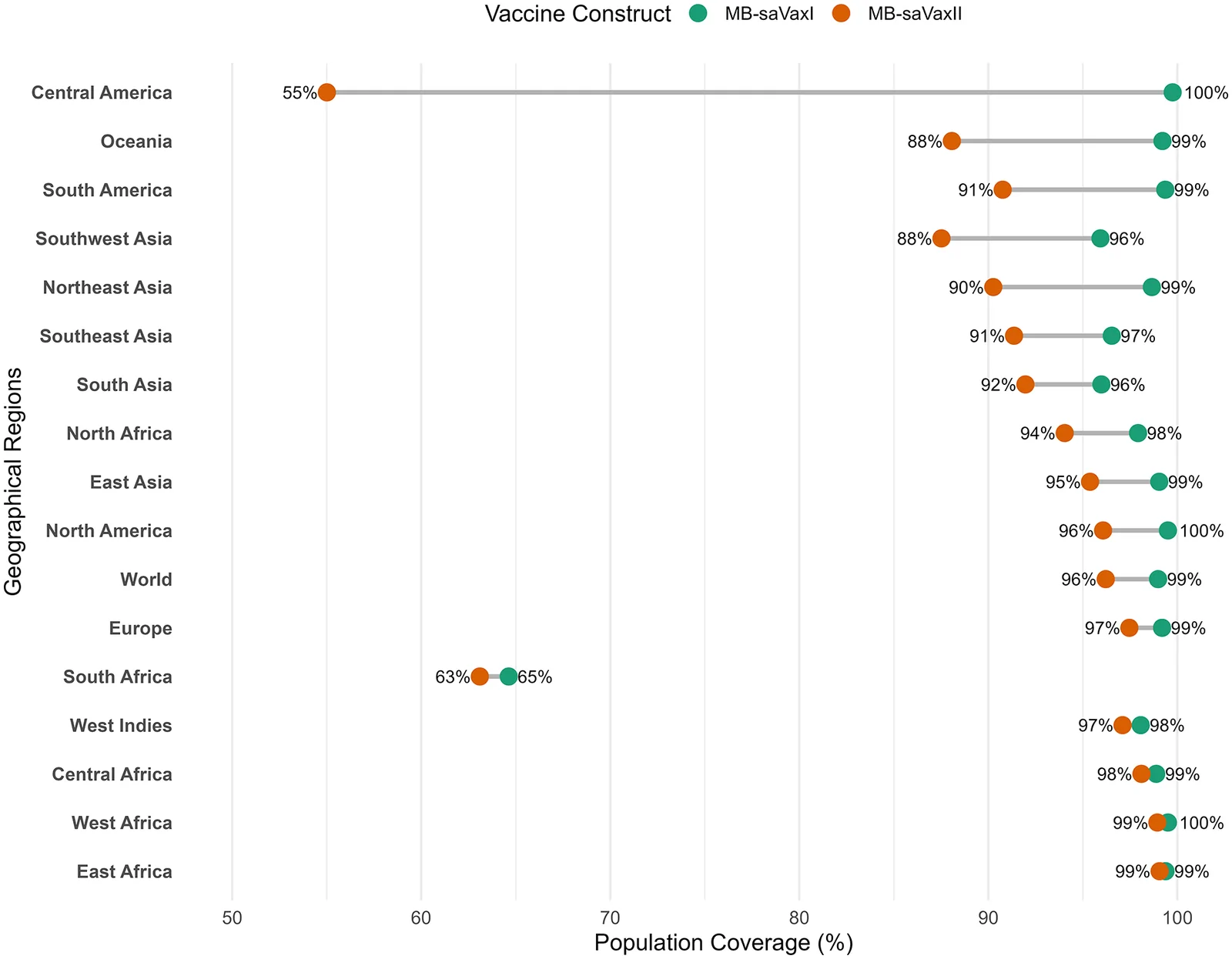
The Dumbbell plot represents the distribution of population coverage across the combined CD8+ and CD4 + epitopes for MB-saVaxI and MB-saVaxII.

### 3.6. Vaccine backbone preparation

To prepare the MB-saVaxI and MB-saVaxII backbones, selected CD8+ and CD4 + epitopes and linear B cell epitopes were compiled, along with numerous linkers such as EAAAK, AAY, GPGPG, and KK, with the EAAAK linker used to connect the adjuvant. The multi-epitope sequence (ORF2) was then incorporated into an saRNA backbone. To assure optimal replication, translation, and presentation, the comprehensive MB-saVaxI and MB-saVaxII architecture was constructed in a 5’ to 3’ orientation as follows by a 5’ cap (m7G), 5’ UTR, ORF1 (encoding the nsP1-nsP4 replicase complex), a subgenomic promoter (SGP), a Kozak sequence, a tPA signal peptide, the multi-epitope antigen sequence (ORF2), an MITD trafficking signal, a 3’ UTR, and a poly(A) tail (Fig 3 and Table 1 in S6 File).

**Fig 3:**
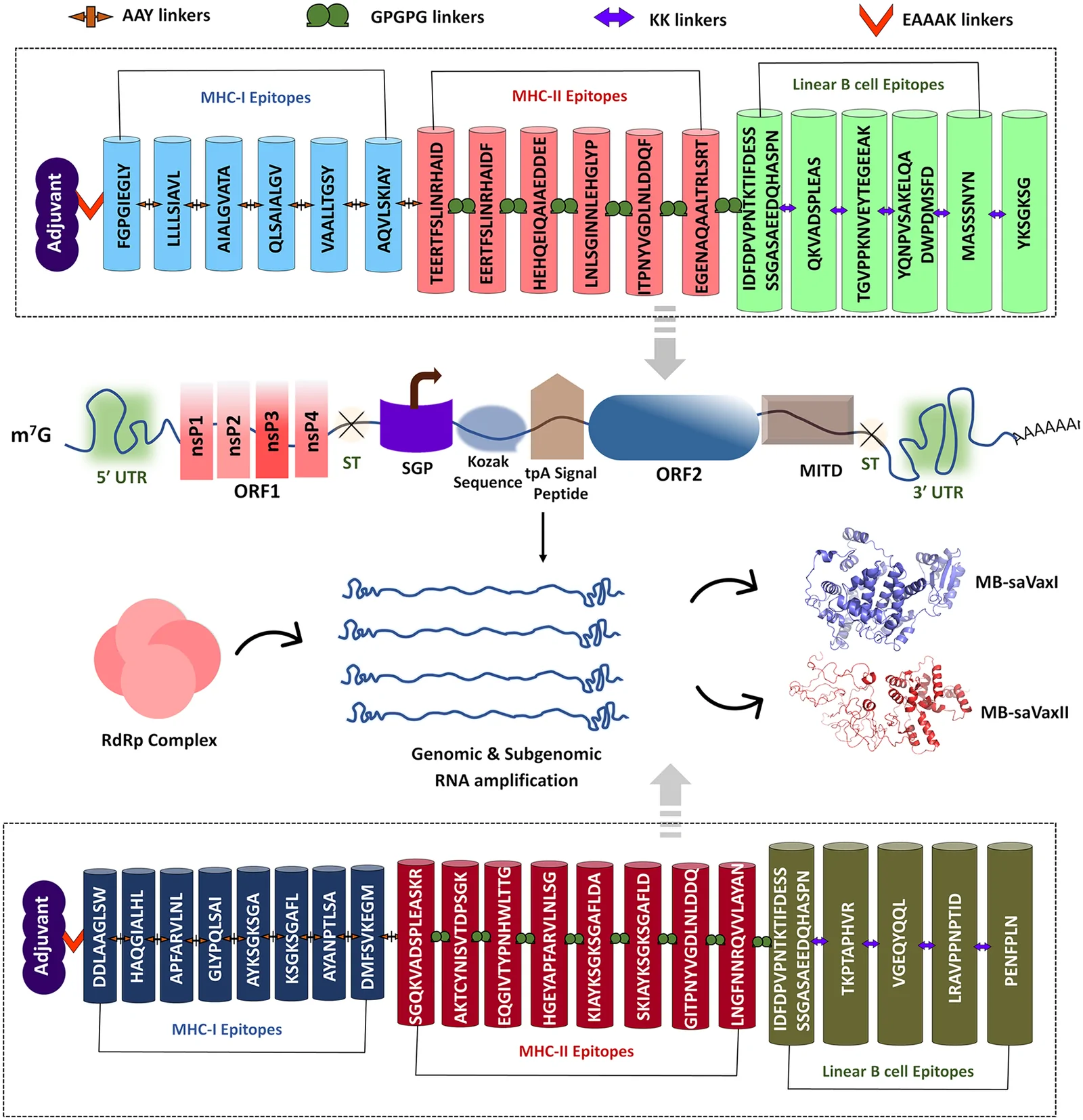
Schematic representation of MB-saVaxI and MB-saVaxII, and the translation of the construct into nsP1-4 proteins, generates an RdRP complex that amplifies both genomic and subgenomic RNAs, resulting in the accumulation of the antigen.

### 3.7. Post-synthetic protein modification analysis

The designed vaccine construct was evaluated for potential post-synthetic protein modification analysis. N-glycosylation analysis indicated that the MB-saVaxI has an N-glycosylation site at 269 NLSG position, and MB-saVaxII has an N-glycosylation site at the 211 NPTL, 257 NISV, 303 NLSG, and 453 NPTI positions. Moreover, phosphorylation profiling with NetPhos 3.1 identified 22 and 18 phosphorylation sites distributed across serine, threonine, and tyrosine residues, in MB-saVaxI and MB-saVaxII, respectively. In contrast, MB-saVaxI and MB-saVaxII showed no acetylation site.

### 3.8. Biophysical features evaluation

The biophysical features were evaluated using the ExPASy ProtParam website, which revealed that MB-saVaxI and MB-saVaxII contained 6449 and 6837 atoms, respectively, and had molecular weights of 45928.45 and 48669.81 Da, respectively. MB-saVaxI and MB-saVaxII contained 438 and 472 amino acids, respectively. Additionally, the total number of positively charged residues (Arg + Lys) was 41 and 48, while negatively charged residues (Asp + Glu) numbered 63 and 50 in MB-saVaxI and MB-saVaxII, respectively. Both vaccines were indicated as stable and highly thermostable, with instability indexes of 32.01 and 24.15, and aliphatic indices of 82.40 and 74.22 for MB-saVaxI and MB-saVaxII, respectively. Furthermore, MB-saVaxI and MB-saVaxII had pI values of 5.00 and 6.65 and GRAVY scores of −0.282 and −0.298, respectively. Solubility was assessed using SOLpro (probability MB-saVaxI: 0.946257, MB-saVaxII: 0.944209) and Protein-Sol (0.713 and 0.420). Both vaccines were found to be antigenic (MB-saVaxI: 0.6051, MB-saVaxII: 0.5811) and non-allergenic (Table 7).

**Table 7:** Evaluation of biophysical features of the vaccine constructs.

Parameter	Value	
	**MB-saVaxI**	**MB-saVaxII**
Amino acid count	438	472
Molecular mass	45928.45 Da	48669.81 Da
Isoelectric point (pI)	5.00	6.65
Total number of negatively charged residues (Asp + Glu)	63	50
Total number of positively charged residues (Arg + Lys)	41	48
Instability index (II)	32.01	24.15
Aliphatic index	82.40	74.22
GRAVY	−0.282	−0.298
Solubility (SOLpro)	0.946257	0.944209
Protein-Sol	0.713	0.420
Antigenicity	0.6051 (Yes)	0.5788 (Yes)
Allergenicity	No	No

### 3.9. Secondary (2˚) structure determination

GOR4, SOPMA, and PSIPRED web servers were employed to estimate the 2˚ structure of both vaccines. Based on GOR4, the random coils, extended strands, and alpha helices of the structure were 42.01%, 5.02%, and 52.97%, respectively, for the MB-saVaxI. Whereas, for MB-saVaxII, the values were 44.07%, 13.14%, and 42.80%, correspondingly. Meanwhile, the percentages predicted by SOPMA were 40.64%, 9.13%, and 46.12% for MB-saVaxI and 46.40%, 14.62%, and 35.38% for MB-saVaxII, respectively (Fig 4). In addition, the PSIPRED web server used a 3-state strategy to assess secondary-structure features such as coils, strands, and helices (Fig 5).

**Fig 4:**
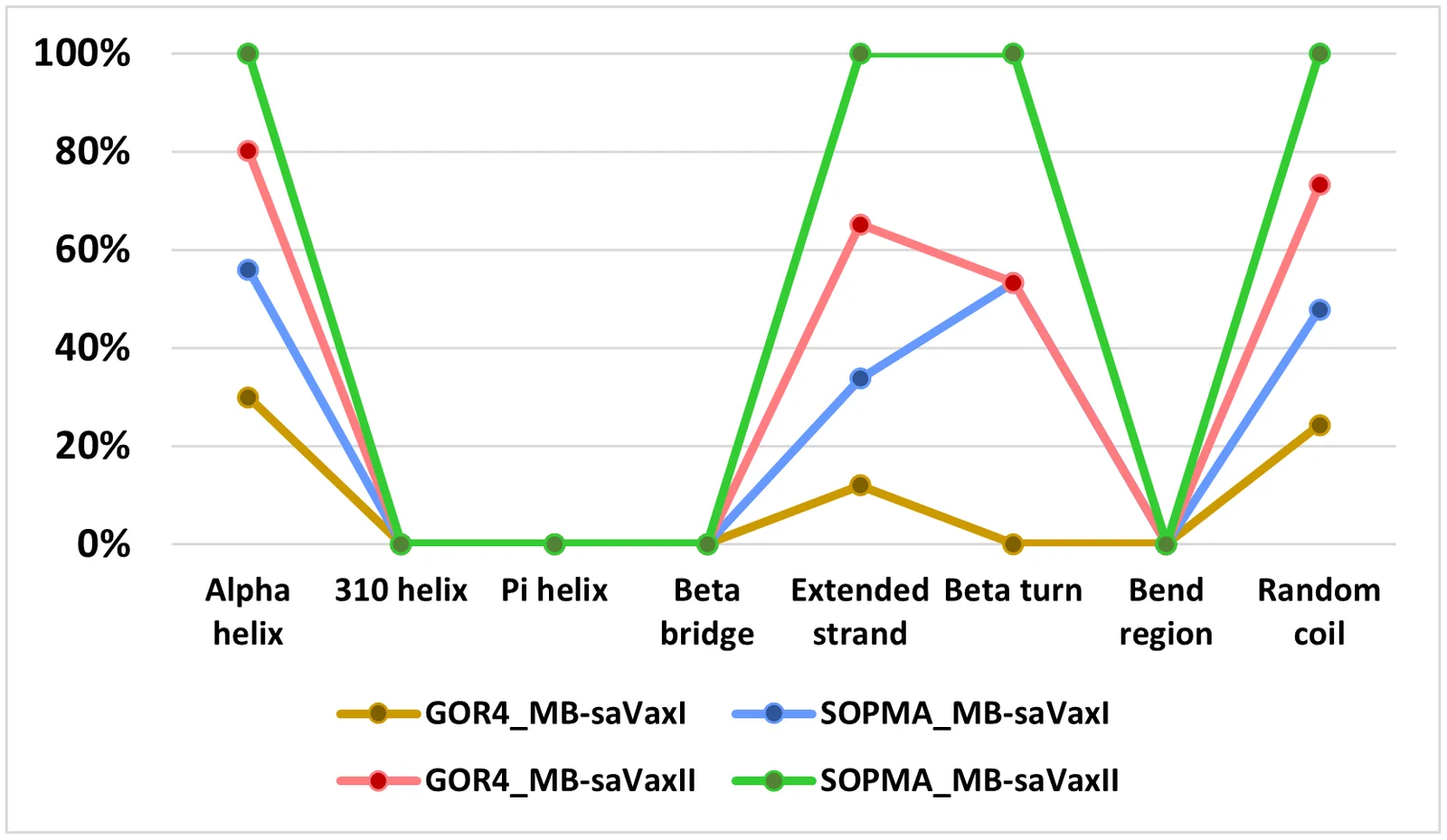
The 2˚structure of the MB-saVaxI and MB-saVaxII predicted by GOR4 and SOPMA.

**Fig 5:**
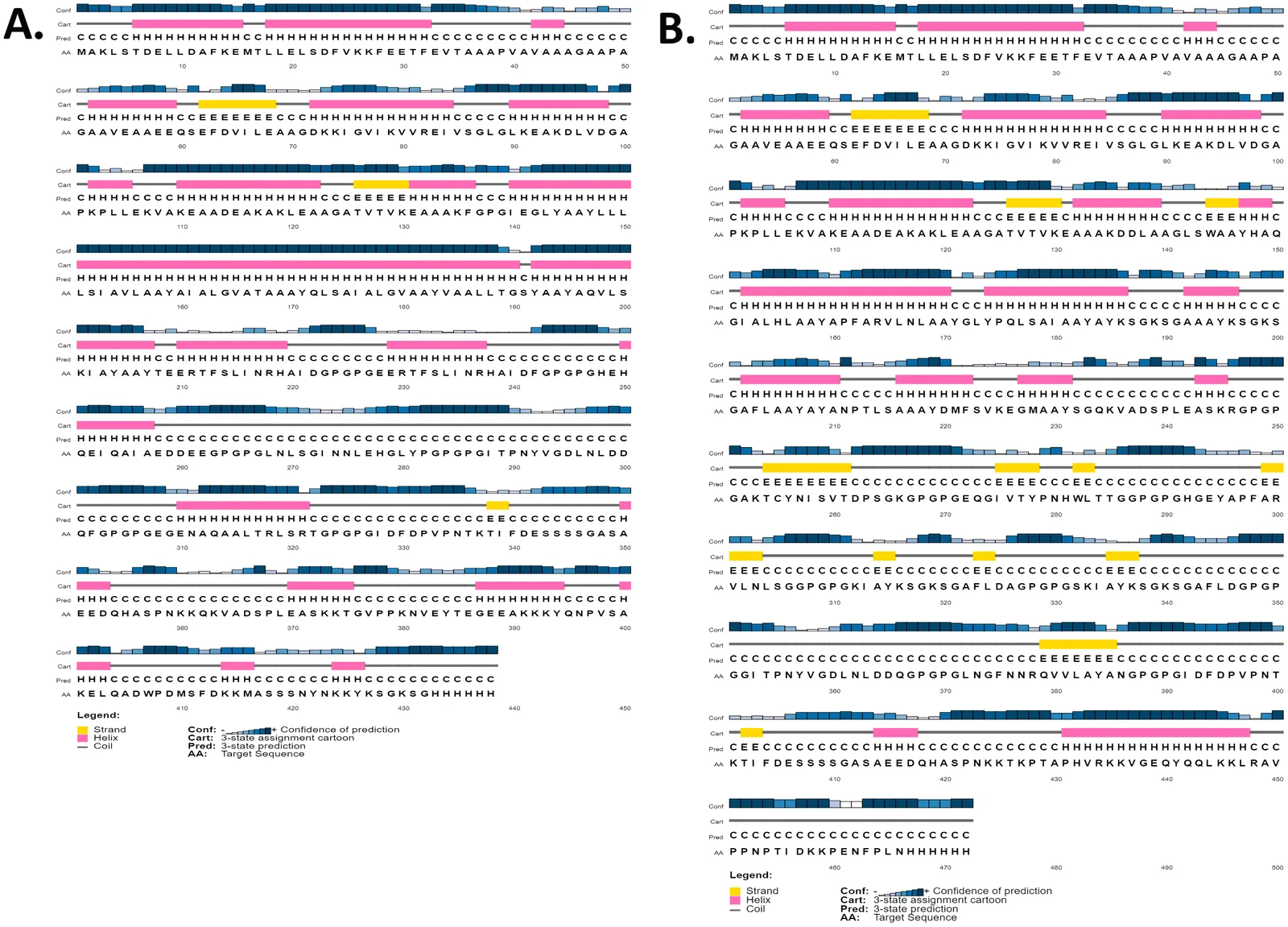
The PSIPRED predicted structures show helices, strands, and coils along the amino acid sequence. The beta-sheets are signified by the yellow color, the helical structure is represented by the pink color, and the coil configuration is denoted by the gray hue.

### 3.10. Tertiary (3˚) structure determination, stabilization, refinement, and quality evaluation

The I-TASSER generated 5 models, and based on optimal C-score, TM-score, and minimal root-mean-square deviation (RMSD), the fourth and third model was chosen for MB-saVaxI and MB-saVaxII, respectively. Then, disulfide engineering was performed using Disulfide by Design v2.13 to increase the structure’s conformational stability. In MB-saVaxI, the server predicted 34 amino acid pairs as viable candidates for forming disulfide bonds, of which 4 were selected based on energy and Chi3 values. Subsequently, the residues pairs where eight mutations were added was ALA 37-PRO 49 (energy = 3.56 kcal/mol and Chi3 = +80.51), GLY 51-ALA 162 (energy = 3.93 kcal/mol and Chi3 = −76.04), LEU 143-VAL 184 (energy = 4.59 kcal/mol and Chi3 = +77.78), and ALA 146-LEU 188 (energy = 4.16 kcal/mol and Chi3 = −72.09) (Fig 6D and Table 5 in S1 File). Similarly, 44 amino acid pairs were predicted in MB-saVaxII, among which 4 were selected to introduce disulfide bonds. The energy and Chi3 values of these 4 pairs were ALA 100-PRO 101 (energy = 1.12 kcal/mol and Chi3 = + 92.27), ALA 243-LYS 316 (energy = 2.5 kcal/mol and Chi3 = +88.07), LYS 253-THR 254 (energy = 0.78 kcal/mol and Chi3 = −76.35), and ASN 466-HIS 467 (energy = +1.66 kcal/mol and Chi3 = +86.15) (Fig 6H and Table 6 in S1 File). Furthermore, the GalaxyWEB server was used to refine the 3˚ structure and to estimate the RMSD score and MolProbity, which were 0.328 and 2.087 for MB-saVaxI and 0.277 and 1.918 for MB-saVaxII, respectively. Moreover, the quality factor of MB-saVaxI and MB-saVaxII assessed using ERRAT analysis was 86.5707 and 77.686, correspondingly. Also, a Z-score of −7.75 and −5.82 for MB-saVaxI and MB-saVaxII was predicted by the ProSA web server (Fig 6B and 6F). Additionally, the RamPlot website was implemented to generate a Ramachandran plot for the energy-minimized structure, which illustrated, in MB-saVaxI, the most-favoured regions and allowed regions, consisting of 93.35% and 4.82% amino acid residues, accordingly. Although 1.84% residues were also observed in the disallowed regions (Fig 6C and 6G). Conversely, the amino acid residues of MB-saVaxII in most-favoured regions, allowed regions, and disallowed regions were 91.70%, 7.02%, and 1,28%, respectively. These characteristics were also assessed for the refined AlphaFold3-predicted model and compared with those of the refined I-TASSER model (Table 9 in S1 File).

**Fig 6:**
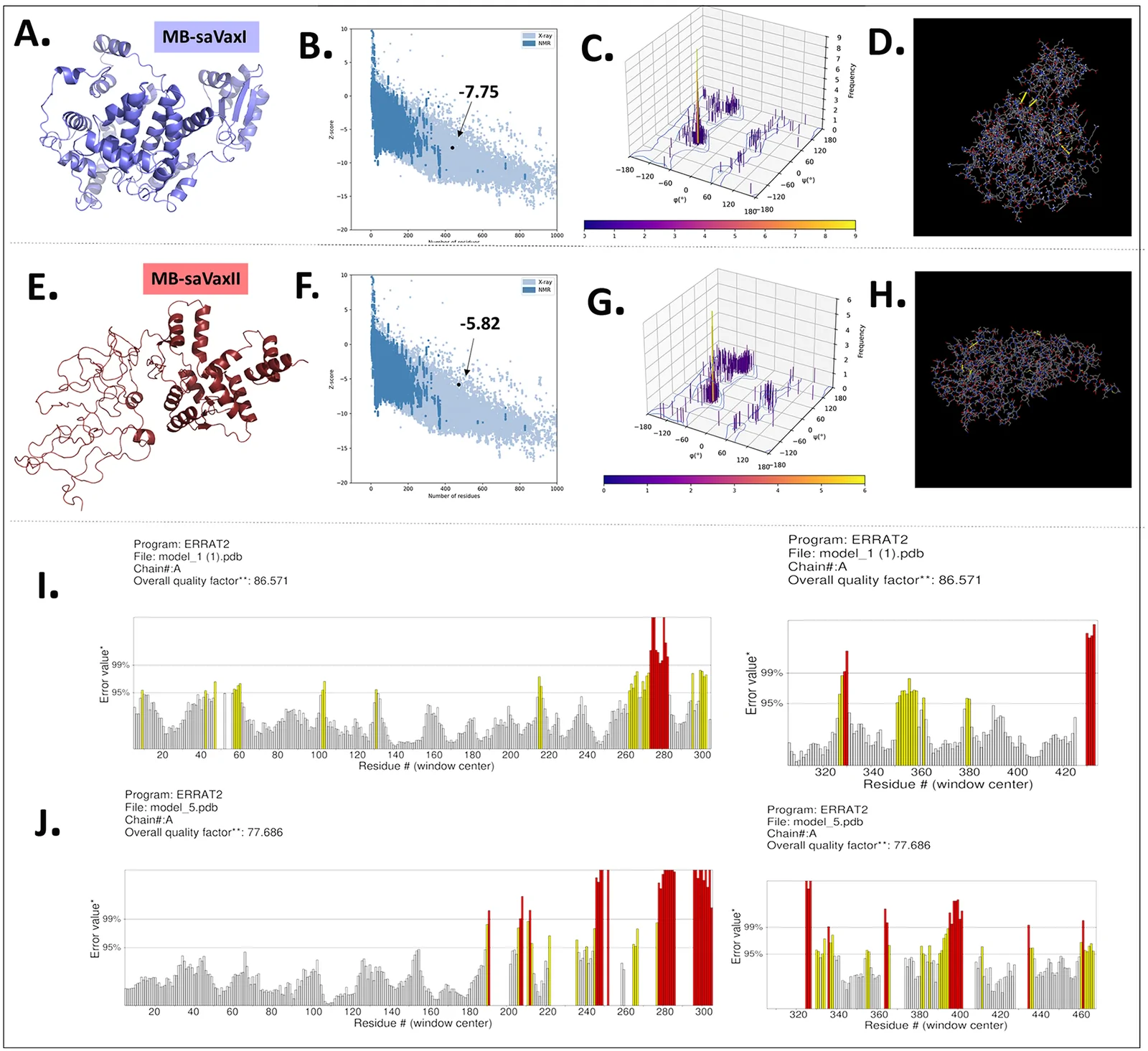
Analysis of predicted and refined saRNA 3˚ model. The validated 3˚ construct (A and E) was determined by Z score (B and F), Ramachandran plot (C and G), disulfide engineering (D and H), and ERRAT score (D) for MB-saVaxI and MB-saVaxII.

### 3.11. Structural B cell epitope determination

The Ellipro website identified 9 structural B cell epitopes among the 438 amino acid residues of MB-saVaxI with a protrusion index (PI) values ranging from 0.535 to 0.887. The peptides with the highest PI (0.887) and lowest PI (0.535) consisted of 8 and 16 amino acid residues, respectively (Fig 7, Tables 7 and 8 in S1 File). Likewise, the protrusion index (PI) values of structural B cell epitopes of MB-saVaxII ranged from 0.839 to 0.548, with the highest and lowest PI peptides comprising 39 and 9 amino acid residues, respectively.

**Fig 7:**
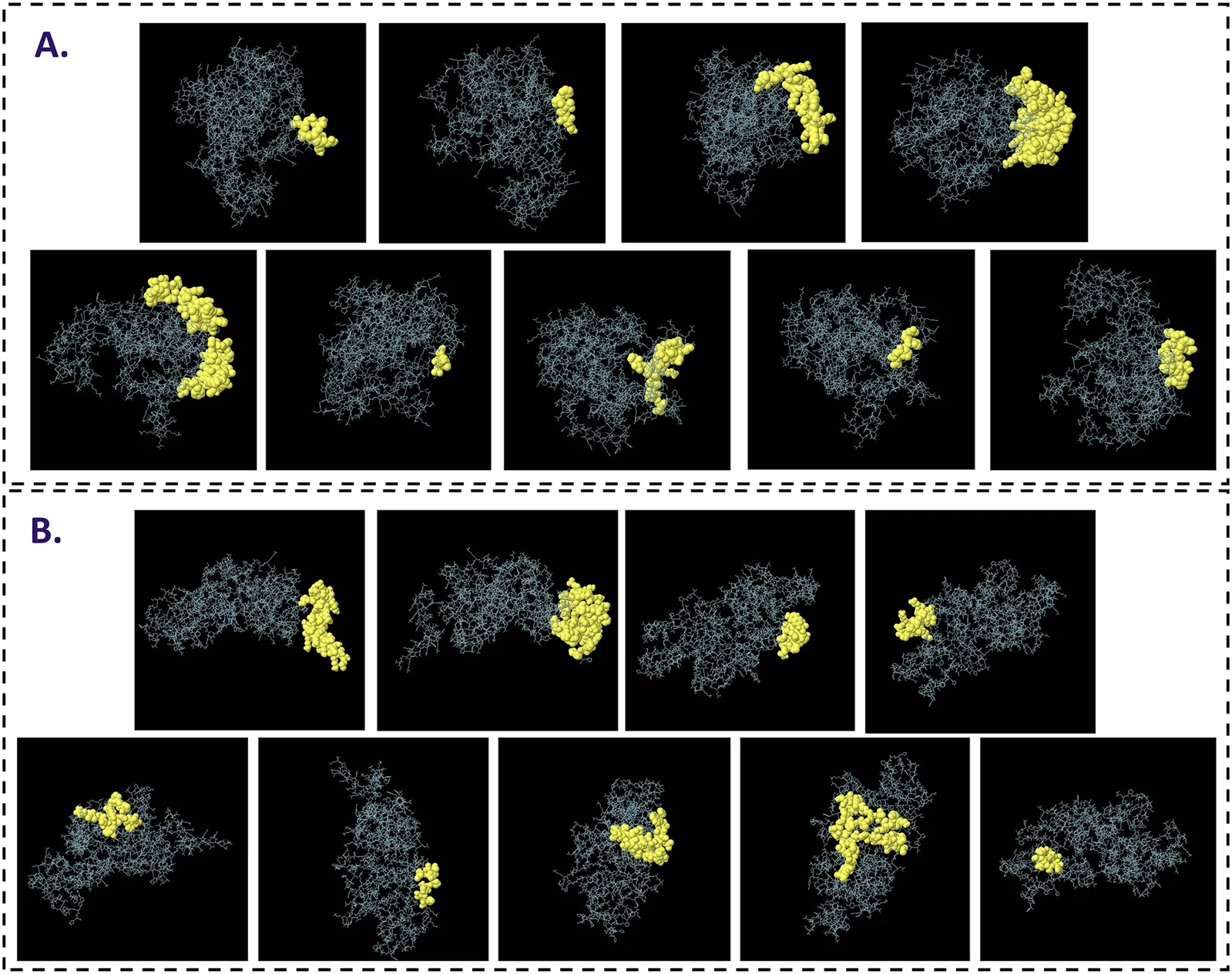
Prediction of structural B cell epitopes (yellow) by ElliPro server, for MB-saVaxI (A) and MB-saVaxII (B).

### 3.12. Molecular docking within the vaccine and TLR

Docking of MB-saVaxI with TLR2 and TLR4 was performed using ClusPro 2.0, and of the 30 results, two models were selected based on their central and lowest energies. The central and lowest energies of the model selected for MB-saVaxI-TLR2 were −821.5 and −938.3, respectively, whereas those for MB-saVaxI-TLR4 were −902.4 and −902.4, respectively. By contrast, MB-saVaxII was docked to TLR3 and TLR7. The central energy and the lowest energy of the selected models for MB-saVaxII-TLR3 were −1412.3 and −1485.4, whereas for MB-saVaxII-TLR7, the energy values were −1185.1 and −1543.6, respectively. The docked complexes were then visualized and evaluated with PyMOL and PDBsum. The PDBsum server predicted that the MB-saVaxI-TLR2 complex had 4 salt bridges, 14 hydrogen bonds, and 172 non-bonded interactions, while the MB-saVaxI-TLR4 complex had 10 salt bridges, 35 hydrogen bonds, and 312 non-bonded interactions (Figs 8 and 9). Meanwhile, the MB-saVaxII-TLR3 complex had 5 salt bridges, 10 hydrogen bonds, and 220 non-bonded interactions, whereas 5 salt bridges, 22 hydrogen bonds, and 262 non-bonded interactions were found in the MB-saVaxII-TLR7 complex.

**Fig 8:**
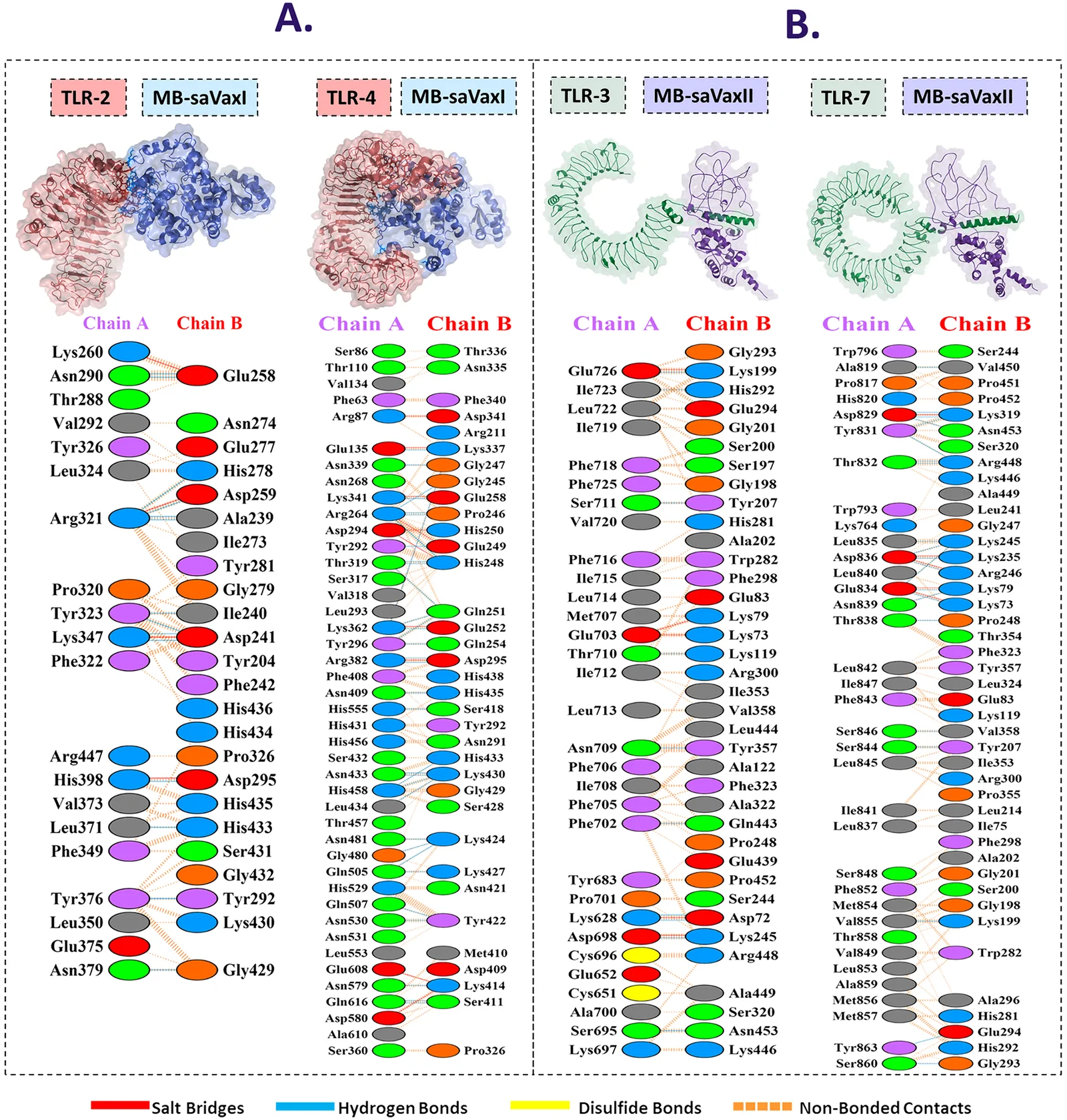
Analysis of the intermolecular interaction of MB-saVaxI-TLR2 and MB-saVaxI-TLR4 (A) and MB-saVaxII-TLR3 and MB-saVaxII-TLR7 (B) complexes.

**Fig 9:**
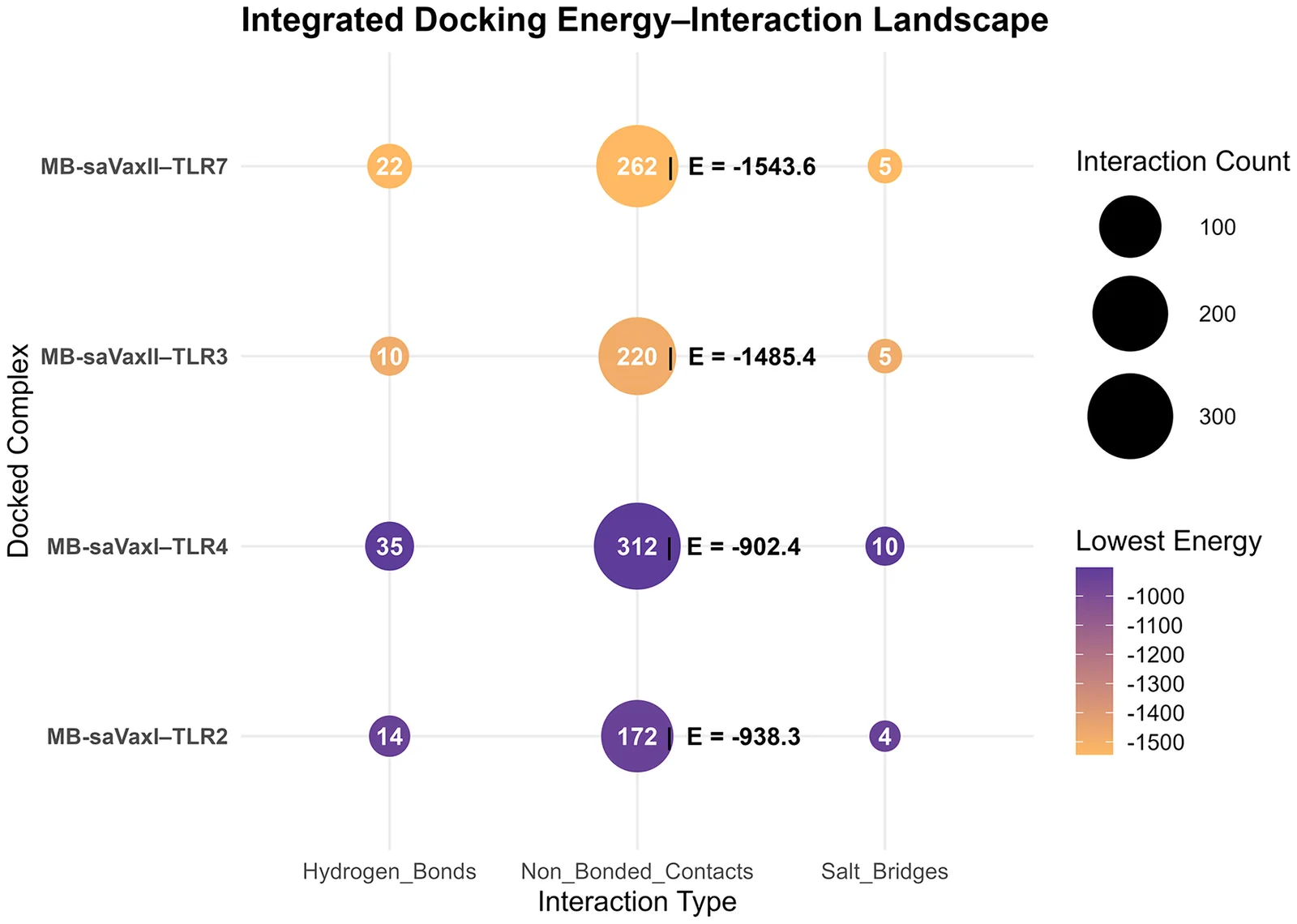
The bubble plot denotes the hydrogen bond, salt bridge, non-bonded contacts, and binding energy of the four vaccine complexes.

### 3.13. Molecular docking of T cell epitopes and HLA molecules

The HDOCK website was employed to evaluate the docking of selected T cell epitopes with HLA class I and II molecules. The website provided ten models for each epitope, and from them, one model was chosen. Finalized CD8 + epitopes of MB-saVaxI displayed docking scores of −170.49, −188.95, −175.03, −121.23, −183.41, and −192.42 for the epitopes FGPGIEGLY, LLLLSIAVL, AIALGVATA, QLSAIALGV, VAALLTGSY, and AQVLSKIAY, accordingly. Similarly, the predicted docking scores of the chosen CD4 + epitopes of MB-saVaxI TEERTFSLINRHAID, EERTFSLINRHAIDF, HEHQEIQAIAEDDEE, LNLSGINNLEHGLYP, ITPNYVGDLNLDDQF, and EGENAQAALTRLSRT were −223.09, −240.08, −170.81, −240.09, −177.14, and −183.23, respectively (Fig 9A). Consequently, for the CD8 + epitopes of MB-saVaxII DDLAAGLSW, HAQGIALHL, APFARVLNL, GLYPQLSAI, AYANPTLSA, DMFSVKEGM, AYKSGKSGA, and KSGKSGAFL, the docking scores are −202.2, −167.38, −178.68, −206.47, −213.50, −181.68, −179.59, and −124.83, correspondingly. Additionally, for the CD4 + epitopes of MB-saVaxII SGQKVADSPLEASKR, AKTCYNISVTDPSGK, EQGIVTYPNHWLTTG, HGEYAPFARVLNLSG, GITPNYVGDLNLDDQ, LNGFNNRQVVLAYAN, KIAYKSGKSGAFLDA, and SKIAYKSGKSGAFLD, the docking scores are −168.01, −217.89, −227.80, −255.57, −241.07, −205.65, −206.70, and −179.01 (Figs 9B and 10).

**Fig 10:**
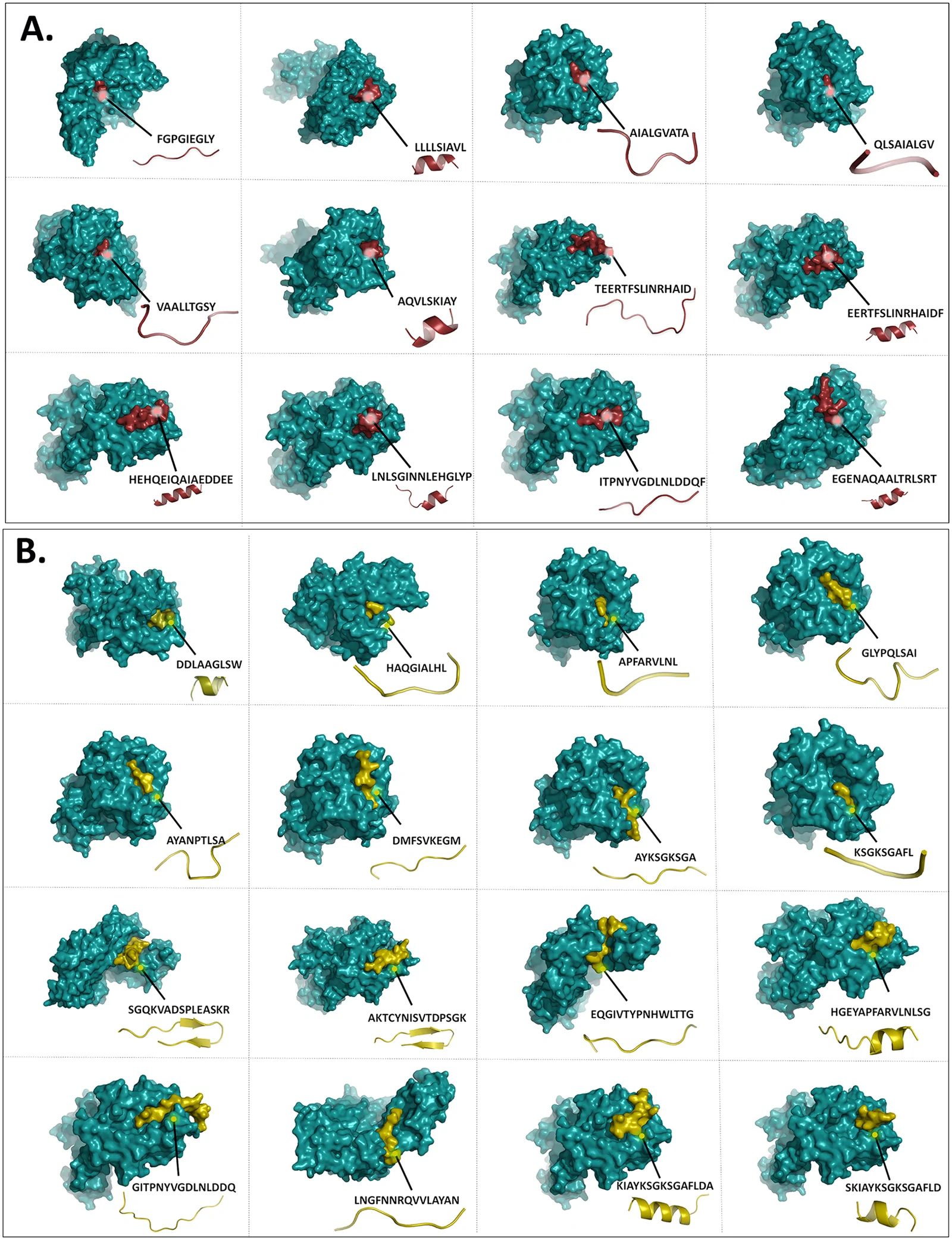
The docking between the HLA class I and class II molecule and the CD8 + epitope for MB-saVaxI (A), and MB-saVaxII (B). The molecules are shown in cyan, whereas the epitopes in MB-saVaxI are in cerise and those in MB-saVaxII are in olive.

### 3.14. Negative free energy calculation (MM-GBSA)

The HawkDock web server was used to estimate the free binding energy of MB-saVaxI-TLRs and MB-saVaxII-TLRs complexes. The results showed free binding energies of −65.44 and −101.69 kcal/mol for MB-saVaxI-TLR2 and MB-saVaxI-TLR4, respectively. Whereas, the free binding energy of MB-saVaxII-TLR3 and MB-saVaxII-TLR7 were −142.71 and −184.35 kcal/mol, respectively. Moreover, the GB, vdW, ELE, and SA of MB-saVaxI-TLR2 were predicted to be −535.79, −121.47, 606.54, and −14.72, respectively. Similarly, for MB-saVaxI-TLR4, the values were −2051.9, −208.55, 2186.64, and −27.88, accordingly. Conversely, the predicted values of GB, vdW, ELE, and SA were 539.28, −223.86, −427.5, and −30.63 for MB-saVaxII-TLR3, and 806.12, −255.5, −701.19, and −33.79 for MB-saVaxII-TLR7, respectively (Fig 11).

**Fig 11:**
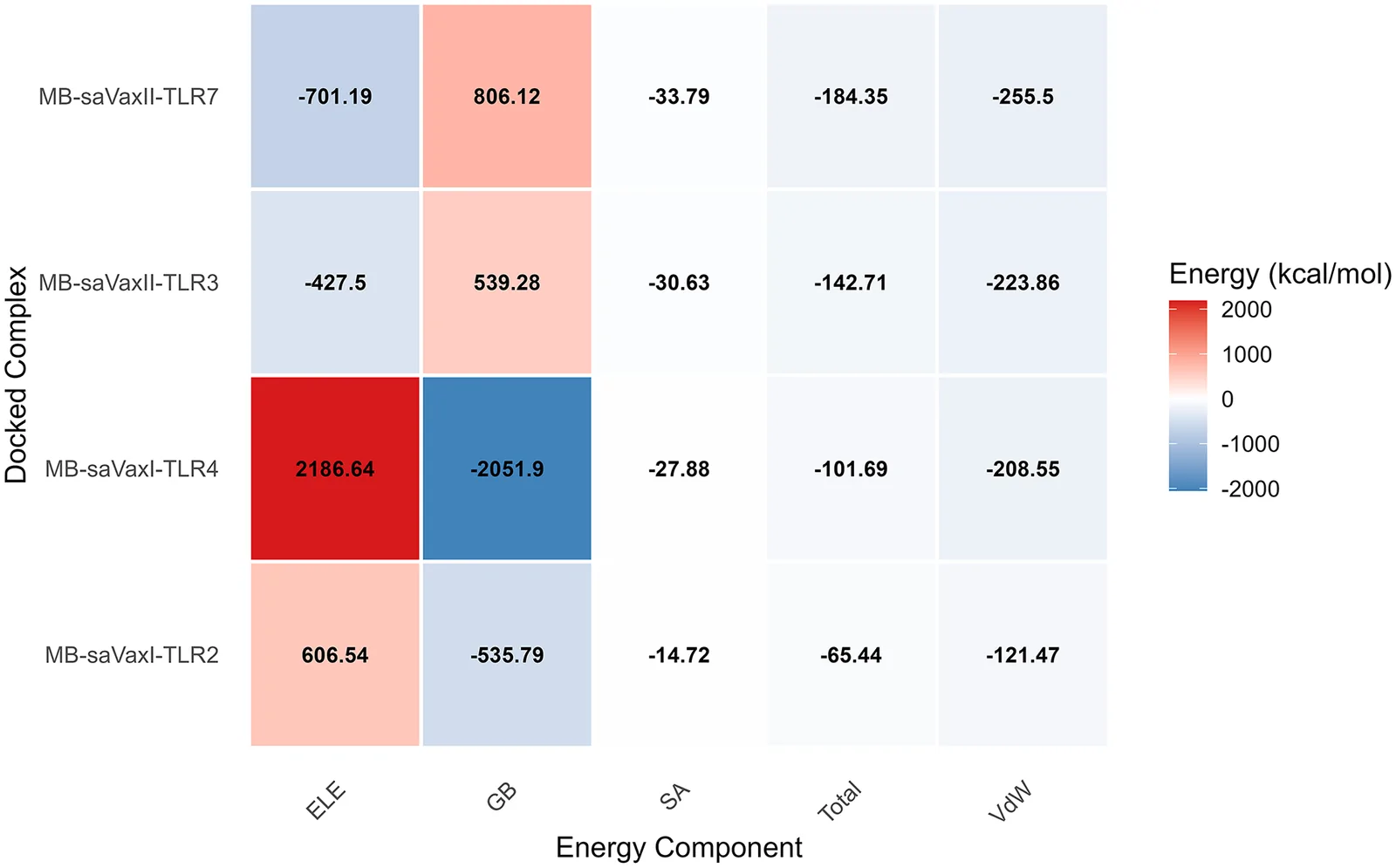
The heatmap represents the energy component of the docked complexes.

### 3.15. Molecular dynamics simulation (MDS)

MDS were performed to investigate the molecular binding stability, flexibility, and conformational compactness of the MB-saVaxI-TLR complexes, using root mean square deviation (RMSD), root mean square fluctuation (RMSF), hydrogen-bond analysis, and radius of gyration (rGyr) as metrics.

#### 3.15.1. RMSD analysis.

The RMSD quantified the motion of specific atoms within the complexes relative to the initial structure and conformational stability trends [78]. The average RMSD values of MB-saVaxI-TLR2 and MB-saVaxI-TLR4 complexes were 9.107 and 5.284 **Å**, respectively. During the initial 0–15 ns, the RMSD for the MB-saVaxI-TLR2 complex gradually increased, reaching a maximum of 8.575 **Å** (Fig 12A). Subsequently, the RMSD remained relatively stable, with minor fluctuations. The RMSD for MB-saVaxI-TLR4 remains almost constant throughout the simulation (Fig 12A). The lowest and highest RMSD values for the MB-saVaxI-TLR2 and MB-saVaxI-TLR4 complexes were 3.283, 10.716, and 2.771, 6.134 **Å**, respectively. This indicates that MB-saVaxI-TLR4 exhibited greater structural stability than MB-saVaxI-TLR2.

**Fig 12:**
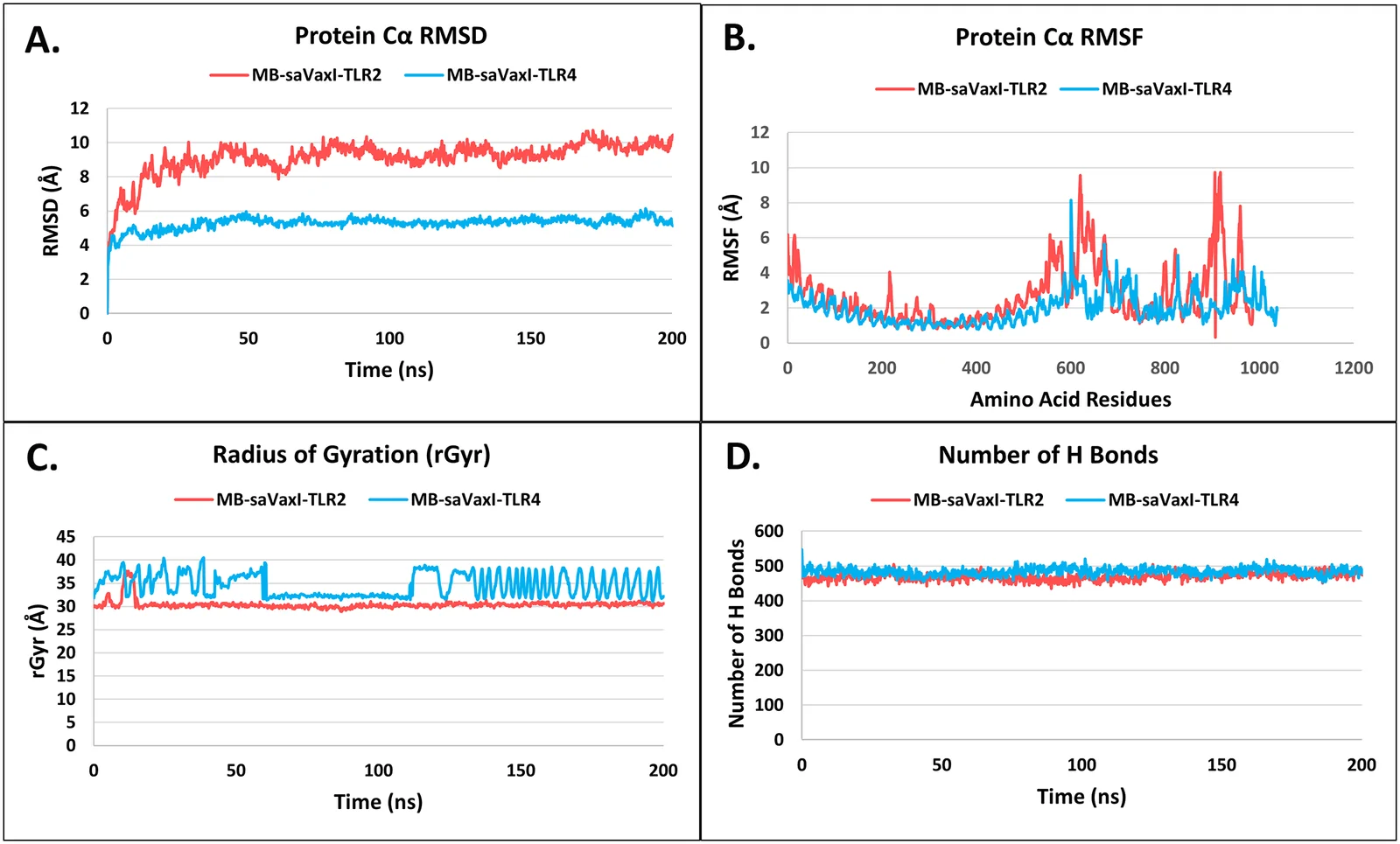
The MDS analysis of MB-saVaxI-TLR2 and MB-saVaxII-TLR4 complexes, visualizing RMSD, RMSF, rGyr, and hydrogen bond analysis.

#### 3.15.2. RMSF analysis.

RMSF was employed to quantify the native deviation of amino acid residues in a system, which illustrates the structural flexibility and stability of the complexes [77]. The average RMSF values for MB-saVaxI-TLR2 and MB-saVaxI-TLR4 were 2.664 and 1.966 **Å**, consequently. Moreover, graphical analysis of the RMSF showed that the MB-saVaxI-TLR2 complex exhibited the lowest and highest RMSF values of 0.331 and 9.732 **Å**, accordingly, at the ASN_359 and LEU_369 residue regions. Conversely, the MB-saVaxI-TLR4 complex had the lowest and highest RMSF values of 0.744 and 8.135 **Å**, respectively, at the LEU_290 and MET_1 residue region (Fig 12B). Despite modest changes, both MB-saVaxI-TLR2 and MB-saVaxI-TLR4 complexes demonstrated similar overall structural stability.

#### 3.15.3. rGyr analysis.

The rGyr demonstrated the three-dimensional organization of the atoms within the complexes in relation to their main axis, indicating the stiffness and structural integrity of the macromolecular complexes [79,87]. An average rGyr of 30.401 **Å** and 34.526 **Å** was recorded for MB-saVaxI-TLR2 and MB-saVaxI-TLR4 complexes, respectively. Furthermore, the MB-saVaxI-TLR2 complex was stable, with no notable conformational alterations, indicating enhanced structural integrity and denser packing (Fig 12C). Meanwhile, the MB-saVaxI-TLR4 experienced noticeable fluctuations between 1–60 ns and 110–200 ns.

#### 3.15.4. H-bond analysis.

H-bond analysis was performed to quantify the number of H-bonds formed between the vaccine and TLRs; a higher number of H-bonds indicated greater structural stability [88]. For MB-saVaxI-TLR2 and MB-saVaxI-TLR4, the average quantity of H-bonds formed was 469.549 and 484.056, respectively. Moreover, the highest H-bond counts were 510 and 547 for the MB-saVaxI-TLR2 and TLR4 complexes, respectively, whereas the lowest counts were 435 and 453 for the MB-saVaxI-TLR2 and TLR4 complexes, respectively (Fig 12D). Despite the MB-saVaxI-TLR4 complex showing a marginally higher number of H-bonds, both complexes exhibited comparable H-bond stability throughout the simulation period.

### 3.16. Codon optimization and computational cloning

JCat provided a 1314-nucleotide MB-saVaxI and 1416-nucleotide MB-saVaxII DNA sequences following codon optimization in *E. coli* K12. The website reported GC content and CAI values of 51.44% and 0.98 for MB-saVaxI and 52.82% and 0.99 for MB-saVaxII, respectively, indicating effective protein expression in *E. coli.* Subsequently, the optimized sequences were inserted into the pcTMDNA3.1(+) vector to construct recombinant plasmid DNA using SnapGene software (Fig 13).

**Fig 13:**
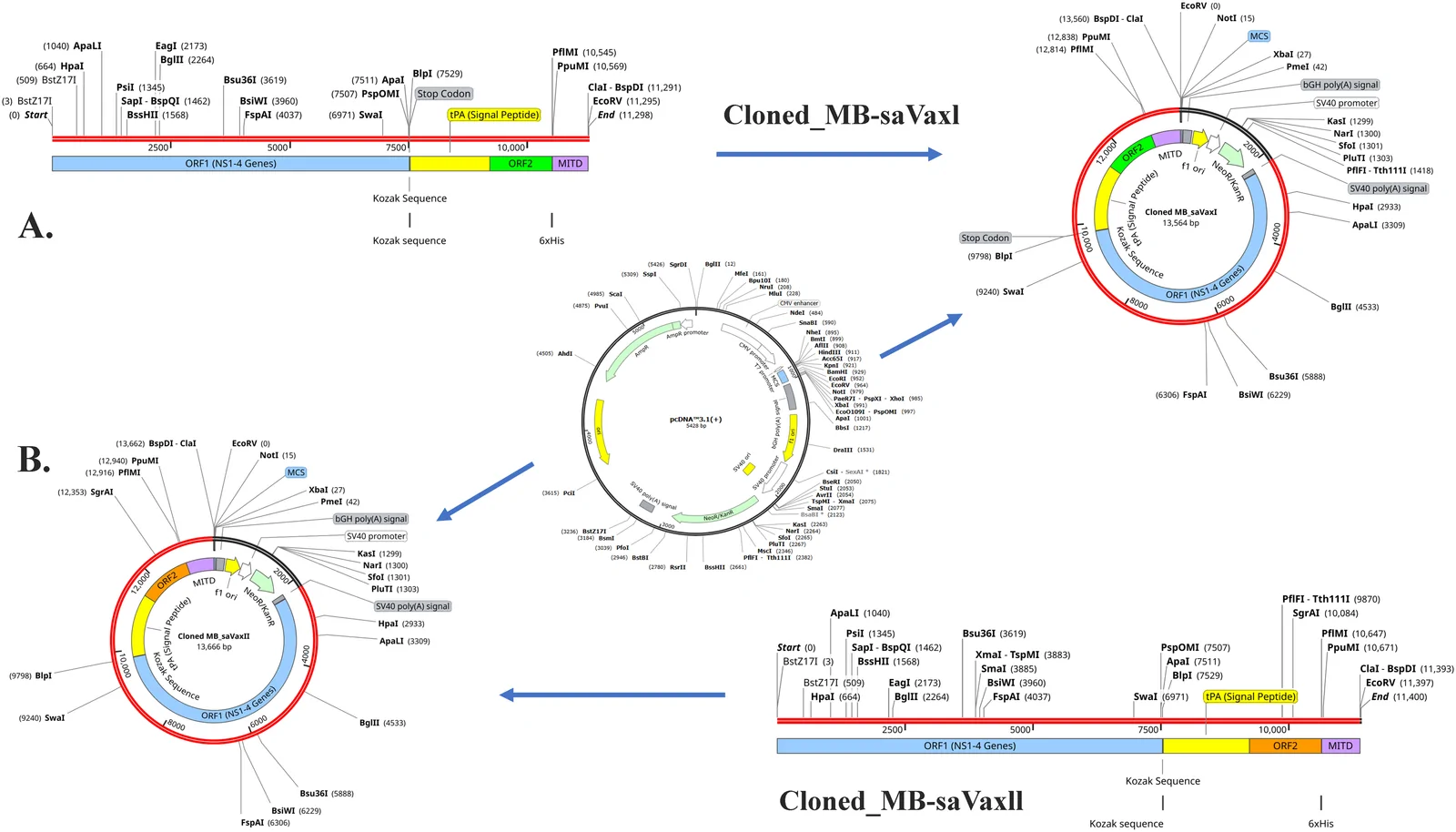
*In-silico* cloning of the optimized vaccine between the BstZ17I and EcoRV restriction sites of the mammalian expression vector pc^TM^DNA3.1(+).

### 3.17. Immunological response simulation

Computer-aided immunological response simulation was carried out by administering three doses of both vaccines to assess the ability of the MB-saVaxI and MB-saVaxII to induce a robust adaptive immune response. After the immunization, a notable increase in TC cell, TH cell, and TR (regulatory) cell concentration values was observed by both vaccines, as well as an elevation in active and memory TH cells on day 70. Similarly, high levels of active TR and TC were seen one year following immunization. Moreover, there was a significant rise in memory B cells, and prolonged immunity is indicated by the total B cells remaining active. Furthermore, the IgM + IgG titre reached the highest level at day 60 and started decreasing from day 100 and persisted for 1 year at a constant phase. However, the antigen concentration reached a sharp peak at days 1, 25, and 50, although it reduced remarkably after day 50. Additionally, the cytokine response was also induced, but only for 1 year after immunization, and a persistent year-long immunity is also indicated by the high levels and sustained activity of natural killer (NK) cells and dendritic cells (DC) (Fig 14).

**Fig 14:**
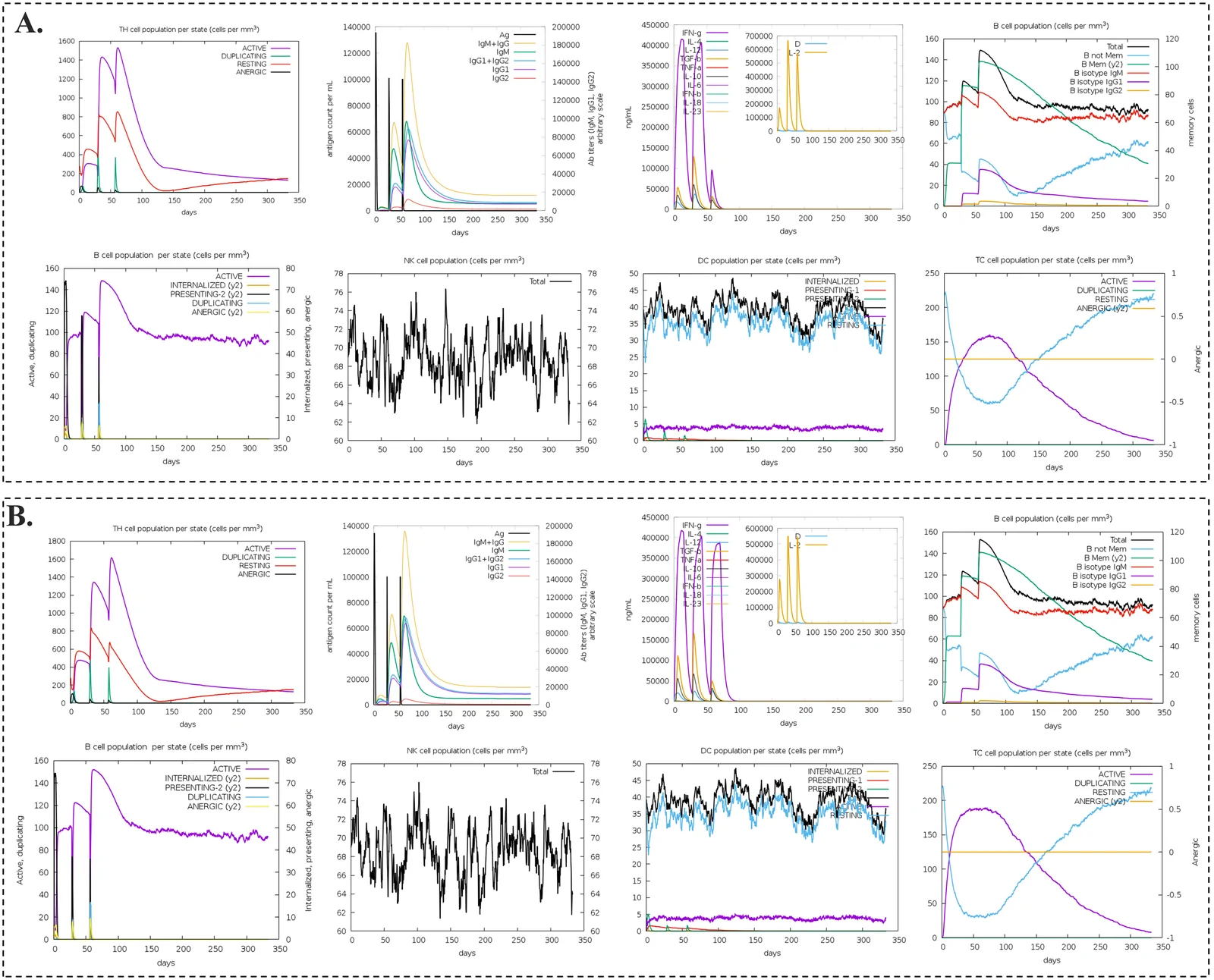
Immune simulation evaluation of the MB-saVaxI and MB-saVaxII employing the C-immsim server (A and B, respectively).

### 3.18. Modelling of the 2˚ structure of the mRNA vaccine

According to the RNAfold website, the thermodynamic ensemble free energy and the MFE score of the centroid and optimal structures of MB-saVaxI mRNA were −429.41, −330.90, and −407.40 kcal/mol, respectively. Conversely, the MFE score of the centroid and optimal structures of MB-saVaxII mRNA were −276.80 and −461.50 kcal/mol, respectively. Moreover, the thermodynamic ensemble free energy of MB-saVaxII mRNA was −485.64 kcal/mol. The website also predicted an MFE structure frequency of 0.00% in the ensemble (Fig 15).

**Fig 15:**
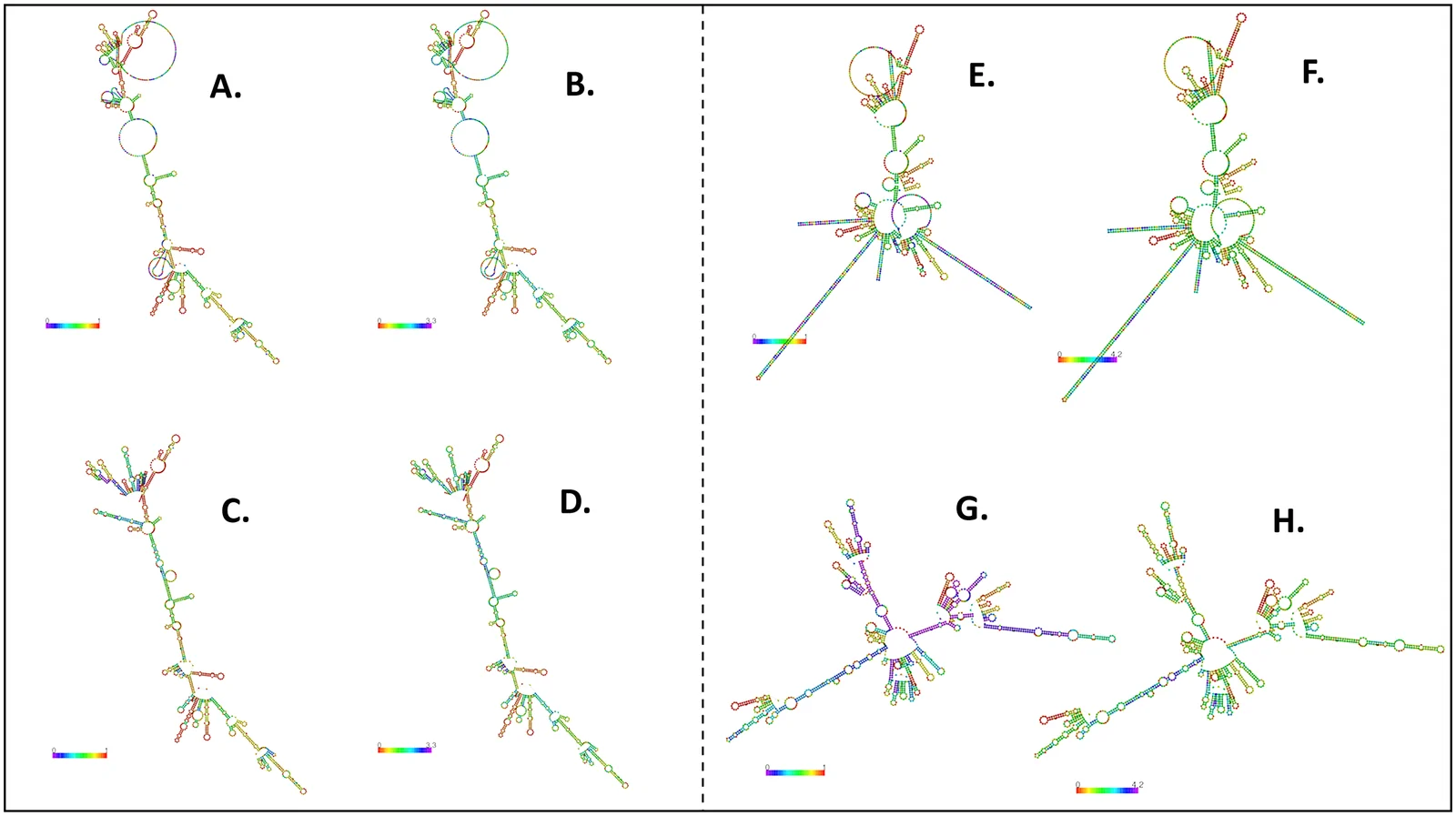
The predicted mRNA structures for MB-saVaxI and MB-saVaxII. The centroid mRNA secondary structures are colored according to base-pair probabilities (A and B) for MB-saVaxI and (E and F) for MB-saVaxII and positional entropy (C and D) for MB-saVaxI and (G and H) for MB-saVaxII. The red signifies nucleotides with a high probability of base pairing, whereas colors ranging from yellow to violet indicate progressively lower pairing probabilities, and green indicates moderate probabilities. In the positional entropy, the red color indicates low entropy, associated with highly stable base pairs. The green color denotes intermediate entropy levels, while colours ranging from yellow to violet represent increasing entropy and structural flexibility.

## 4. Discussion

MARV is a highly virulent pathogenic virus that typically impacts liver tissues, causing dysfunctional immune systems and serious systemic infection [89]. Since its initial identification in 1967, there have been severe and sporadic outbreaks, with a documented mortality rate of up to 88% [90]. Moreover, preventing MVD outbreaks has become more challenging in recent times as numerous human reports have emerged around the world. Due to the absence of an FDA-authorized vaccine and the possibility for aerosol transmission, MARV research is limited to extreme-containment Biosafety Level 4 (BSL-4) laboratory environments [90]. Nevertheless, several experimental vaccines are under development. The most clinically advanced candidate, the Sabin Vaccine Institute’s chimpanzee adenovirus type 3-vectored vaccine (cAd3-MARV) encoding the MARV glycoprotein, was safe and immunogenic in a first-in-human phase 1 trial in the United States (NCT03475056), with 95% of participants developing glycoprotein-specific antibodies [91]. Nevertheless, as a replication-incompetent viral vector, cAd3-MARV may be difficult to administer repeatedly because pre-existing anti-vector immunity can reduce its effectiveness, particularly in adenovirus-exposed populations. In addition, its clinical efficacy has not yet been demonstrated. Similarly, live-attenuated rVSV∆G-MARV-GP candidates (PHV01, NCT06265012; IAVI’s rVSV∆G-MARV-GP, NCT07425821) have entered phase 1 testing [92]. However, these replication-competent live viral vaccines may cause reactogenicity and viremia and require strict cold-chain storage. In addition, human correlates of protection remain undefined, and their clinical efficacy has not yet been established [93,94]. Additionally, conventional vaccine development may need years to identify the most effective antigens and refine the vaccine composition. Conversely, reverse vaccinology-based saRNA vaccines offer several advantages over conventional approaches. As of today, several multi-epitope vaccines targeting MARV have been designed using bioinformatics tools, but no saRNA vaccines have been specifically assembled [95–98]. Compared with traditional multi-epitope vaccines, the saRNA vaccine generates more antigen over longer periods as it replicates within the cell and, in preclinical experiments, has been observed to produce long-lasting protection with lower doses [99]. Therefore, this study focuses on immunoinformatics methods for the construction of saRNA vaccines [100]. In this study, the saRNA vaccines were designed using antigenic, antitoxic, and non-allergenic epitopes from the GP, NP, VP40, and VP35 proteins of MARV. Subsequently, the conservation assessment of the vaccines revealed that GP (94.74%), NP (100%), VP40 (100%), and VP35 (100%) displayed the highest concentrations of conserved residues, suggesting that these epitopes could withstand the rapid mutation rates of various viral variants. Consequently, the bL12 adjuvant was incorporated to enhance the overall immunopotency of MB-saVaxI and MB-saVaxII [101]. The designed MB-saVaxI and MB-saVaxII exhibited worldwide population coverage of 98.98% and 96.21%, respectively, for the combination of CD8+ and CD4 + epitopes, indicating that these vaccines are likely to induce immunoreactivity across diverse populations [102]. Meanwhile, Mohamed et al. (2022) and Bishal et al. (2023) did not discuss geographical population coverage, whereas Saad et al. (2021) and Fouad et al. (2024) reported population coverage of 97.92% and 93.94%, respectively [95–98]. Afterwards, the constructed vaccines were calculated to have instability indices of 32.01 and 24.15 for MB-saVaxI and MB-saVaxII, respectively, indicating that the proteins are stable. While the aforementioned Bishal et al. (2023) did not mention the instability index, Mohamed et al. (2022), Saad et al. (2021), and Fouad et al. (2024) reported instability scores of 38.9, 33.8, and 38.57, respectively [95–98]. Moreover, the aliphatic index of the MB-saVaxI and MB-saVaxII was predicted to be 82.40 and 74.22, respectively, suggesting the thermostability of the sequences at normal body temperature [103]. Compared with this, Saad et al. (2021), Mohamed et al. (2022), and Fouad et al. (2024) reported aliphatic indices of 77.08, 73.3, and 73.30, respectively [95–98]. Since protein folding defines the final structure and biological activity of proteins, knowledge of how proteins fold from primary to tertiary structure is vital for successful vaccine development [104]. Throughout the study, favourable results were observed for MB-saVaxI and MB-saVaxII in the analysed protein 2˚ and 3˚ structures. The RamPlot assessment also revealed that the majority of the vaccine residues were concentrated in the favored domains (93.349% for MB-saVaxI and 92.202% for MB-saVaxII). In contrast, Bishal et al. (2023), Saad et al. (2021), and Mohamed et al. (2022) utilized 89.5%, 93.2%, and 93.3% residues, respectively, in the most favored regions [95–98]. To assess the binding interactions between the vaccines and TLRs on immune cells, molecular docking was conducted using the human TLR2 and TLR4 receptors with MB-saVaxI, and the TLR3 and TLR7 receptors with MB-saVaxII [105]. Based on this analysis, MB-saVaxI displayed an immense affinity to TLR2 (lowest energy score of −938.3) and TLR4 (lowest energy score of −902.4) receptors, where MB-saVaxII exhibited a binding affinity toward TLR3 (lowest energy score of −1485.4) and TLR7 (lowest energy score of −1543.6) receptors. On the contrary, the Saad et al. (2021) and Mohamed et al. (2022) vaccines predicted the lowest values of −1034.9 and −1458, respectively, highlighting the docking accuracy of the present designed vaccines [95–98]. To determine conformational changes and efficient immune recognition, MD simulations were conducted on the vaccine complexes in association with their TLRs. Bishal et al. (2023) conducted the MDS for 50 ns; in addition, Saad et al. (2021), Mohamed et al. (2022), and Fouad et al. (2024) performed simulations for 100 ns each. According to this research, the MDS was applied for 200 ns to MB-saVaxI-TLR2 and MB-saVaxI-TLR4, yielding a more comprehensive understanding of the dynamic system and improved statistical convergence [106]. Consequently, the structural stability and flexibility of individual residues in the constructed complexes were assessed using RMSD and RMSF relative to the primary reference structure, respectively. Nonetheless, RMSD and RMSF scores below 1 nm (10 Å) indicate close structural resemblance between the two overlaid structures [106]. However, the average RMSD scores were 9.107 and 5.284, respectively, demonstrating the structural integrity of the vaccine complexes. Furthermore, the mean RMSF scores were 2.66 and 1.66, both below 10 Å, indicating stable conformation throughout the simulations [107]. Notably, the vaccine-receptor complexes exhibited structural expansion and intermolecular interactions, as indicated by the Radius of gyration and average H-bond scores between the constructed vaccines and the TLRs [85,108]. Although the MB-saVaxI-TLR4 complex exhibits continual fluctuations in radius of gyration during the simulation, this behaviour likely indicates inherent conformational flexibility rather than structural instability [108]. Additionally, to enhance expression of these recombinant vaccines, codon optimization was performed in *E. coli*, particularly the K12 strain. Fouad et al. (2024) reported a CAI score of 1.0 and a GC content of 50.73% [95–98]. Meanwhile, MB-saVaxI and MB-saVaxII had a CAI score of 0.981 and 0.996, respectively, along with GC content of 51.44% and 52.82%, respectively, indicating a high likelihood of efficient expression, optimal cloning probability, and genetic stability within the acceptable ranges for GC content (30–70%) and CAI (~1.0) [101]. After a three-dose course, both vaccines elicited an effective and durable immune response, with a stronger B cell population and prolonged stimulation of TH and TC cells, suggesting the potential for sustained immune function and enhanced immunity against MARV [109]. Furthermore, the minimum free energy (MFE) of the vaccine depends on sequence and length, with MFE values ranging from −50 to −250 kcal/mol [110]. Additionally, the constructed MB-saVaxI displayed probable structural stability due to MFE scores of −407.40 kcal/mol (optimal) and −330.90 kcal/mol (centroid). Similarly, MB-saVaxII indicates structural integrity, with MFE scores of −461.50 kcal/mol (optimal) and −276.80 kcal/mol (centroid), as higher negative energies often indicate enhanced stability. Moreover, the current study integrates a modified computational framework that encompasses post-synthetic protein modification analysis, disulfide engineering, structural B cell epitope prediction, and docking of T-cell epitopes with HLA molecules. In addition, this study presents a more extensive and comprehensive assessment of the proposed Vaccine design than earlier studies.

In light of this, the proposed vaccine structure will probably maintain stability throughout the processes of entry into the host, transcription, and expression. Although the findings were favorable, this study focused exclusively on the immunoinformatics process. This process has limitations, including inaccurate structural predictions and a lack of chronic immunity predictions. Furthermore, all findings were generated through computational predictions, and no experimental validation was performed. Therefore, laboratory and animal studies are needed to confirm the immunogenicity and safety of MB-saVaxI and MB-saVaxII. Likewise, epitope prediction depends on the algorithms and databases used, which may not fully represent all HLA types in the global population. To address this bias, only epitopes with high conservancy were retained. Moreover, molecular dynamics simulations were performed over a limited timescale and may not capture long-term structural behavior. Overall, the stability and expression of the designed saRNA constructs were assessed only *in silico*. These limitations were partly addressed through the use of validated tools and strict selection criteria. However, experimental studies are required before clinical translation. Further research, including preclinical and clinical studies, will be essential to precisely verify the immunogenicity, efficacy, and stability of these vaccines.

## 5. Conclusion

Next-generation immunoinformatics technology has enabled the prevention and control of highly pathogenic, life-threatening diseases by using computational tools to design vaccines against them. This study intends to formulate an saRNA vaccine candidate against MVD by targeting proteins involved in pathogenesis. The study outcomes suggest that the vaccine, designed using antigenic epitopes, has the potential to induce a strong immune response without causing allergic reactions or toxicity. Additionally, the selected epitopes showed high conservation and substantial population coverage across diverse regions worldwide. Molecular docking scores indicated high-affinity interactions with TLR2, TLR3, TLR4, and TLR7 receptors, which were further validated by MM-GBSA analysis. The vaccine also showed strong cloning potential when expressed in a bacterial vector, and immunological response simulations suggested it could efficiently stimulate both cell-mediated and antibody-mediated immune responses. Moreover, mRNA stability was assessed using thermodynamic free energy and MFE values. Overall, these findings demonstrate that the bioinformatically designed vaccine was a strong, viable candidate for countering MVD. However, these findings must be validated through laboratory and animal testing to assess vaccine efficacy and safety.

## Supporting information

S1 File Conservancy, coverage, disulfide, and conformational B-lymphocyte epitopes. (DOCX)

S2 File Protein RMSD. (DOCX)

S3 File Protein RMSF. (DOCX)

S4 File Radius of Gyration. (DOCX)

S5 File Hydrogen Bond. (DOCX)

S6 File saRNA Sequences. (DOCX)
