# Supplementary material for: Immunoinformatics and reverse vaccinology design of self-amplifying RNA vaccine against marburg virus with structural dynamics and immune simulation analysis
Source: PLoS One. 2026 Oct 23;21(10):e0361136. doi: 10.1371/journal.pone.0361136 (PMC13693908; doi:10.1371/journal.pone.0361136)
Supplement: Supplementary file 1 [file pone.0361136.s007.pdf]

Overall, this study focuses on the computational design and evaluation of two self-amplifying RNA (saRNA) vaccine candidates against Marburg virus disease (MVD). Initially, key Marburg virus proteins, including glycoprotein (GP), nucleoprotein (NP), VP40, and VP35, were selected, and their sequences were retrieved from the NCBI database. Subsequently, suitable epitopes for CD8+ and CD4+ T-cell responses, as well as humoral B-cell responses, were identified using bioinformatics tools and evaluated based on antigenicity, allergenicity, toxicity, sequence conservation, and population coverage. The selected epitopes were incorporated into two saRNA vaccine constructs, designated MB-saVaxI and MB-saVaxII, along with adjuvant components and other elements intended to enhance antigen expression and presentation. The physicochemical and structural properties of the vaccine constructs, including secondary and tertiary protein structures and B-cell epitopes, were subsequently assessed. Their potential interactions with TLR and HLA receptors, binding affinities, and complex stability were evaluated using molecular docking, MM-GBSA calculations, and molecular dynamics simulations. In addition, codon optimization, in silico cloning, immune response simulation, and prediction of the mRNA secondary structure were performed. Overall, the computational findings indicated that both constructs exhibited favorable characteristics in terms of epitope selection, population coverage, structural properties, and predicted immune responses, suggesting that they may serve as potential candidates for future experimental investigation against Marburg virus disease. However, their actual efficacy, safety, stability, and immunogenicity remain to be confirmed through in vitro and in vivo studies, followed by appropriate animal and clinical investigations.

Abstract:

It is stated that a pI < 7 indicates the vaccine is basic, whereas a pI of less than 7 indicates an acidic nature.

Two saRNA vaccines were cloned in *Escherichia coli*, but it was not specified exactly what was cloned.

The results regarding the immune response have been stated unequivocally.

Additionally, it has been repeated several times.

Introduction:

The NP protein forms the ribonucleoprotein complex required for transcription and translation; however, the primary role of this complex is in the replication and transcription of the viral genome, whereas the translation of viral proteins is carried out by host cell ribosomes.

They have explained the functions of GP, NP, VP40, and VP35, but have not specified exactly why these four proteins were selected for vaccine epitope selection.

check for grammatical correctness.

Methods and materials:

Regarding CD4+, it is only stated that 15-mer epitopes were selected based on binding strength; no specific cutoff value is provided.

It is unclear how many MARV sequences and of which strains were included in the analysis, or from which database or collection they were obtained.

The authors optimized the DNA sequence for *E. coli* K-12; however, the final product is an saRNA vaccine intended for expression in eukaryotic cells. If E. coli is used solely for plasmid propagation, the codon optimization strategy should be justified based on the final expression system rather than the bacterial host alone. This section needs to clarify the intended purpose of the DNA construct and the specific cellular system in which the RNA is ultimately meant to be expressed.

Check for grammatical correctness

Some sentences are incomplete.

Results:

Inconsistency regarding the conservation criterion The Methods section states: The analysis used a threshold of ≥100% sequence identity to ensure only fully conserved epitopes were chosen. However, in the Results section, conservation levels significantly lower than 100% are reported for some epitopes for instance, 21.05%, 42.31%, 50%, and 75%.

Merely having a CAI of 0.98 or 0.99 and an appropriate GC content does not guarantee effective protein expression. These parameters only indicate the sequence's relative suitability for translation within the host.

Docking the vaccine with TLR2, TLR3, TLR4, and TLR7 and reporting hydrogen bonds, salt bridges, and docking scores canot demonstrate that the vaccine actually activates TLR receptors or stimulates an immune response. Therefore, terms such as "increased immune response" should be used with caution and framed in terms of predicted or putative interactions.

A sufficient methodological explanation regarding why these TLRs were selected for each construct has not been provided. Furthermore, the nature of the vaccine, the type of RNA, and the mechanism by which it accesses the various TLRs must be explained in a manner consistent with the biology of the receptors.

Some words and sentences are incorrect in terms of grammatical structure.

Discussion:

RMSD and RMSF are distinct metrics, and a fixed threshold of 1 nm cannot be established as a universal criterion of stability for both.

The claim that high conservation indicates epitopes remain resistant to mutation is incorrect.

The logical flow of the Discussion is weak.

Conclusion:

The study results have been interpreted as overly definitive in several sections, whereas the study is entirely computational in silico.

Overall, this study focuses on the computational design and evaluation of two self-amplifying RNA (saRNA) vaccine candidates against Marburg virus disease (MVD). Initially, key Marburg virus proteins, including glycoprotein (GP), nucleoprotein (NP), VP40, and VP35, were selected, and their sequences were retrieved from the NCBI database. Subsequently, suitable epitopes for CD8+ and CD4+ T-cell responses, as well as humoral B-cell responses, were identified using bioinformatics tools and evaluated based on antigenicity, allergenicity, toxicity, sequence conservation, and population coverage. The selected epitopes were incorporated into two saRNA vaccine constructs, designated MB-saVaxI and MB-saVaxII, along with adjuvant components and other elements intended to enhance antigen expression and presentation. The physicochemical and structural properties of the vaccine constructs, including secondary and tertiary protein structures and B-cell epitopes, were subsequently assessed. Their potential interactions with TLR and HLA receptors, binding affinities, and complex stability were evaluated using molecular docking, MM-GBSA calculations, and molecular dynamics simulations. In addition, codon optimization, in silico cloning, immune response simulation, and prediction of the mRNA secondary structure were performed. Overall, the computational findings indicated that both constructs exhibited favorable characteristics in terms of epitope selection, population coverage, structural properties, and predicted immune responses, suggesting that they may serve as potential candidates for future experimental investigation against Marburg virus disease. However, their actual efficacy, safety, stability, and immunogenicity remain to be confirmed through in vitro and in vivo studies, followed by appropriate animal and clinical investigations.
