# Supplementary material for: Immunoinformatics and reverse vaccinology design of self-amplifying RNA vaccine against marburg virus with structural dynamics and immune simulation analysis
Source: PLoS One. 2026 Oct 23;21(10):e0361136. doi: 10.1371/journal.pone.0361136 (PMC13693908; doi:10.1371/journal.pone.0361136)
Supplement: Supplementary file 2 [file pone.0361136.s001.docx]

**Supplementary Table 1.** The conservation percentage of the predicted epitopes among various Marburg virus (MB-saVaxI) strains.

| **Protein** | **Epitope** | **Percent of protein sequence matches at identity <= 100%** |
| --- | --- | --- |
| **GP** | FGPGIEGLY | 92.11% |
|  | LLLLSIAVL | 94.74% |
|  | TEERTFSLINRHAID | 94.74% |
|  | EERTFSLINRHAIDF | 94.74% |
|  | IDFDPVPNTKTIFDESSSSGASAEEDQHASPN | 21.05% |
|  | QKVADSPLEAS | 92.11% |
|  | TGVPPKNVEYTEGEEAK | 89.47% |
| **NP** | AIALGVATA | 100.00% |
|  | QLSAIALGV | 100.00% |
|  | HEHQEIQAIAEDDEE | 80.77% |
|  | LNLSGINNLEHGLYP | 100.00% |
|  | YQNPVSAKELQADWPDMSFD | 50.00% |
| **VP40** | VAALLTGSY | 94.44% |
|  | ITPNYVGDLNLDDQF | 88.89% |
|  | MASSSNYN | 88.89% |
| **VP35** | AQVLSKIAY | 87.50% |
|  | EGENAQAALTRLSRT | 100.00% |
|  | YKSGKSG | 100.00% |

**Supplementary Table 2.** The conservation percentage of the predicted epitopes among various Marburg virus strains (MB-saVaxII).

| **Protein** | **Epitope** | **Percent of protein sequence matches at identity <= 100%** |
| --- | --- | --- |
| **GP** | DDLAAGLSW | 94.74% |
|  | HAQGIALHL | 94.74% |
|  | SGQKVADSPLEASKR | 92.11% |
|  | AKTCYNISVTDPSGK | 94.74% |
|  | IDFDPVPNTKTIFDESSSSGASAEEDQHASPN | 21.05% |
| **NP** | APFARVLNL | 100.00% |
|  | GLYPQLSAI | 100.00% |
|  | EQGIVTYPNHWLTTG | 46.15% |
|  | HGEYAPFARVLNLSG | 100% |
|  | TKPTAPHVR | 42.31% |
|  | VGEQYQQL | 88.46% |
| **VP40** | AYANPTLSA | 100.00% |
|  | DMFSVKEGM | 100.00% |
|  | GITPNYVGDLNLDDQ | 94.44% |
|  | LNGFNNRQVVLAYAN | 100.00% |
|  | PENFPLN | 100.00% |
| **VP35** | AYKSGKSGA | 100.00% |
|  | KSGKSGAFL | 100.00% |
|  | KIAYKSGKSGAFLDA | 95.83% |
|  | SKIAYKSGKSGAFLD | 95.83% |
|  | LRAVPPNPTID | 91.67% |

**Supplementary Table 3.** The percentage of population coverage of the chosen epitopes across several geographical locations. (MB-saVaxI)

| **Region** | **Combined** | **CD8+ epitope** | **CD4+ epitope** |
| --- | --- | --- | --- |
| **World** | 98.98% | 79.67% | 95.00% |
| **East Asia** | 99.04% | 78.80% | 95.46% |
| [**Northeast Asia**](https://tools.iedb.org/population/result/#Northeast%20Asia) | 98.65% | 55.62% | 96.95% |
| [**South Asia**](https://tools.iedb.org/population/result/#South%20Asia) | 95.98% | 56.35% | 90.79% |
| [**Southeast Asia**](https://tools.iedb.org/population/result/#Southeast%20Asia) | 96.53% | 47.33% | 93.42% |
| [**Southwest Asia**](https://tools.iedb.org/population/result/#Southwest%20Asia) | 95.93% | 67.09% | 87.63% |
| [**Europe**](https://tools.iedb.org/population/result/#Europe) | 99.19% | 88.92% | 92.71% |
| [**East Africa**](https://tools.iedb.org/population/result/#East%20Africa) | 99.37% | 70.07% | 97.89% |
| [**West Africa**](https://tools.iedb.org/population/result/#West%20Africa) | 99.50% | 73.11% | 98.14% |
| [**Central Africa**](https://tools.iedb.org/population/result/#Central%20Africa) | 98.88% | 65.12% | 96.80% |
| [**North Africa**](https://tools.iedb.org/population/result/#North%20Africa) | 97.92% | 72.25% | 92.50% |
| [**South Africa**](https://tools.iedb.org/population/result/#South%20Africa) | 64.63% | 62.41% | 5.91% |
| [**West Indies**](https://tools.iedb.org/population/result/#West%20Indies) | 98.06% | 83.22% | 88.44% |
| [**North America**](https://tools.iedb.org/population/result/#North%20America) | 99.50% | 83.50% | 96.99% |
| [**Central America**](https://tools.iedb.org/population/result/#Central%20America) | 99.75% | 4.33% | 99.74% |
| [**South America**](https://tools.iedb.org/population/result/#South%20America) | 99.36% | 51.29% | 98.69% |
| [**Oceania**](https://tools.iedb.org/population/result/#Oceania) | 99.21% | 37.88% | 98.73% |

**Supplementary Table 4.** The percentage of population coverage of the chosen epitopes across several geographical locations. (MB-saVaxII)

| **Region** | **Combined** | **CD8+ epitope** | **CD4+ epitope** |
| --- | --- | --- | --- |
| **World** | 96.21% | 75.41% | 84.58% |
| **East Asia** | 95.38% | 68.83% | 85.18% |
| [**Northeast Asia**](https://tools.iedb.org/population/result/#Northeast%20Asia) | 90.26% | 51.06% | 80.11% |
| [**South Asia**](https://tools.iedb.org/population/result/#South%20Asia) | 91.96% | 55.07% | 82.11% |
| [**Southeast Asia**](https://tools.iedb.org/population/result/#Southeast%20Asia) | 91.36% | 49.60% | 82.85% |
| [**Southwest Asia**](https://tools.iedb.org/population/result/#Southwest%20Asia) | 87.52% | 65.03% | 64.32% |
| [**Europe**](https://tools.iedb.org/population/result/#Europe) | 97.46% | 85.07% | 82.97% |
| [**East Africa**](https://tools.iedb.org/population/result/#East%20Africa) | 99.06% | 71.25% | 96.72% |
| [**West Africa**](https://tools.iedb.org/population/result/#West%20Africa) | 98.94% | 73.82% | 95.95% |
| [**Central Africa**](https://tools.iedb.org/population/result/#Central%20Africa) | 98.10% | 65.37% | 94.51% |
| [**North Africa**](https://tools.iedb.org/population/result/#North%20Africa) | 94.04% | 75.28% | 75.90% |
| [**South Africa**](https://tools.iedb.org/population/result/#South%20Africa) | 63.10% | 62.43% | 1.79% |
| [**West Indies**](https://tools.iedb.org/population/result/#West%20Indies) | 97.10% | 73.82% | 84.73% |
| [**North America**](https://tools.iedb.org/population/result/#North%20America) | 96.07% | 81.67% | 78.56% |
| [**Central America**](https://tools.iedb.org/population/result/#Central%20America) | 55.01% | 5.69% | 52.30% |
| [**South America**](https://tools.iedb.org/population/result/#South%20America) | 90.76% | 47.71% | 82.34% |
| [**Oceania**](https://tools.iedb.org/population/result/#Oceania) | 88.07% | 35.13% | 81.60% |

**Supplementary Table 5.** The Disulfide by Design 2 v2.13 server predicted disulfide bond candidates in the designed vaccine structure. (MB-saVaxI)

| **Res1 Chain** | **Res1 Seq #** | **Res1 AA** | **Res2 Chain** | **Res2 Seq #** | **Res2 AA** | **Chi3** | **Energy** | **Sum B-Factors** |
| --- | --- | --- | --- | --- | --- | --- | --- | --- |
| A | 4 | LEU | A | 10 | LEU | 107.61 | 3.9 | 0 |
| A | 16 | MET | A | 20 | GLU | 123.27 | 3.48 | 0 |
| A | 35 | THR | A | 44 | ALA | -103.38 | 4.57 | 0 |
| A | 37 | ALA | A | 49 | PRO | 80.51 | 3.56 | 0 |
| A | 38 | ALA | A | 43 | ALA | -100.93 | 4.42 | 0 |
| A | 39 | PRO | A | 160 | ALA | -93.76 | 1.64 | 0 |
| A | 40 | VAL | A | 160 | ALA | 118.55 | 5.31 | 0 |
| A | 51 | GLY | A | 162 | ALA | -76.04 | 3.93 | 0 |
| A | 57 | ALA | A | 62 | GLU | 119.96 | 4.97 | 0 |
| A | 62 | GLU | A | 107 | LYS | 108.32 | 5.13 | 0 |
| A | 68 | GLU | A | 126 | THR | 118.84 | 6.58 | 0 |
| A | 73 | LYS | A | 76 | GLY | -109.53 | 6.6 | 0 |
| A | 89 | GLY | A | 92 | GLU | 114.34 | 3.6 | 0 |
| A | 108 | VAL | A | 112 | ALA | 97.3 | 1.59 | 0 |
| A | 120 | LEU | A | 125 | ALA | 119.85 | 4.63 | 0 |
| A | 137 | GLY | A | 141 | GLU | 121.82 | 4.88 | 0 |
| A | 143 | LEU | A | 184 | VAL | 77.78 | 4.59 | 0 |
| A | 146 | ALA | A | 188 | LEU | -72.09 | 4.16 | 0 |
| A | 150 | LEU | A | 170 | ALA | -109.67 | 1.35 | 0 |
| A | 157 | ALA | A | 162 | ALA | -58.79 | 2.46 | 0 |
| A | 163 | LEU | A | 165 | VAL | 89.95 | 2.71 | 0 |
| A | 165 | VAL | A | 198 | VAL | 118.96 | 3.82 | 0 |
| A | 173 | LEU | A | 187 | LEU | 118.53 | 7.4 | 0 |
| A | 196 | ALA | A | 276 | LEU | 119.23 | 4.45 | 0 |
| A | 208 | THR | A | 212 | THR | -76.26 | 6.89 | 0 |
| A | 210 | GLU | A | 320 | SER | -85.12 | 5.03 | 0 |
| A | 224 | PRO | A | 227 | GLY | 118.48 | 2.38 | 0 |
| A | 248 | HIS | A | 251 | GLN | 111.46 | 1.07 | 0 |
| A | 273 | ILE | A | 279 | GLY | 90.31 | 4.84 | 0 |
| A | 299 | ASP | A | 302 | PHE | 99.33 | 3.04 | 0 |
| A | 335 | ASN | A | 338 | THR | 111.05 | 1.53 | 0 |
| A | 379 | PRO | A | 387 | GLY | 106.46 | 1.19 | 0 |
| A | 386 | GLU | A | 389 | GLU | 105.4 | 3.51 | 0 |
| A | 409 | ASP | A | 412 | PHE | 94.49 | 1 | 0 |

**Supplementary Table 6.** The Disulfide by Design 2 v2.13 server predicted disulfide bond candidates in the designed vaccine structure. (MB-saVaxII)

| **Res1 Chain** | **Res1 Seq #** | **Res1 AA** | **Res2 Chain** | **Res2 Seq #** | **Res2 AA** | **Chi3** | **Energy** | **Sum B-Factors** |
| --- | --- | --- | --- | --- | --- | --- | --- | --- |
| A | 11 | ASP | A | 12 | ALA | 123.96 | 6.31 | 17.89 |
| A | 43 | ALA | A | 154 | LEU | 97.59 | 2.45 | 18.35 |
| A | 49 | PRO | A | 50 | ALA | 122.07 | 4.55 | 18.91 |
| A | 49 | PRO | A | 191 | GLY | -108.04 | 5.53 | 20.74 |
| A | 61 | SER | A | 136 | ASP | 80.31 | 3.86 | 18.88 |
| A | 64 | ASP | A | 130 | LYS | 117.7 | 5.42 | 17.6 |
| A | 65 | VAL | A | 129 | VAL | -97.82 | 2.63 | 16.51 |
| A | 67 | LEU | A | 125 | ALA | -93.98 | 5.35 | 16.73 |
| A | 68 | GLU | A | 101 | PRO | 121.25 | 5.82 | 17.83 |
| A | 70 | ALA | A | 71 | GLY | 102.12 | 4.82 | 17.62 |
| A | 70 | ALA | A | 125 | ALA | 115.41 | 4.86 | 17.03 |
| A | 82 | ARG | A | 93 | ALA | 77.61 | 3.19 | 16.74 |
| A | 84 | ILE | A | 116 | ALA | 103.25 | 5.01 | 16.71 |
| A | 86 | SER | A | 87 | GLY | 101.31 | 3.06 | 17.17 |
| A | 100 | ALA | A | 101 | PRO | 92.27 | 1.12 | 18.58 |
| A | 104 | LEU | A | 105 | LEU | -108.19 | 2.89 | 17.21 |
| A | 135 | LYS | A | 138 | LEU | -97.94 | 4.85 | 19.1 |
| A | 161 | PRO | A | 162 | PHE | 100.16 | 1.6 | 18.57 |
| A | 169 | ALA | A | 178 | SER | 97.69 | 4.44 | 18.72 |
| A | 175 | PRO | A | 179 | ALA | -100.11 | 4.4 | 19.84 |
| A | 181 | ALA | A | 220 | ASP | -62.07 | 8.74 | 18.28 |
| A | 184 | ALA | A | 220 | ASP | 102.26 | 4.3 | 18.6 |
| A | 201 | GLY | A | 203 | PHE | 105.03 | 4.33 | 22.01 |
| A | 204 | LEU | A | 205 | ALA | -85.03 | 4.53 | 18.13 |
| A | 209 | TYR | A | 214 | LEU | -102.23 | 1.12 | 18.22 |
| A | 243 | ALA | A | 315 | TYR | -71.33 | 2.68 | 20.58 |
| A | 243 | ALA | A | 316 | LYS | 88.07 | 2.5 | 21.59 |
| A | 253 | LYS | A | 254 | THR | -76.35 | 0.78 | 19.62 |
| A | 257 | ASN | A | 340 | LYS | -68.89 | 7.02 | 18.04 |
| A | 269 | GLY | A | 337 | LYS | -74.83 | 2.97 | 18.23 |
| A | 270 | PRO | A | 336 | TYR | -107.2 | 2.91 | 18.21 |
| A | 291 | GLY | A | 292 | HIS | -109.48 | 3.43 | 26.79 |
| A | 307 | GLY | A | 348 | PRO | -66.99 | 5.92 | 22.73 |
| A | 319 | LYS | A | 320 | SER | 91.74 | 3.2 | 22.2 |

**Supplementary Table 7.** Dataset of predicted structural B-lymphocyte epitopes from the MB-saVaxI using the ElliPro server.

| **No.** | **Residues** | **Number of residues** | **Score** |
| --- | --- | --- | --- |
| 1 | A:A356, A:S357, A:P358, A:N359, A:K360, A:K361, A:Q362, A:K363 | 8 | 0.887 |
| 2 | A:K27, A:E30, A:T31, A:F32 | 4 | 0.805 |
| 3 | A:P408, A:D409, A:M410, A:S411, A:F412, A:D413, A:K414, A:K415, A:M416, A:A417, A:S418, A:S419, A:S420, A:N421, A:Y422, A:N423, A:K424, A:K425, A:Y426, A:K427, A:S428, A:G429, A:K430, A:S431, A:G432, A:H433, A:H434, A:H435 | 28 | 0.763 |
| 4 | A:E58, A:E59, A:Q60, A:S61, A:E62, A:F63, A:D64, A:V65, A:I66, A:L67, A:E68, A:A69, A:A70, A:G71, A:D72, A:K73, A:K74, A:I75, A:G76, A:V77, A:I78, A:K79, A:V80, A:V81, A:R82, A:E83, A:I84, A:V85, A:S86, A:G87, A:L88, A:G89, A:L90, A:K91, A:A93, A:K94, A:D95, A:V97, A:D98, A:G99, A:A100, A:P101, A:K102, A:P103, A:L104, A:L105, A:V108, A:A109, A:K110, A:E111, A:A112, A:A113, A:D114, A:E115, A:A116, A:K117, A:A118, A:K119, A:L120, A:E121, A:A122, A:A123, A:G124, A:A125, A:T126, A:V127, A:T128, A:V129, A:K130, A:E131, A:A132, A:A133, A:A134, A:K135, A:F136, A:G137, A:P138 | 77 | 0.731 |
| 5 | A:G243, A:P244, A:G245, A:P246, A:G247, A:H248, A:H250, A:Q251, A:E252, A:I253, A:Q254, A:A255, A:I256, A:A257, A:E258, A:D259, A:D260, A:E261, A:E262, A:G263, A:P264, A:G265, A:P266, A:G267, A:L268, A:N269, A:G294, A:D295, A:L296, A:N297, A:L298, A:D299, A:D300, A:Q301, A:F302, A:G303, A:P304, A:G305, A:P306, A:G307, A:E308, A:T322, A:G323, A:P324, A:G325, A:P326, A:G327, A:I328, A:D329, A:F330, A:D331, A:P332, A:V333, A:P334, A:N335, A:T336, A:K337, A:T338, A:I339, A:F340 | 60 | 0.713 |
| 6 | A:K401, A:Q404, A:A405, A:D406 | 4 | 0.704 |
| 7 | A:D341, A:E342, A:S343, A:S344, A:S345, A:S346, A:G347, A:A348, A:A350, A:D353, A:Q354, A:V364, A:A365, A:D366, A:S367, A:P368, A:L369, A:E370, A:A371, A:S372, A:K373, A:K374, A:T375, A:G376, A:V377, A:P378 | 26 | 0.682 |
| 8 | A:D222, A:G223, A:P224, A:G225, A:P226, A:E229 | 6 | 0.58 |
| 9 | A:M1, A:K3, A:L4, A:S5, A:T6, A:D7, A:E8, A:L9, A:D11, A:A12, A:I273, A:N274, A:N275, A:L276, A:E277, A:H278 | 16 | 0.535 |

**Supplementary Table 8.** Dataset of predicted structural B-lymphocyte epitopes from the MB-saVaxII using the ElliPro server.

| **No.** | **Residues** | **Number of residues** | **Score** |
| --- | --- | --- | --- |
| 1 | A:M1, A:A2, A:K3, A:L4, A:S5, A:T6, A:D7, A:E8, A:L9, A:L10, A:D11, A:A12, A:F13, A:K14, A:E15, A:M16, A:T17, A:L18, A:L19, A:E20, A:L21, A:S22, A:D23, A:F24, A:V25, A:K26, A:K27, A:F28, A:E29, A:E30, A:T31, A:F32, A:E33, A:V34, A:T35, A:A36, A:A37, A:A38, A:P39 | 39 | 0.839 |
| 2 | A:N376, A:N377, A:R378, A:Q379, A:V380, A:V381, A:L382, A:Y384, A:A385, A:N386, A:G387, A:P388, A:G389, A:P390, A:G391, A:I392, A:D393, A:F394, A:I403, A:D405, A:E406, A:S407, A:S408, A:S409, A:S410, A:G411, A:A412, A:S413, A:A414, A:E415, A:E416, A:D417, A:Q418, A:H419, A:A420, A:S421, A:P422, A:N423, A:K424, A:K425, A:T426, A:K427, A:P428, A:T429, A:A430, A:P431, A:H432, A:V433, A:K458, A:K459, A:P460, A:E461, A:N462, A:F463, A:P464, A:L465, A:C466, A:C467, A:H468, A:H469, A:H470, A:H471 | 62 | 0.782 |
| 3 | A:V165, A:L166, A:N167, A:L168, A:A169, A:A170, A:Y171, A:G172, A:L173, A:Y174, A:P175, A:Q176, A:L177, A:S178, A:A179 | 15 | 0.737 |
| 4 | A:I258, A:S259, A:V260, A:T261, A:D262, A:P263, A:S264, A:G265, A:K266, A:G267, A:P268, A:G269, A:P270, A:G271, A:E272, A:A335, A:Y336, A:K337, A:S338, A:G339 | 20 | 0.699 |
| 5 | A:Q273, A:G274, A:I275, A:V276, A:T277, A:Y278, A:P279, A:L283, A:T284, A:T285, A:G286, A:G287, A:P288, A:G289, A:P290, A:G291, A:H292, A:G293, A:E294 | 19 | 0.665 |
| 6 | A:L447, A:A449, A:V450, A:P451, A:P452, A:N453, A:P454, A:T455, A:I456, A:D457 | 10 | 0.662 |
| 7 | A:A50, A:G51, A:A52, A:V54, A:E55, A:A56, A:A57, A:E58, A:E59, A:Q60, A:S61, A:E62, A:F63, A:D64, A:K107, A:T128, A:V129, A:K130, A:E131, A:A132, A:A133, A:A134, A:K135, A:D136, A:D137, A:L138, A:A139, A:A140, A:G141, A:L142, A:W144, A:A145, A:H148 | 33 | 0.642 |
| 8 | A:S223, A:V224, A:K225, A:E226, A:G227, A:M228, A:A229, A:A230, A:Y231, A:S232, A:G233, A:Q234, A:K235, A:V236, A:A237, A:D238, A:S239, A:P240, A:L241, A:E242, A:C243, A:S244, A:L302, A:N303, A:L304, A:S305, A:G306, A:G307, A:P308, A:G309, A:P310, A:G311, A:K312, A:I313, A:A314, A:Y315, A:C316, A:S317, A:G318, A:K319, A:S320, A:G327, A:G329, A:P330, A:A343, A:F344, A:L345, A:D346, A:G347, A:P348, A:G349 | 51 | 0.565 |
| 9 | A:D365, A:Q366, A:G369, A:P370, A:G371, A:L372, A:N373, A:G374, A:F375 | 9 | 0.548 |

**Supplementary Table 9.** The comparison of different parameters between the I-TASSER and AlphaFold3-predicted MB-saVaxI and MB-saVaxII 3D structures.

| **Parameter** | **I-TASSER Server (MB-saVaxI)** | **AlphaFold3 Server (MB-saVaxI)** | **I-TASSER Server (MB-saVaxII)** | **AlphaFold3 Server (MB-saVaxII)** |
| --- | --- | --- | --- | --- |
|  | **Raw Model** | | | |
| C- score | -2.36 | 0.27 | -2.25 | 0.24 |
|  | **Refined Model** | | | |
| Z-score | -7.75 | -5.29 | -5.82 | -3.31 |
| Ramachandran values | **Most favoured Regions** | | | |
|  | 93.349% | 98.624% | 91.702% | 97.447% |
|  | **Allowed Regions** | | | |
|  | 4.817% | 1.147% | 7.021% | 2.34% |
|  | **Disallowed Regions** | | | |
|  | 1.835% | 0.229% | 1.277% | 0.213% |
