# Supplementary material for: Immunoinformatics and reverse vaccinology design of self-amplifying RNA vaccine against marburg virus with structural dynamics and immune simulation analysis
Source: PLoS One. 2026 Oct 23;21(10):e0361136. doi: 10.1371/journal.pone.0361136 (PMC13693908; doi:10.1371/journal.pone.0361136)
Supplement: Supplementary file 3 [file pone.0361136.s002.docx]

**Protein RMSD**

| **frame** | **Time (ns)** | **MB-mVax-TLR2** | **MB-mVax-TLR4** |
| --- | --- | --- | --- |
| 0 | 0 | 0 | 0 |
| 1 | 0.2 | 3.283 | 2.771 |
| 2 | 0.4 | 3.723 | 3.205 |
| 3 | 0.6 | 4.177 | 3.844 |
| 4 | 0.8 | 4.02 | 3.642 |
| 5 | 1 | 4.674 | 3.706 |
| 6 | 1.2 | 4.77 | 3.992 |
| 7 | 1.4 | 4.449 | 4.036 |
| 8 | 1.6 | 4.545 | 4.563 |
| 9 | 1.8 | 4.857 | 4.467 |
| 10 | 2 | 4.902 | 4.258 |
| 11 | 2.2 | 4.843 | 4.566 |
| 12 | 2.4 | 4.834 | 4.499 |
| 13 | 2.6 | 5.065 | 4.281 |
| 14 | 2.8 | 5.736 | 3.917 |
| 15 | 3 | 6.047 | 3.827 |
| 16 | 3.2 | 5.461 | 3.881 |
| 17 | 3.4 | 6.05 | 4.005 |
| 18 | 3.6 | 6.197 | 4.099 |
| 19 | 3.8 | 6.494 | 4.08 |
| 20 | 4 | 6.379 | 3.863 |
| 21 | 4.2 | 6.675 | 4.057 |
| 22 | 4.4 | 6.668 | 4.305 |
| 23 | 4.6 | 6.912 | 4.243 |
| 24 | 4.8 | 7.352 | 4.397 |
| 25 | 5 | 7.063 | 4.32 |
| 26 | 5.2 | 6.491 | 4.655 |
| 27 | 5.4 | 6.371 | 4.614 |
| 28 | 5.6 | 6.429 | 4.457 |
| 29 | 5.8 | 7.22 | 4.534 |
| 30 | 6 | 6.123 | 4.654 |
| 31 | 6.2 | 6.077 | 4.629 |
| 32 | 6.4 | 6.155 | 4.578 |
| 33 | 6.6 | 6.252 | 4.688 |
| 34 | 6.8 | 5.999 | 4.704 |
| 35 | 7 | 6.567 | 4.672 |
| 36 | 7.2 | 6.49 | 4.78 |
| 37 | 7.4 | 6.165 | 4.963 |
| 38 | 7.6 | 6.433 | 4.975 |
| 39 | 7.8 | 6.12 | 4.981 |
| 40 | 8 | 6.193 | 4.895 |
| 41 | 8.2 | 6.469 | 4.852 |
| 42 | 8.4 | 6.536 | 4.992 |
| 43 | 8.6 | 6.267 | 4.837 |
| 44 | 8.8 | 6.327 | 4.683 |
| 45 | 9 | 7.227 | 4.968 |
| 46 | 9.2 | 7.691 | 5.179 |
| 47 | 9.4 | 7.129 | 5.095 |
| 48 | 9.6 | 6.524 | 5.11 |
| 49 | 9.8 | 5.83 | 5.084 |
| 50 | 10 | 6 | 4.953 |
| 51 | 10.2 | 6.092 | 4.857 |
| 52 | 10.4 | 6.286 | 4.651 |
| 53 | 10.6 | 6.14 | 4.632 |
| 54 | 10.8 | 6.607 | 4.743 |
| 55 | 11 | 6.789 | 4.566 |
| 56 | 11.2 | 6.684 | 4.6 |
| 57 | 11.4 | 7.156 | 4.544 |
| 58 | 11.6 | 7.516 | 4.423 |
| 59 | 11.8 | 7.413 | 4.371 |
| 60 | 12 | 8.159 | 4.494 |
| 61 | 12.2 | 7.971 | 4.552 |
| 62 | 12.4 | 7.898 | 4.346 |
| 63 | 12.6 | 7.951 | 4.696 |
| 64 | 12.8 | 8.183 | 4.41 |
| 65 | 13 | 8.506 | 4.373 |
| 66 | 13.2 | 8.719 | 4.517 |
| 67 | 13.4 | 8.788 | 4.53 |
| 68 | 13.6 | 8.066 | 4.522 |
| 69 | 13.8 | 8.012 | 4.609 |
| 70 | 14 | 8.085 | 4.822 |
| 71 | 14.2 | 8.19 | 4.747 |
| 72 | 14.4 | 7.911 | 4.892 |
| 73 | 14.6 | 8.235 | 4.661 |
| 74 | 14.8 | 8.492 | 4.689 |
| 75 | 15 | 8.575 | 4.534 |
| 76 | 15.2 | 8.794 | 4.655 |
| 77 | 15.4 | 8.427 | 4.757 |
| 78 | 15.6 | 8.616 | 4.36 |
| 79 | 15.8 | 9.27 | 4.599 |
| 80 | 16 | 8.719 | 4.815 |
| 81 | 16.2 | 8.013 | 4.945 |
| 82 | 16.4 | 7.889 | 4.896 |
| 83 | 16.6 | 7.697 | 5.112 |
| 84 | 16.8 | 7.846 | 4.72 |
| 85 | 17 | 7.996 | 4.674 |
| 86 | 17.2 | 8.137 | 5.044 |
| 87 | 17.4 | 7.804 | 5.162 |
| 88 | 17.6 | 8.007 | 4.919 |
| 89 | 17.8 | 7.454 | 4.725 |
| 90 | 18 | 7.941 | 4.701 |
| 91 | 18.2 | 7.477 | 4.463 |
| 92 | 18.4 | 7.478 | 4.787 |
| 93 | 18.6 | 7.592 | 4.484 |
| 94 | 18.8 | 7.795 | 5.259 |
| 95 | 19 | 8.215 | 4.835 |
| 96 | 19.2 | 8.631 | 4.808 |
| 97 | 19.4 | 8.54 | 4.972 |
| 98 | 19.6 | 8.504 | 4.627 |
| 99 | 19.8 | 8.492 | 4.696 |
| 100 | 20 | 9.062 | 4.986 |
| 101 | 20.2 | 9.383 | 4.914 |
| 102 | 20.4 | 9.712 | 4.88 |
| 103 | 20.6 | 9.498 | 4.824 |
| 104 | 20.8 | 8.653 | 4.709 |
| 105 | 21 | 8.575 | 5.015 |
| 106 | 21.2 | 8.939 | 4.924 |
| 107 | 21.4 | 8.825 | 5.044 |
| 108 | 21.6 | 8.742 | 5.14 |
| 109 | 21.8 | 8.778 | 4.779 |
| 110 | 22 | 8.515 | 5.036 |
| 111 | 22.2 | 8.069 | 5.003 |
| 112 | 22.4 | 8.441 | 5.007 |
| 113 | 22.6 | 8.833 | 4.808 |
| 114 | 22.8 | 8.959 | 5.185 |
| 115 | 23 | 8.491 | 5.206 |
| 116 | 23.2 | 8.862 | 4.998 |
| 117 | 23.4 | 8.459 | 5.084 |
| 118 | 23.6 | 9.138 | 4.945 |
| 119 | 23.8 | 7.954 | 4.975 |
| 120 | 24 | 8.622 | 4.582 |
| 121 | 24.2 | 8.339 | 4.831 |
| 122 | 24.4 | 8.625 | 4.883 |
| 123 | 24.6 | 8.006 | 4.57 |
| 124 | 24.8 | 8.481 | 4.63 |
| 125 | 25 | 8.774 | 4.714 |
| 126 | 25.2 | 8.056 | 4.895 |
| 127 | 25.4 | 7.541 | 5.086 |
| 128 | 25.6 | 8.128 | 4.778 |
| 129 | 25.8 | 8.476 | 4.973 |
| 130 | 26 | 8.28 | 4.895 |
| 131 | 26.2 | 8.474 | 4.789 |
| 132 | 26.4 | 8.95 | 4.73 |
| 133 | 26.6 | 8.767 | 4.969 |
| 134 | 26.8 | 8.45 | 4.955 |
| 135 | 27 | 8.648 | 5.13 |
| 136 | 27.2 | 8.916 | 5.175 |
| 137 | 27.4 | 9.303 | 5.014 |
| 138 | 27.6 | 9.153 | 4.981 |
| 139 | 27.8 | 8.858 | 4.961 |
| 140 | 28 | 8.993 | 4.932 |
| 141 | 28.2 | 8.695 | 5.135 |
| 142 | 28.4 | 9.271 | 4.846 |
| 143 | 28.6 | 9.537 | 4.854 |
| 144 | 28.8 | 10.037 | 4.815 |
| 145 | 29 | 8.979 | 4.825 |
| 146 | 29.2 | 8.716 | 4.948 |
| 147 | 29.4 | 9.122 | 4.821 |
| 148 | 29.6 | 8.577 | 4.9 |
| 149 | 29.8 | 8.682 | 4.851 |
| 150 | 30 | 8.916 | 5.092 |
| 151 | 30.2 | 8.733 | 5.52 |
| 152 | 30.4 | 8.302 | 5.39 |
| 153 | 30.6 | 8.479 | 5.092 |
| 154 | 30.8 | 9.079 | 5.005 |
| 155 | 31 | 9.2 | 5.179 |
| 156 | 31.2 | 9.203 | 4.881 |
| 157 | 31.4 | 9.16 | 5.068 |
| 158 | 31.6 | 8.975 | 4.99 |
| 159 | 31.8 | 8.941 | 5.081 |
| 160 | 32 | 8.58 | 5.225 |
| 161 | 32.2 | 8.783 | 5.542 |
| 162 | 32.4 | 8.358 | 5.363 |
| 163 | 32.6 | 8.326 | 4.997 |
| 164 | 32.8 | 8.366 | 5.429 |
| 165 | 33 | 8.016 | 5.237 |
| 166 | 33.2 | 8.301 | 5.597 |
| 167 | 33.4 | 8.532 | 5.337 |
| 168 | 33.6 | 8.929 | 5.517 |
| 169 | 33.8 | 8.94 | 5.399 |
| 170 | 34 | 9.228 | 5.341 |
| 171 | 34.2 | 8.618 | 5.432 |
| 172 | 34.4 | 7.98 | 5.444 |
| 173 | 34.6 | 8.483 | 5.493 |
| 174 | 34.8 | 8.194 | 5.562 |
| 175 | 35 | 8.883 | 5.5 |
| 176 | 35.2 | 8.937 | 5.472 |
| 177 | 35.4 | 8.314 | 5.141 |
| 178 | 35.6 | 8.677 | 5.182 |
| 179 | 35.8 | 8.764 | 4.86 |
| 180 | 36 | 8.815 | 5.29 |
| 181 | 36.2 | 8.826 | 5.296 |
| 182 | 36.4 | 8.728 | 5.474 |
| 183 | 36.6 | 8.971 | 5.049 |
| 184 | 36.8 | 8.731 | 4.985 |
| 185 | 37 | 8.597 | 5.266 |
| 186 | 37.2 | 8.498 | 5.275 |
| 187 | 37.4 | 8.671 | 5.054 |
| 188 | 37.6 | 9.301 | 5.096 |
| 189 | 37.8 | 9.417 | 4.855 |
| 190 | 38 | 9.465 | 4.925 |
| 191 | 38.2 | 9.309 | 5.256 |
| 192 | 38.4 | 9.152 | 5.334 |
| 193 | 38.6 | 9.219 | 5.44 |
| 194 | 38.8 | 8.738 | 5.413 |
| 195 | 39 | 8.895 | 5.429 |
| 196 | 39.2 | 8.878 | 5.419 |
| 197 | 39.4 | 9.026 | 5.493 |
| 198 | 39.6 | 9.435 | 5.528 |
| 199 | 39.8 | 9.434 | 5.357 |
| 200 | 40 | 9.589 | 5.305 |
| 201 | 40.2 | 10.003 | 5.447 |
| 202 | 40.4 | 9.674 | 5.566 |
| 203 | 40.6 | 9.113 | 5.328 |
| 204 | 40.8 | 9.66 | 5.177 |
| 205 | 41 | 9.672 | 5.331 |
| 206 | 41.2 | 9.865 | 5.208 |
| 207 | 41.4 | 9.899 | 5.293 |
| 208 | 41.6 | 9.646 | 5.649 |
| 209 | 41.8 | 9.903 | 5.502 |
| 210 | 42 | 9.901 | 5.459 |
| 211 | 42.2 | 9.699 | 5.436 |
| 212 | 42.4 | 9.579 | 5.237 |
| 213 | 42.6 | 9.809 | 5.558 |
| 214 | 42.8 | 9.538 | 5.31 |
| 215 | 43 | 9.651 | 5.484 |
| 216 | 43.2 | 9.734 | 5.471 |
| 217 | 43.4 | 9.434 | 5.533 |
| 218 | 43.6 | 9.989 | 5.448 |
| 219 | 43.8 | 9.107 | 5.692 |
| 220 | 44 | 9.767 | 5.607 |
| 221 | 44.2 | 9.404 | 5.693 |
| 222 | 44.4 | 9.495 | 5.668 |
| 223 | 44.6 | 9.106 | 5.684 |
| 224 | 44.8 | 8.86 | 5.506 |
| 225 | 45 | 9.57 | 5.643 |
| 226 | 45.2 | 9.162 | 5.513 |
| 227 | 45.4 | 9.319 | 5.514 |
| 228 | 45.6 | 9.285 | 5.374 |
| 229 | 45.8 | 9.921 | 5.562 |
| 230 | 46 | 9.56 | 5.643 |
| 231 | 46.2 | 9.461 | 5.746 |
| 232 | 46.4 | 9.266 | 5.774 |
| 233 | 46.6 | 9.522 | 5.791 |
| 234 | 46.8 | 9.321 | 5.711 |
| 235 | 47 | 9.554 | 5.585 |
| 236 | 47.2 | 9.451 | 5.539 |
| 237 | 47.4 | 9.607 | 5.756 |
| 238 | 47.6 | 9.496 | 5.478 |
| 239 | 47.8 | 9.338 | 5.831 |
| 240 | 48 | 9.471 | 5.721 |
| 241 | 48.2 | 9.2 | 5.834 |
| 242 | 48.4 | 9.309 | 5.754 |
| 243 | 48.6 | 9.944 | 5.462 |
| 244 | 48.8 | 9.31 | 5.554 |
| 245 | 49 | 8.822 | 5.623 |
| 246 | 49.2 | 8.67 | 5.958 |
| 247 | 49.4 | 8.806 | 5.663 |
| 248 | 49.6 | 9.112 | 5.551 |
| 249 | 49.8 | 8.801 | 5.492 |
| 250 | 50 | 9.075 | 5.78 |
| 251 | 50.2 | 8.947 | 5.622 |
| 252 | 50.4 | 8.66 | 5.778 |
| 253 | 50.6 | 8.662 | 5.47 |
| 254 | 50.8 | 8.97 | 5.582 |
| 255 | 51 | 8.806 | 5.481 |
| 256 | 51.2 | 9.218 | 5.437 |
| 257 | 51.4 | 8.824 | 5.504 |
| 258 | 51.6 | 8.843 | 5.639 |
| 259 | 51.8 | 9.223 | 5.605 |
| 260 | 52 | 9.181 | 5.282 |
| 261 | 52.2 | 9.577 | 5.352 |
| 262 | 52.4 | 9.233 | 5.416 |
| 263 | 52.6 | 9.127 | 5.531 |
| 264 | 52.8 | 9.596 | 5.6 |
| 265 | 53 | 9.157 | 5.38 |
| 266 | 53.2 | 9.243 | 5.419 |
| 267 | 53.4 | 9.005 | 5.784 |
| 268 | 53.6 | 8.729 | 5.46 |
| 269 | 53.8 | 9.153 | 5.391 |
| 270 | 54 | 9.111 | 5.148 |
| 271 | 54.2 | 8.851 | 5.424 |
| 272 | 54.4 | 9.305 | 5.401 |
| 273 | 54.6 | 8.654 | 5.292 |
| 274 | 54.8 | 9.26 | 5.216 |
| 275 | 55 | 9.179 | 5.281 |
| 276 | 55.2 | 9.112 | 5.444 |
| 277 | 55.4 | 9.117 | 5.399 |
| 278 | 55.6 | 8.704 | 5.413 |
| 279 | 55.8 | 8.731 | 5.384 |
| 280 | 56 | 8.974 | 5.682 |
| 281 | 56.2 | 9.248 | 5.552 |
| 282 | 56.4 | 8.929 | 5.482 |
| 283 | 56.6 | 9.143 | 5.354 |
| 284 | 56.8 | 8.998 | 5.285 |
| 285 | 57 | 8.859 | 5.367 |
| 286 | 57.2 | 8.777 | 5.574 |
| 287 | 57.4 | 8.813 | 5.483 |
| 288 | 57.6 | 8.911 | 5.399 |
| 289 | 57.8 | 8.722 | 5.464 |
| 290 | 58 | 8.904 | 5.594 |
| 291 | 58.2 | 8.725 | 5.511 |
| 292 | 58.4 | 8.818 | 5.259 |
| 293 | 58.6 | 8.336 | 5.271 |
| 294 | 58.8 | 8.691 | 5.199 |
| 295 | 59 | 8.954 | 5.215 |
| 296 | 59.2 | 8.673 | 5.179 |
| 297 | 59.4 | 8.289 | 5.239 |
| 298 | 59.6 | 8.742 | 5.302 |
| 299 | 59.8 | 8.57 | 5.133 |
| 300 | 60 | 8.623 | 5.06 |
| 301 | 60.2 | 8.439 | 5.331 |
| 302 | 60.4 | 8.325 | 5.201 |
| 303 | 60.6 | 7.87 | 5.037 |
| 304 | 60.8 | 8.449 | 5.435 |
| 305 | 61 | 8.311 | 5.308 |
| 306 | 61.2 | 8.665 | 5.364 |
| 307 | 61.4 | 8.367 | 5.359 |
| 308 | 61.6 | 8.372 | 5.391 |
| 309 | 61.8 | 8.368 | 5.383 |
| 310 | 62 | 8.234 | 5.387 |
| 311 | 62.2 | 8.242 | 5.41 |
| 312 | 62.4 | 8.4 | 5.23 |
| 313 | 62.6 | 8.259 | 5.246 |
| 314 | 62.8 | 8.221 | 5.005 |
| 315 | 63 | 8.305 | 5.285 |
| 316 | 63.2 | 8.137 | 5.242 |
| 317 | 63.4 | 8.479 | 5.081 |
| 318 | 63.6 | 8.263 | 5.317 |
| 319 | 63.8 | 8.581 | 5.249 |
| 320 | 64 | 8.522 | 5.213 |
| 321 | 64.2 | 8.634 | 5.335 |
| 322 | 64.4 | 8.409 | 5.156 |
| 323 | 64.6 | 9.154 | 5.205 |
| 324 | 64.8 | 8.787 | 5.297 |
| 325 | 65 | 9.053 | 5.454 |
| 326 | 65.2 | 9.059 | 5.238 |
| 327 | 65.4 | 9.765 | 5.095 |
| 328 | 65.6 | 9.328 | 5.216 |
| 329 | 65.8 | 8.926 | 5.473 |
| 330 | 66 | 9.018 | 5.427 |
| 331 | 66.2 | 9.028 | 5.495 |
| 332 | 66.4 | 8.914 | 5.38 |
| 333 | 66.6 | 8.768 | 5.456 |
| 334 | 66.8 | 8.773 | 5.165 |
| 335 | 67 | 8.98 | 5.294 |
| 336 | 67.2 | 9.228 | 5.429 |
| 337 | 67.4 | 9.154 | 5.468 |
| 338 | 67.6 | 9.187 | 5.496 |
| 339 | 67.8 | 9.356 | 5.435 |
| 340 | 68 | 9.455 | 5.613 |
| 341 | 68.2 | 9.449 | 5.601 |
| 342 | 68.4 | 9.377 | 5.488 |
| 343 | 68.6 | 9.296 | 5.579 |
| 344 | 68.8 | 9.713 | 5.378 |
| 345 | 69 | 9.263 | 5.606 |
| 346 | 69.2 | 9.06 | 5.831 |
| 347 | 69.4 | 9.433 | 5.685 |
| 348 | 69.6 | 9.033 | 5.516 |
| 349 | 69.8 | 9.051 | 5.386 |
| 350 | 70 | 8.847 | 5.213 |
| 351 | 70.2 | 9.302 | 5.496 |
| 352 | 70.4 | 9.167 | 5.481 |
| 353 | 70.6 | 9.239 | 5.426 |
| 354 | 70.8 | 9.031 | 5.652 |
| 355 | 71 | 9.083 | 5.659 |
| 356 | 71.2 | 8.819 | 5.66 |
| 357 | 71.4 | 9.222 | 5.531 |
| 358 | 71.6 | 9.126 | 5.553 |
| 359 | 71.8 | 9.208 | 5.505 |
| 360 | 72 | 9.404 | 5.238 |
| 361 | 72.2 | 9.304 | 5.207 |
| 362 | 72.4 | 9.894 | 5.298 |
| 363 | 72.6 | 9.579 | 5.256 |
| 364 | 72.8 | 9.477 | 5.314 |
| 365 | 73 | 9.238 | 5.231 |
| 366 | 73.2 | 9.696 | 5.283 |
| 367 | 73.4 | 9.609 | 5.48 |
| 368 | 73.6 | 9.557 | 5.403 |
| 369 | 73.8 | 9.3 | 5.409 |
| 370 | 74 | 9.424 | 5.331 |
| 371 | 74.2 | 9.285 | 5.269 |
| 372 | 74.4 | 8.753 | 5.248 |
| 373 | 74.6 | 8.651 | 5.162 |
| 374 | 74.8 | 8.879 | 5.156 |
| 375 | 75 | 9.314 | 5.303 |
| 376 | 75.2 | 9.466 | 5.29 |
| 377 | 75.4 | 9.119 | 5.164 |
| 378 | 75.6 | 9.176 | 5.257 |
| 379 | 75.8 | 9.345 | 5.175 |
| 380 | 76 | 9.318 | 5.187 |
| 381 | 76.2 | 9.143 | 5.117 |
| 382 | 76.4 | 10.31 | 5.175 |
| 383 | 76.6 | 9.923 | 5.153 |
| 384 | 76.8 | 9.245 | 5.301 |
| 385 | 77 | 9.023 | 5.314 |
| 386 | 77.2 | 9.266 | 5.304 |
| 387 | 77.4 | 9.459 | 5.227 |
| 388 | 77.6 | 9.682 | 5.332 |
| 389 | 77.8 | 9.297 | 5.318 |
| 390 | 78 | 9.559 | 5.158 |
| 391 | 78.2 | 9.224 | 5.241 |
| 392 | 78.4 | 9.868 | 5.227 |
| 393 | 78.6 | 9.372 | 5.304 |
| 394 | 78.8 | 9.885 | 5.194 |
| 395 | 79 | 10.173 | 5.296 |
| 396 | 79.2 | 10.199 | 5.169 |
| 397 | 79.4 | 9.964 | 5.349 |
| 398 | 79.6 | 9.864 | 5.113 |
| 399 | 79.8 | 9.912 | 5.202 |
| 400 | 80 | 9.765 | 5.158 |
| 401 | 80.2 | 9.941 | 5.211 |
| 402 | 80.4 | 10.168 | 5.164 |
| 403 | 80.6 | 10.032 | 5.334 |
| 404 | 80.8 | 9.973 | 5.319 |
| 405 | 81 | 9.759 | 5.304 |
| 406 | 81.2 | 9.988 | 5.322 |
| 407 | 81.4 | 10.268 | 5.251 |
| 408 | 81.6 | 9.521 | 5.218 |
| 409 | 81.8 | 9.688 | 5.171 |
| 410 | 82 | 9.762 | 5.223 |
| 411 | 82.2 | 9.877 | 5.356 |
| 412 | 82.4 | 9.71 | 5.315 |
| 413 | 82.6 | 9.979 | 5.256 |
| 414 | 82.8 | 9.545 | 5.37 |
| 415 | 83 | 9.391 | 4.949 |
| 416 | 83.2 | 9.624 | 5.225 |
| 417 | 83.4 | 9.693 | 5.125 |
| 418 | 83.6 | 9.951 | 5.372 |
| 419 | 83.8 | 9.499 | 5.234 |
| 420 | 84 | 9.773 | 5.193 |
| 421 | 84.2 | 9.373 | 5.353 |
| 422 | 84.4 | 9.258 | 5.308 |
| 423 | 84.6 | 9.884 | 5.312 |
| 424 | 84.8 | 9.911 | 5.361 |
| 425 | 85 | 9.87 | 5.384 |
| 426 | 85.2 | 9.756 | 5.571 |
| 427 | 85.4 | 9.568 | 5.564 |
| 428 | 85.6 | 9.424 | 5.706 |
| 429 | 85.8 | 9.638 | 5.539 |
| 430 | 86 | 9.441 | 5.619 |
| 431 | 86.2 | 9.86 | 5.516 |
| 432 | 86.4 | 9.839 | 5.852 |
| 433 | 86.6 | 9.927 | 5.65 |
| 434 | 86.8 | 9.983 | 5.615 |
| 435 | 87 | 10.125 | 5.268 |
| 436 | 87.2 | 9.54 | 5.603 |
| 437 | 87.4 | 9.561 | 5.343 |
| 438 | 87.6 | 9.185 | 5.552 |
| 439 | 87.8 | 9.467 | 5.609 |
| 440 | 88 | 9.692 | 5.29 |
| 441 | 88.2 | 9.586 | 5.351 |
| 442 | 88.4 | 9.632 | 5.527 |
| 443 | 88.6 | 9.224 | 5.591 |
| 444 | 88.8 | 9.593 | 5.428 |
| 445 | 89 | 9.3 | 5.401 |
| 446 | 89.2 | 9.034 | 5.463 |
| 447 | 89.4 | 9.634 | 5.348 |
| 448 | 89.6 | 9.578 | 5.289 |
| 449 | 89.8 | 9.425 | 5.324 |
| 450 | 90 | 9.26 | 5.254 |
| 451 | 90.2 | 9.723 | 5.47 |
| 452 | 90.4 | 9.537 | 5.548 |
| 453 | 90.6 | 9.363 | 5.409 |
| 454 | 90.8 | 9.783 | 5.424 |
| 455 | 91 | 9.636 | 5.532 |
| 456 | 91.2 | 9.775 | 5.372 |
| 457 | 91.4 | 10.071 | 5.314 |
| 458 | 91.6 | 10.119 | 5.256 |
| 459 | 91.8 | 10.345 | 5.618 |
| 460 | 92 | 9.584 | 5.591 |
| 461 | 92.2 | 10.013 | 5.515 |
| 462 | 92.4 | 9.894 | 5.479 |
| 463 | 92.6 | 9.622 | 5.594 |
| 464 | 92.8 | 9.463 | 5.558 |
| 465 | 93 | 9.694 | 5.763 |
| 466 | 93.2 | 10.127 | 5.52 |
| 467 | 93.4 | 9.989 | 5.413 |
| 468 | 93.6 | 9.43 | 5.453 |
| 469 | 93.8 | 9.661 | 5.412 |
| 470 | 94 | 9.339 | 5.47 |
| 471 | 94.2 | 9.902 | 5.496 |
| 472 | 94.4 | 9.408 | 5.568 |
| 473 | 94.6 | 9.084 | 5.294 |
| 474 | 94.8 | 9.758 | 5.39 |
| 475 | 95 | 9.027 | 5.403 |
| 476 | 95.2 | 9.502 | 5.369 |
| 477 | 95.4 | 9.389 | 5.598 |
| 478 | 95.6 | 9.178 | 5.754 |
| 479 | 95.8 | 9.019 | 5.565 |
| 480 | 96 | 9.553 | 5.547 |
| 481 | 96.2 | 9.32 | 5.411 |
| 482 | 96.4 | 9.281 | 5.301 |
| 483 | 96.6 | 9.045 | 5.42 |
| 484 | 96.8 | 9.082 | 5.461 |
| 485 | 97 | 9.44 | 5.478 |
| 486 | 97.2 | 9.608 | 5.496 |
| 487 | 97.4 | 9.04 | 5.394 |
| 488 | 97.6 | 9.167 | 5.347 |
| 489 | 97.8 | 9.337 | 5.611 |
| 490 | 98 | 9.305 | 5.406 |
| 491 | 98.2 | 9.464 | 5.443 |
| 492 | 98.4 | 9.2 | 5.293 |
| 493 | 98.6 | 9.229 | 5.276 |
| 494 | 98.8 | 8.776 | 5.427 |
| 495 | 99 | 8.858 | 5.384 |
| 496 | 99.2 | 8.674 | 5.339 |
| 497 | 99.4 | 9.009 | 5.527 |
| 498 | 99.6 | 9.075 | 5.391 |
| 499 | 99.8 | 9.293 | 5.446 |
| 500 | 100 | 9.307 | 5.335 |
| 501 | 100.2 | 9.337 | 5.197 |
| 502 | 100.4 | 9.075 | 5.422 |
| 503 | 100.6 | 8.743 | 5.424 |
| 504 | 100.8 | 8.716 | 5.395 |
| 505 | 101 | 8.935 | 5.095 |
| 506 | 101.2 | 8.847 | 5.24 |
| 507 | 101.4 | 9.19 | 5.284 |
| 508 | 101.6 | 9.002 | 5.563 |
| 509 | 101.8 | 9.126 | 5.362 |
| 510 | 102 | 9.264 | 5.374 |
| 511 | 102.2 | 9.077 | 5.369 |
| 512 | 102.4 | 8.928 | 5.152 |
| 513 | 102.6 | 9.222 | 5.276 |
| 514 | 102.8 | 8.912 | 5.255 |
| 515 | 103 | 9.351 | 5.362 |
| 516 | 103.2 | 8.928 | 5.319 |
| 517 | 103.4 | 9.552 | 5.213 |
| 518 | 103.6 | 9.651 | 5.321 |
| 519 | 103.8 | 9.167 | 5.124 |
| 520 | 104 | 8.87 | 5.25 |
| 521 | 104.2 | 9.257 | 5.319 |
| 522 | 104.4 | 9.316 | 5.142 |
| 523 | 104.6 | 9.034 | 5.074 |
| 524 | 104.8 | 9.073 | 5.34 |
| 525 | 105 | 9.101 | 5.198 |
| 526 | 105.2 | 9.081 | 5.076 |
| 527 | 105.4 | 9.185 | 5.293 |
| 528 | 105.6 | 8.819 | 5.231 |
| 529 | 105.8 | 9.197 | 5.231 |
| 530 | 106 | 9.153 | 5.24 |
| 531 | 106.2 | 9.393 | 5.176 |
| 532 | 106.4 | 9.538 | 5.241 |
| 533 | 106.6 | 9.392 | 5.212 |
| 534 | 106.8 | 9.021 | 5.294 |
| 535 | 107 | 9.024 | 5.22 |
| 536 | 107.2 | 9.086 | 5.239 |
| 537 | 107.4 | 8.956 | 5.305 |
| 538 | 107.6 | 8.572 | 5.28 |
| 539 | 107.8 | 8.831 | 5.327 |
| 540 | 108 | 9.063 | 5.066 |
| 541 | 108.2 | 8.744 | 5.291 |
| 542 | 108.4 | 9.047 | 5.325 |
| 543 | 108.6 | 8.617 | 5.428 |
| 544 | 108.8 | 8.827 | 5.151 |
| 545 | 109 | 8.639 | 5.226 |
| 546 | 109.2 | 8.749 | 5.248 |
| 547 | 109.4 | 9.129 | 5.223 |
| 548 | 109.6 | 8.865 | 5.424 |
| 549 | 109.8 | 9.026 | 5.361 |
| 550 | 110 | 8.853 | 5.363 |
| 551 | 110.2 | 8.598 | 5.525 |
| 552 | 110.4 | 8.967 | 5.457 |
| 553 | 110.6 | 8.594 | 5.413 |
| 554 | 110.8 | 8.622 | 5.304 |
| 555 | 111 | 8.938 | 5.292 |
| 556 | 111.2 | 8.956 | 5.343 |
| 557 | 111.4 | 8.939 | 5.189 |
| 558 | 111.6 | 8.796 | 5.303 |
| 559 | 111.8 | 8.772 | 5.405 |
| 560 | 112 | 8.859 | 5.165 |
| 561 | 112.2 | 9.012 | 5.089 |
| 562 | 112.4 | 8.963 | 4.984 |
| 563 | 112.6 | 9.179 | 5.409 |
| 564 | 112.8 | 9.412 | 5.226 |
| 565 | 113 | 9.35 | 5.116 |
| 566 | 113.2 | 9.177 | 5.102 |
| 567 | 113.4 | 9.115 | 5.204 |
| 568 | 113.6 | 9.307 | 5.275 |
| 569 | 113.8 | 9.434 | 5.268 |
| 570 | 114 | 9.396 | 5.276 |
| 571 | 114.2 | 9.282 | 5.313 |
| 572 | 114.4 | 9.489 | 5.441 |
| 573 | 114.6 | 8.868 | 5.275 |
| 574 | 114.8 | 8.904 | 5.36 |
| 575 | 115 | 8.871 | 5.384 |
| 576 | 115.2 | 9.042 | 5.045 |
| 577 | 115.4 | 9.1 | 5.203 |
| 578 | 115.6 | 9.37 | 5.316 |
| 579 | 115.8 | 8.993 | 5.129 |
| 580 | 116 | 9.601 | 5.355 |
| 581 | 116.2 | 9.168 | 5.375 |
| 582 | 116.4 | 9.33 | 5.319 |
| 583 | 116.6 | 9.537 | 5.118 |
| 584 | 116.8 | 9.58 | 5.196 |
| 585 | 117 | 9.348 | 4.934 |
| 586 | 117.2 | 8.997 | 5.108 |
| 587 | 117.4 | 9.01 | 5.096 |
| 588 | 117.6 | 8.68 | 5.278 |
| 589 | 117.8 | 9.25 | 5.148 |
| 590 | 118 | 9.718 | 5.211 |
| 591 | 118.2 | 9.394 | 5.386 |
| 592 | 118.4 | 10.102 | 5.186 |
| 593 | 118.6 | 9.427 | 5.429 |
| 594 | 118.8 | 9.34 | 5.381 |
| 595 | 119 | 8.971 | 5.249 |
| 596 | 119.2 | 9.818 | 5.402 |
| 597 | 119.4 | 8.856 | 5.396 |
| 598 | 119.6 | 9.302 | 5.295 |
| 599 | 119.8 | 9.376 | 5.273 |
| 600 | 120 | 8.741 | 5.348 |
| 601 | 120.2 | 8.657 | 5.453 |
| 602 | 120.4 | 8.682 | 5.284 |
| 603 | 120.6 | 8.894 | 5.052 |
| 604 | 120.8 | 9.233 | 5.175 |
| 605 | 121 | 9.001 | 5.241 |
| 606 | 121.2 | 8.97 | 5.277 |
| 607 | 121.4 | 9.201 | 5.158 |
| 608 | 121.6 | 9.299 | 5.162 |
| 609 | 121.8 | 8.992 | 5.057 |
| 610 | 122 | 8.839 | 5.226 |
| 611 | 122.2 | 8.884 | 5.156 |
| 612 | 122.4 | 9.056 | 5.34 |
| 613 | 122.6 | 8.77 | 5.149 |
| 614 | 122.8 | 9.077 | 5.059 |
| 615 | 123 | 9.342 | 5.324 |
| 616 | 123.2 | 9.556 | 5.255 |
| 617 | 123.4 | 9.453 | 5.284 |
| 618 | 123.6 | 9.423 | 5.267 |
| 619 | 123.8 | 9.835 | 5.415 |
| 620 | 124 | 9.759 | 5.315 |
| 621 | 124.2 | 9.638 | 5.295 |
| 622 | 124.4 | 9.525 | 5.342 |
| 623 | 124.6 | 9.988 | 5.364 |
| 624 | 124.8 | 10.035 | 5.19 |
| 625 | 125 | 9.518 | 5.302 |
| 626 | 125.2 | 9.649 | 5.341 |
| 627 | 125.4 | 9.516 | 5.299 |
| 628 | 125.6 | 9.41 | 5.129 |
| 629 | 125.8 | 9.723 | 5.373 |
| 630 | 126 | 10.011 | 5.329 |
| 631 | 126.2 | 9.736 | 5.04 |
| 632 | 126.4 | 9.485 | 5.212 |
| 633 | 126.6 | 9.351 | 5.443 |
| 634 | 126.8 | 9.698 | 5.34 |
| 635 | 127 | 8.953 | 5.218 |
| 636 | 127.2 | 9.526 | 5.323 |
| 637 | 127.4 | 10.201 | 5.251 |
| 638 | 127.6 | 9.859 | 5.149 |
| 639 | 127.8 | 9.9 | 5.412 |
| 640 | 128 | 9.299 | 5.521 |
| 641 | 128.2 | 9.415 | 5.5 |
| 642 | 128.4 | 9.384 | 5.34 |
| 643 | 128.6 | 9.683 | 5.333 |
| 644 | 128.8 | 9.248 | 5.503 |
| 645 | 129 | 9.809 | 5.56 |
| 646 | 129.2 | 9.984 | 5.362 |
| 647 | 129.4 | 9.963 | 5.375 |
| 648 | 129.6 | 9.993 | 5.413 |
| 649 | 129.8 | 9.986 | 5.267 |
| 650 | 130 | 9.513 | 5.588 |
| 651 | 130.2 | 9.795 | 5.518 |
| 652 | 130.4 | 9.184 | 5.357 |
| 653 | 130.6 | 9.507 | 5.526 |
| 654 | 130.8 | 9.186 | 5.236 |
| 655 | 131 | 9.453 | 5.373 |
| 656 | 131.2 | 9.684 | 5.426 |
| 657 | 131.4 | 9.629 | 5.527 |
| 658 | 131.6 | 9.4 | 5.468 |
| 659 | 131.8 | 9.37 | 5.314 |
| 660 | 132 | 9.498 | 5.719 |
| 661 | 132.2 | 9.891 | 5.307 |
| 662 | 132.4 | 9.557 | 5.525 |
| 663 | 132.6 | 9.608 | 5.348 |
| 664 | 132.8 | 9.454 | 5.414 |
| 665 | 133 | 9.27 | 5.405 |
| 666 | 133.2 | 9.756 | 5.352 |
| 667 | 133.4 | 9.843 | 5.405 |
| 668 | 133.6 | 9.347 | 5.218 |
| 669 | 133.8 | 9.404 | 5.318 |
| 670 | 134 | 10.122 | 5.478 |
| 671 | 134.2 | 9.98 | 5.35 |
| 672 | 134.4 | 9.599 | 5.401 |
| 673 | 134.6 | 9.806 | 5.51 |
| 674 | 134.8 | 9.716 | 5.499 |
| 675 | 135 | 9.92 | 5.289 |
| 676 | 135.2 | 9.776 | 5.643 |
| 677 | 135.4 | 9.658 | 5.642 |
| 678 | 135.6 | 9.382 | 5.45 |
| 679 | 135.8 | 9.565 | 5.277 |
| 680 | 136 | 9.682 | 5.296 |
| 681 | 136.2 | 9.342 | 5.236 |
| 682 | 136.4 | 9.396 | 5.109 |
| 683 | 136.6 | 8.948 | 5.314 |
| 684 | 136.8 | 9.13 | 5.356 |
| 685 | 137 | 9.042 | 5.377 |
| 686 | 137.2 | 9.416 | 5.284 |
| 687 | 137.4 | 9.161 | 5.425 |
| 688 | 137.6 | 9.111 | 5.193 |
| 689 | 137.8 | 9.207 | 5.395 |
| 690 | 138 | 9.064 | 5.145 |
| 691 | 138.2 | 9.355 | 5.461 |
| 692 | 138.4 | 8.745 | 5.36 |
| 693 | 138.6 | 8.623 | 5.204 |
| 694 | 138.8 | 8.545 | 5.478 |
| 695 | 139 | 8.765 | 5.567 |
| 696 | 139.2 | 8.528 | 5.446 |
| 697 | 139.4 | 8.859 | 5.289 |
| 698 | 139.6 | 8.959 | 5.319 |
| 699 | 139.8 | 9.367 | 5.453 |
| 700 | 140 | 9.46 | 5.344 |
| 701 | 140.2 | 9.399 | 5.441 |
| 702 | 140.4 | 8.961 | 5.443 |
| 703 | 140.6 | 8.799 | 5.543 |
| 704 | 140.8 | 8.816 | 5.566 |
| 705 | 141 | 8.802 | 5.433 |
| 706 | 141.2 | 8.774 | 5.364 |
| 707 | 141.4 | 9.133 | 5.465 |
| 708 | 141.6 | 9.245 | 5.459 |
| 709 | 141.8 | 9.254 | 5.178 |
| 710 | 142 | 9.389 | 5.343 |
| 711 | 142.2 | 9.012 | 5.154 |
| 712 | 142.4 | 9.11 | 5.159 |
| 713 | 142.6 | 9.304 | 5.407 |
| 714 | 142.8 | 9.28 | 5.426 |
| 715 | 143 | 9.179 | 5.514 |
| 716 | 143.2 | 9.302 | 5.439 |
| 717 | 143.4 | 9.554 | 5.494 |
| 718 | 143.6 | 9.26 | 5.435 |
| 719 | 143.8 | 9.261 | 5.363 |
| 720 | 144 | 9.249 | 5.293 |
| 721 | 144.2 | 9.298 | 5.593 |
| 722 | 144.4 | 9.263 | 5.4 |
| 723 | 144.6 | 9.023 | 5.303 |
| 724 | 144.8 | 9.701 | 5.283 |
| 725 | 145 | 9.115 | 5.29 |
| 726 | 145.2 | 9.279 | 5.365 |
| 727 | 145.4 | 9.332 | 5.446 |
| 728 | 145.6 | 9.073 | 5.362 |
| 729 | 145.8 | 9.261 | 5.349 |
| 730 | 146 | 9.057 | 5.564 |
| 731 | 146.2 | 9.548 | 5.423 |
| 732 | 146.4 | 9.213 | 5.453 |
| 733 | 146.6 | 9.422 | 5.631 |
| 734 | 146.8 | 9.383 | 5.712 |
| 735 | 147 | 9.242 | 5.508 |
| 736 | 147.2 | 9.328 | 5.358 |
| 737 | 147.4 | 8.886 | 5.359 |
| 738 | 147.6 | 9.474 | 5.253 |
| 739 | 147.8 | 8.863 | 5.451 |
| 740 | 148 | 8.844 | 5.681 |
| 741 | 148.2 | 9.182 | 5.46 |
| 742 | 148.4 | 9.558 | 5.543 |
| 743 | 148.6 | 9.299 | 5.61 |
| 744 | 148.8 | 9.541 | 5.549 |
| 745 | 149 | 9.463 | 5.328 |
| 746 | 149.2 | 9.533 | 5.387 |
| 747 | 149.4 | 9.399 | 5.479 |
| 748 | 149.6 | 9.186 | 5.593 |
| 749 | 149.8 | 8.968 | 5.409 |
| 750 | 150 | 9.618 | 5.565 |
| 751 | 150.2 | 9.798 | 5.616 |
| 752 | 150.4 | 9.28 | 5.794 |
| 753 | 150.6 | 9.287 | 5.69 |
| 754 | 150.8 | 8.968 | 5.433 |
| 755 | 151 | 9.121 | 5.385 |
| 756 | 151.2 | 9.279 | 5.77 |
| 757 | 151.4 | 8.963 | 5.632 |
| 758 | 151.6 | 9.207 | 5.419 |
| 759 | 151.8 | 9.17 | 5.571 |
| 760 | 152 | 9.032 | 5.575 |
| 761 | 152.2 | 9.143 | 5.382 |
| 762 | 152.4 | 9.297 | 5.335 |
| 763 | 152.6 | 9.229 | 5.655 |
| 764 | 152.8 | 9.191 | 5.304 |
| 765 | 153 | 8.928 | 5.32 |
| 766 | 153.2 | 9.065 | 5.455 |
| 767 | 153.4 | 9.277 | 5.506 |
| 768 | 153.6 | 9.182 | 5.352 |
| 769 | 153.8 | 9.186 | 5.587 |
| 770 | 154 | 9.766 | 5.105 |
| 771 | 154.2 | 9.625 | 5.349 |
| 772 | 154.4 | 9.605 | 5.449 |
| 773 | 154.6 | 9.123 | 5.378 |
| 774 | 154.8 | 9.088 | 5.276 |
| 775 | 155 | 9.244 | 5.256 |
| 776 | 155.2 | 8.977 | 5.443 |
| 777 | 155.4 | 9.462 | 5.339 |
| 778 | 155.6 | 9.348 | 5.334 |
| 779 | 155.8 | 9.226 | 5.397 |
| 780 | 156 | 9.271 | 5.283 |
| 781 | 156.2 | 9.398 | 5.481 |
| 782 | 156.4 | 9.844 | 5.4 |
| 783 | 156.6 | 9.361 | 5.38 |
| 784 | 156.8 | 9.534 | 5.336 |
| 785 | 157 | 9.416 | 5.294 |
| 786 | 157.2 | 9.424 | 5.42 |
| 787 | 157.4 | 9.176 | 5.589 |
| 788 | 157.6 | 9.361 | 5.498 |
| 789 | 157.8 | 9.143 | 5.362 |
| 790 | 158 | 9.113 | 5.606 |
| 791 | 158.2 | 9.101 | 5.604 |
| 792 | 158.4 | 9.283 | 5.564 |
| 793 | 158.6 | 9.521 | 5.535 |
| 794 | 158.8 | 9.265 | 5.409 |
| 795 | 159 | 9.255 | 5.446 |
| 796 | 159.2 | 9.486 | 5.381 |
| 797 | 159.4 | 9.183 | 5.679 |
| 798 | 159.6 | 9.503 | 5.699 |
| 799 | 159.8 | 9.05 | 5.596 |
| 800 | 160 | 9.525 | 5.509 |
| 801 | 160.2 | 9.666 | 5.476 |
| 802 | 160.4 | 9.451 | 5.523 |
| 803 | 160.6 | 9.451 | 5.316 |
| 804 | 160.8 | 9.42 | 5.741 |
| 805 | 161 | 9.048 | 5.602 |
| 806 | 161.2 | 9.271 | 5.682 |
| 807 | 161.4 | 9.075 | 5.437 |
| 808 | 161.6 | 9.751 | 5.638 |
| 809 | 161.8 | 9.314 | 5.69 |
| 810 | 162 | 9.662 | 5.652 |
| 811 | 162.2 | 9.596 | 5.487 |
| 812 | 162.4 | 9.917 | 5.37 |
| 813 | 162.6 | 9.895 | 5.353 |
| 814 | 162.8 | 9.798 | 5.49 |
| 815 | 163 | 9.743 | 5.662 |
| 816 | 163.2 | 9.761 | 5.531 |
| 817 | 163.4 | 9.737 | 5.739 |
| 818 | 163.6 | 9.841 | 5.557 |
| 819 | 163.8 | 9.702 | 5.612 |
| 820 | 164 | 9.927 | 5.461 |
| 821 | 164.2 | 10.068 | 5.533 |
| 822 | 164.4 | 10.06 | 5.551 |
| 823 | 164.6 | 9.96 | 5.697 |
| 824 | 164.8 | 9.636 | 5.446 |
| 825 | 165 | 9.741 | 5.491 |
| 826 | 165.2 | 9.826 | 5.636 |
| 827 | 165.4 | 9.625 | 5.552 |
| 828 | 165.6 | 9.853 | 5.533 |
| 829 | 165.8 | 9.436 | 5.731 |
| 830 | 166 | 9.84 | 5.423 |
| 831 | 166.2 | 9.91 | 5.302 |
| 832 | 166.4 | 9.923 | 5.322 |
| 833 | 166.6 | 9.949 | 5.424 |
| 834 | 166.8 | 9.642 | 5.555 |
| 835 | 167 | 10.005 | 5.306 |
| 836 | 167.2 | 10.008 | 5.426 |
| 837 | 167.4 | 9.906 | 5.457 |
| 838 | 167.6 | 9.982 | 5.352 |
| 839 | 167.8 | 9.842 | 5.39 |
| 840 | 168 | 10.104 | 5.505 |
| 841 | 168.2 | 10.267 | 5.472 |
| 842 | 168.4 | 10.509 | 5.405 |
| 843 | 168.6 | 9.948 | 5.527 |
| 844 | 168.8 | 10.252 | 5.542 |
| 845 | 169 | 10.404 | 5.43 |
| 846 | 169.2 | 10.423 | 5.243 |
| 847 | 169.4 | 10.43 | 5.315 |
| 848 | 169.6 | 10.657 | 5.287 |
| 849 | 169.8 | 10.654 | 5.393 |
| 850 | 170 | 10.519 | 5.176 |
| 851 | 170.2 | 10.66 | 5.29 |
| 852 | 170.4 | 10.323 | 5.219 |
| 853 | 170.6 | 10.299 | 5.505 |
| 854 | 170.8 | 10.21 | 5.52 |
| 855 | 171 | 10.169 | 5.524 |
| 856 | 171.2 | 10.175 | 5.357 |
| 857 | 171.4 | 10.159 | 5.235 |
| 858 | 171.6 | 10.26 | 5.161 |
| 859 | 171.8 | 10.068 | 5.124 |
| 860 | 172 | 10.716 | 5.097 |
| 861 | 172.2 | 10.418 | 5.231 |
| 862 | 172.4 | 9.991 | 5.029 |
| 863 | 172.6 | 10.186 | 5.314 |
| 864 | 172.8 | 10.413 | 5.169 |
| 865 | 173 | 10.106 | 5.17 |
| 866 | 173.2 | 9.999 | 4.976 |
| 867 | 173.4 | 10.14 | 5.162 |
| 868 | 173.6 | 9.988 | 5.135 |
| 869 | 173.8 | 9.988 | 5.245 |
| 870 | 174 | 10.185 | 5.283 |
| 871 | 174.2 | 9.566 | 5.153 |
| 872 | 174.4 | 10.089 | 5.351 |
| 873 | 174.6 | 10.087 | 5.299 |
| 874 | 174.8 | 10.012 | 5.448 |
| 875 | 175 | 9.72 | 5.42 |
| 876 | 175.2 | 9.992 | 5.501 |
| 877 | 175.4 | 10.657 | 5.616 |
| 878 | 175.6 | 10.319 | 5.504 |
| 879 | 175.8 | 9.808 | 5.436 |
| 880 | 176 | 10.085 | 5.36 |
| 881 | 176.2 | 10.011 | 5.356 |
| 882 | 176.4 | 10.261 | 5.574 |
| 883 | 176.6 | 9.877 | 5.559 |
| 884 | 176.8 | 10.377 | 5.246 |
| 885 | 177 | 9.938 | 5.672 |
| 886 | 177.2 | 9.927 | 5.781 |
| 887 | 177.4 | 9.729 | 5.44 |
| 888 | 177.6 | 9.626 | 5.595 |
| 889 | 177.8 | 9.716 | 5.823 |
| 890 | 178 | 9.736 | 5.693 |
| 891 | 178.2 | 10.039 | 5.821 |
| 892 | 178.4 | 10.23 | 5.45 |
| 893 | 178.6 | 10.004 | 5.625 |
| 894 | 178.8 | 9.905 | 5.397 |
| 895 | 179 | 10.043 | 5.219 |
| 896 | 179.2 | 10.029 | 5.471 |
| 897 | 179.4 | 9.94 | 5.749 |
| 898 | 179.6 | 9.658 | 5.633 |
| 899 | 179.8 | 9.469 | 5.652 |
| 900 | 180 | 9.324 | 5.394 |
| 901 | 180.2 | 9.639 | 5.294 |
| 902 | 180.4 | 9.889 | 5.562 |
| 903 | 180.6 | 10.301 | 5.609 |
| 904 | 180.8 | 9.987 | 5.411 |
| 905 | 181 | 10.096 | 5.496 |
| 906 | 181.2 | 10.065 | 5.508 |
| 907 | 181.4 | 9.884 | 5.63 |
| 908 | 181.6 | 9.935 | 5.331 |
| 909 | 181.8 | 9.827 | 5.323 |
| 910 | 182 | 9.543 | 5.527 |
| 911 | 182.2 | 9.56 | 5.408 |
| 912 | 182.4 | 9.51 | 5.483 |
| 913 | 182.6 | 9.491 | 5.142 |
| 914 | 182.8 | 9.62 | 5.378 |
| 915 | 183 | 9.503 | 5.187 |
| 916 | 183.2 | 9.671 | 5.079 |
| 917 | 183.4 | 9.553 | 5.136 |
| 918 | 183.6 | 9.445 | 5.216 |
| 919 | 183.8 | 9.708 | 5.3 |
| 920 | 184 | 9.229 | 5.483 |
| 921 | 184.2 | 9.576 | 5.357 |
| 922 | 184.4 | 9.467 | 5.276 |
| 923 | 184.6 | 9.851 | 5.136 |
| 924 | 184.8 | 9.946 | 5.193 |
| 925 | 185 | 9.814 | 5.475 |
| 926 | 185.2 | 9.73 | 5.351 |
| 927 | 185.4 | 9.819 | 5.099 |
| 928 | 185.6 | 9.733 | 5.433 |
| 929 | 185.8 | 9.795 | 5.418 |
| 930 | 186 | 9.554 | 5.339 |
| 931 | 186.2 | 9.835 | 5.581 |
| 932 | 186.4 | 9.996 | 5.67 |
| 933 | 186.6 | 9.784 | 5.547 |
| 934 | 186.8 | 9.566 | 5.534 |
| 935 | 187 | 9.855 | 5.825 |
| 936 | 187.2 | 10.305 | 5.54 |
| 937 | 187.4 | 10.069 | 5.725 |
| 938 | 187.6 | 9.642 | 5.448 |
| 939 | 187.8 | 9.596 | 5.491 |
| 940 | 188 | 9.701 | 5.726 |
| 941 | 188.2 | 9.439 | 5.502 |
| 942 | 188.4 | 9.33 | 5.46 |
| 943 | 188.6 | 9.669 | 5.728 |
| 944 | 188.8 | 9.33 | 6.035 |
| 945 | 189 | 9.744 | 5.975 |
| 946 | 189.2 | 9.452 | 5.88 |
| 947 | 189.4 | 9.346 | 5.742 |
| 948 | 189.6 | 9.572 | 5.611 |
| 949 | 189.8 | 9.823 | 5.688 |
| 950 | 190 | 9.663 | 5.804 |
| 951 | 190.2 | 9.71 | 5.614 |
| 952 | 190.4 | 9.83 | 5.796 |
| 953 | 190.6 | 9.947 | 6.134 |
| 954 | 190.8 | 9.662 | 6.023 |
| 955 | 191 | 9.6 | 5.951 |
| 956 | 191.2 | 9.663 | 5.728 |
| 957 | 191.4 | 10.033 | 5.499 |
| 958 | 191.6 | 9.962 | 5.665 |
| 959 | 191.8 | 9.802 | 5.543 |
| 960 | 192 | 10.308 | 5.768 |
| 961 | 192.2 | 10.179 | 5.608 |
| 962 | 192.4 | 10.343 | 5.928 |
| 963 | 192.6 | 10.119 | 5.823 |
| 964 | 192.8 | 10.078 | 5.918 |
| 965 | 193 | 9.728 | 5.859 |
| 966 | 193.2 | 10.318 | 5.899 |
| 967 | 193.4 | 10.05 | 5.848 |
| 968 | 193.6 | 10.276 | 5.67 |
| 969 | 193.8 | 10.282 | 5.78 |
| 970 | 194 | 10.01 | 5.373 |
| 971 | 194.2 | 10.2 | 5.518 |
| 972 | 194.4 | 9.817 | 5.196 |
| 973 | 194.6 | 10.164 | 5.459 |
| 974 | 194.8 | 10.393 | 5.167 |
| 975 | 195 | 9.767 | 5.367 |
| 976 | 195.2 | 10.033 | 5.419 |
| 977 | 195.4 | 9.696 | 5.36 |
| 978 | 195.6 | 9.757 | 5.563 |
| 979 | 195.8 | 10.021 | 5.644 |
| 980 | 196 | 9.934 | 5.439 |
| 981 | 196.2 | 9.83 | 5.625 |
| 982 | 196.4 | 10.175 | 5.438 |
| 983 | 196.6 | 9.896 | 5.38 |
| 984 | 196.8 | 9.623 | 5.488 |
| 985 | 197 | 9.635 | 5.283 |
| 986 | 197.2 | 9.771 | 5.19 |
| 987 | 197.4 | 9.806 | 5.355 |
| 988 | 197.6 | 9.566 | 5.288 |
| 989 | 197.8 | 9.591 | 5.239 |
| 990 | 198 | 9.806 | 5.403 |
| 991 | 198.2 | 9.757 | 5.205 |
| 992 | 198.4 | 9.454 | 5.202 |
| 993 | 198.6 | 9.481 | 5.204 |
| 994 | 198.8 | 10.156 | 5.351 |
| 995 | 199 | 9.664 | 5.454 |
| 996 | 199.2 | 10.286 | 5.313 |
| 997 | 199.4 | 9.79 | 5.264 |
| 998 | 199.6 | 10.2 | 5.536 |
| 999 | 199.8 | 10.146 | 5.2 |
| 1000 | 200 | 10.4 | 5.188 |
| 1001 | 200.2 | 10.457 | 5.134 |
