# Supplementary material for: Immunoinformatics and reverse vaccinology design of self-amplifying RNA vaccine against marburg virus with structural dynamics and immune simulation analysis
Source: PLoS One. 2026 Oct 23;21(10):e0361136. doi: 10.1371/journal.pone.0361136 (PMC13693908; doi:10.1371/journal.pone.0361136)
Supplement: Supplementary file 4 [file pone.0361136.s003.docx]

**Protein RMSF**

| **Residue** | **MB-mVax-TLR2** | **MB-mVax-TLR4** |
| --- | --- | --- |
| 0 | 6.176 | 3.576 |
| 1 | 4.86 | 3.075 |
| 2 | 4.962 | 2.844 |
| 3 | 3.901 | 3.004 |
| 4 | 4.402 | 3.088 |
| 5 | 4.35 | 3.098 |
| 6 | 4.021 | 3.345 |
| 7 | 3.502 | 3.314 |
| 8 | 3.263 | 3.306 |
| 9 | 3.222 | 3.132 |
| 10 | 3.116 | 2.884 |
| 11 | 3.553 | 2.744 |
| 12 | 4.595 | 2.592 |
| 13 | 5.794 | 2.552 |
| 14 | 6.152 | 2.39 |
| 15 | 6.017 | 2.471 |
| 16 | 5.034 | 2.724 |
| 17 | 4.771 | 2.745 |
| 18 | 3.994 | 2.598 |
| 19 | 3.527 | 2.564 |
| 20 | 4.427 | 2.528 |
| 21 | 4.701 | 2.457 |
| 22 | 5.287 | 2.748 |
| 23 | 4.701 | 3.035 |
| 24 | 4.453 | 3.216 |
| 25 | 3.801 | 2.948 |
| 26 | 3.711 | 3.105 |
| 27 | 3.316 | 3.02 |
| 28 | 3 | 3.074 |
| 29 | 2.791 | 2.794 |
| 30 | 2.753 | 2.717 |
| 31 | 2.747 | 2.486 |
| 32 | 2.833 | 2.37 |
| 33 | 2.649 | 2.223 |
| 34 | 2.722 | 2.147 |
| 35 | 3.021 | 2.053 |
| 36 | 3.129 | 2.183 |
| 37 | 3.033 | 2.254 |
| 38 | 3.064 | 2.375 |
| 39 | 3.102 | 2.166 |
| 40 | 3.052 | 2.146 |
| 41 | 3.391 | 2.087 |
| 42 | 3.598 | 2.043 |
| 43 | 3.705 | 2.184 |
| 44 | 3.363 | 2.285 |
| 45 | 3.38 | 2.356 |
| 46 | 3.748 | 2.198 |
| 47 | 3.862 | 2.223 |
| 48 | 3.361 | 2.597 |
| 49 | 3.442 | 3.094 |
| 50 | 3.172 | 2.861 |
| 51 | 2.749 | 3.074 |
| 52 | 2.538 | 2.945 |
| 53 | 2.377 | 2.548 |
| 54 | 2.347 | 2.455 |
| 55 | 2.303 | 2.113 |
| 56 | 2.369 | 2.003 |
| 57 | 2.257 | 1.88 |
| 58 | 2.403 | 1.807 |
| 59 | 2.636 | 1.801 |
| 60 | 2.69 | 1.968 |
| 61 | 2.733 | 2.035 |
| 62 | 2.888 | 2.073 |
| 63 | 2.946 | 1.944 |
| 64 | 2.976 | 2.062 |
| 65 | 3.2 | 2.067 |
| 66 | 3.304 | 2.006 |
| 67 | 3.319 | 2.212 |
| 68 | 3.297 | 2.326 |
| 69 | 2.91 | 2.349 |
| 70 | 3.241 | 2.16 |
| 71 | 3.344 | 2.103 |
| 72 | 2.963 | 2.538 |
| 73 | 3.311 | 2.851 |
| 74 | 2.689 | 2.39 |
| 75 | 2.322 | 2.634 |
| 76 | 2.177 | 2.693 |
| 77 | 2.013 | 2.249 |
| 78 | 1.992 | 2.12 |
| 79 | 1.951 | 1.758 |
| 80 | 2.061 | 1.709 |
| 81 | 1.961 | 1.656 |
| 82 | 2.137 | 1.614 |
| 83 | 2.29 | 1.553 |
| 84 | 2.426 | 1.711 |
| 85 | 2.551 | 1.71 |
| 86 | 2.926 | 1.837 |
| 87 | 3.088 | 1.87 |
| 88 | 2.698 | 2.36 |
| 89 | 2.732 | 2.298 |
| 90 | 2.765 | 2.251 |
| 91 | 3.04 | 2.409 |
| 92 | 2.826 | 2.642 |
| 93 | 2.686 | 2.381 |
| 94 | 3.023 | 2.215 |
| 95 | 2.796 | 2.288 |
| 96 | 2.353 | 2.529 |
| 97 | 2.336 | 2.567 |
| 98 | 2.163 | 2.284 |
| 99 | 1.966 | 2.672 |
| 100 | 2.073 | 2.46 |
| 101 | 1.787 | 1.933 |
| 102 | 1.699 | 1.79 |
| 103 | 1.706 | 1.45 |
| 104 | 1.807 | 1.404 |
| 105 | 1.807 | 1.388 |
| 106 | 1.927 | 1.381 |
| 107 | 1.989 | 1.366 |
| 108 | 2.102 | 1.496 |
| 109 | 1.915 | 1.477 |
| 110 | 1.927 | 1.566 |
| 111 | 1.83 | 1.468 |
| 112 | 1.816 | 1.514 |
| 113 | 2.009 | 1.574 |
| 114 | 2.199 | 1.505 |
| 115 | 2.306 | 1.722 |
| 116 | 2.44 | 1.985 |
| 117 | 2.207 | 1.874 |
| 118 | 2.548 | 1.978 |
| 119 | 2.878 | 2.155 |
| 120 | 2.65 | 2.528 |
| 121 | 2.304 | 2.322 |
| 122 | 2.567 | 2.164 |
| 123 | 2.296 | 2.448 |
| 124 | 1.988 | 2.249 |
| 125 | 1.904 | 1.653 |
| 126 | 1.621 | 1.572 |
| 127 | 1.541 | 1.312 |
| 128 | 1.557 | 1.232 |
| 129 | 1.623 | 1.219 |
| 130 | 1.736 | 1.247 |
| 131 | 1.864 | 1.235 |
| 132 | 2.104 | 1.292 |
| 133 | 2.619 | 1.294 |
| 134 | 2.143 | 1.393 |
| 135 | 1.882 | 1.318 |
| 136 | 2.076 | 1.379 |
| 137 | 2.006 | 1.394 |
| 138 | 1.842 | 1.561 |
| 139 | 2.079 | 1.51 |
| 140 | 1.994 | 1.546 |
| 141 | 2.108 | 1.67 |
| 142 | 2.17 | 1.775 |
| 143 | 2.453 | 1.824 |
| 144 | 2.852 | 1.71 |
| 145 | 2.336 | 1.877 |
| 146 | 1.873 | 2.034 |
| 147 | 2.593 | 1.862 |
| 148 | 1.937 | 2.024 |
| 149 | 1.591 | 1.753 |
| 150 | 1.532 | 1.377 |
| 151 | 1.378 | 1.303 |
| 152 | 1.362 | 1.16 |
| 153 | 1.357 | 1.076 |
| 154 | 1.444 | 1.099 |
| 155 | 1.73 | 1.106 |
| 156 | 2.035 | 1.097 |
| 157 | 1.82 | 1.214 |
| 158 | 1.982 | 1.213 |
| 159 | 1.847 | 1.307 |
| 160 | 2.168 | 1.243 |
| 161 | 2.017 | 1.307 |
| 162 | 1.881 | 1.353 |
| 163 | 1.964 | 1.349 |
| 164 | 2.097 | 1.509 |
| 165 | 1.82 | 1.631 |
| 166 | 1.836 | 1.827 |
| 167 | 1.615 | 1.683 |
| 168 | 1.625 | 1.552 |
| 169 | 1.783 | 1.677 |
| 170 | 1.711 | 1.69 |
| 171 | 2.039 | 1.584 |
| 172 | 1.724 | 1.712 |
| 173 | 1.539 | 1.879 |
| 174 | 1.441 | 1.82 |
| 175 | 1.297 | 2.062 |
| 176 | 1.28 | 2.133 |
| 177 | 1.281 | 1.568 |
| 178 | 1.33 | 1.552 |
| 179 | 1.402 | 1.179 |
| 180 | 1.503 | 1.114 |
| 181 | 1.599 | 0.985 |
| 182 | 1.701 | 0.975 |
| 183 | 1.636 | 1.018 |
| 184 | 1.866 | 1.012 |
| 185 | 1.708 | 1.085 |
| 186 | 1.48 | 1.12 |
| 187 | 1.525 | 1.242 |
| 188 | 1.659 | 1.242 |
| 189 | 1.533 | 1.39 |
| 190 | 1.398 | 1.396 |
| 191 | 1.62 | 1.313 |
| 192 | 1.696 | 1.451 |
| 193 | 1.538 | 1.485 |
| 194 | 1.513 | 1.676 |
| 195 | 1.685 | 1.622 |
| 196 | 1.657 | 1.443 |
| 197 | 1.429 | 1.702 |
| 198 | 1.358 | 1.799 |
| 199 | 1.256 | 1.642 |
| 200 | 1.169 | 1.564 |
| 201 | 1.153 | 1.268 |
| 202 | 1.235 | 1.205 |
| 203 | 1.272 | 1.119 |
| 204 | 1.365 | 0.922 |
| 205 | 1.389 | 0.902 |
| 206 | 1.542 | 0.927 |
| 207 | 1.483 | 0.963 |
| 208 | 1.649 | 0.992 |
| 209 | 1.831 | 1.008 |
| 210 | 1.756 | 1.113 |
| 211 | 1.952 | 1.38 |
| 212 | 2.04 | 1.289 |
| 213 | 2.711 | 1.249 |
| 214 | 2.456 | 1.47 |
| 215 | 2.423 | 1.344 |
| 216 | 4.033 | 1.104 |
| 217 | 3.654 | 1.215 |
| 218 | 3.341 | 1.373 |
| 219 | 3.318 | 1.218 |
| 220 | 2.737 | 1.059 |
| 221 | 1.841 | 1.175 |
| 222 | 1.524 | 1.243 |
| 223 | 1.433 | 1.134 |
| 224 | 1.252 | 1.318 |
| 225 | 1.232 | 1.429 |
| 226 | 1.063 | 1.181 |
| 227 | 1.026 | 1.27 |
| 228 | 0.962 | 1.128 |
| 229 | 1.064 | 1.071 |
| 230 | 1.073 | 1.001 |
| 231 | 1.198 | 0.82 |
| 232 | 1.269 | 0.796 |
| 233 | 1.37 | 0.807 |
| 234 | 1.404 | 0.896 |
| 235 | 1.43 | 0.916 |
| 236 | 1.378 | 1.017 |
| 237 | 1.65 | 1.09 |
| 238 | 1.627 | 1.163 |
| 239 | 1.54 | 1.167 |
| 240 | 1.622 | 1.193 |
| 241 | 1.68 | 1.078 |
| 242 | 1.405 | 1.1 |
| 243 | 1.364 | 1.319 |
| 244 | 1.494 | 1.293 |
| 245 | 1.402 | 1.164 |
| 246 | 1.277 | 1.147 |
| 247 | 1.394 | 1.102 |
| 248 | 1.436 | 1.255 |
| 249 | 1.327 | 1.277 |
| 250 | 1.49 | 1.117 |
| 251 | 2.204 | 1.209 |
| 252 | 1.268 | 1.177 |
| 253 | 1.123 | 1.072 |
| 254 | 1.019 | 1.173 |
| 255 | 0.903 | 1.364 |
| 256 | 0.837 | 1.268 |
| 257 | 0.898 | 1.262 |
| 258 | 0.983 | 1.076 |
| 259 | 1.022 | 1.07 |
| 260 | 1.16 | 1.011 |
| 261 | 1.265 | 0.898 |
| 262 | 1.274 | 0.753 |
| 263 | 1.45 | 0.744 |
| 264 | 1.433 | 0.821 |
| 265 | 1.312 | 0.85 |
| 266 | 1.293 | 0.914 |
| 267 | 1.499 | 1.025 |
| 268 | 1.674 | 1.077 |
| 269 | 1.799 | 1.086 |
| 270 | 1.923 | 1.125 |
| 271 | 1.842 | 1.284 |
| 272 | 2.321 | 1.281 |
| 273 | 2.531 | 1.208 |
| 274 | 2.609 | 1.285 |
| 275 | 2.184 | 1.287 |
| 276 | 1.855 | 1.092 |
| 277 | 1.802 | 1.036 |
| 278 | 1.65 | 1.152 |
| 279 | 1.654 | 1.22 |
| 280 | 1.813 | 1.082 |
| 281 | 1.81 | 1.198 |
| 282 | 1.581 | 1.33 |
| 283 | 1.689 | 1.222 |
| 284 | 1.28 | 1.307 |
| 285 | 0.881 | 1.059 |
| 286 | 0.77 | 0.856 |
| 287 | 0.774 | 0.775 |
| 288 | 0.784 | 0.791 |
| 289 | 0.932 | 0.781 |
| 290 | 1.008 | 0.909 |
| 291 | 1.153 | 0.965 |
| 292 | 1.21 | 1.049 |
| 293 | 1.428 | 1.061 |
| 294 | 1.273 | 1.123 |
| 295 | 1.179 | 1.258 |
| 296 | 1.253 | 1.161 |
| 297 | 1.233 | 1.288 |
| 298 | 1.186 | 1.41 |
| 299 | 1.174 | 1.501 |
| 300 | 1.288 | 1.842 |
| 301 | 1.251 | 1.459 |
| 302 | 1.539 | 1.383 |
| 303 | 2.198 | 1.118 |
| 304 | 2.136 | 1.151 |
| 305 | 2.041 | 1.033 |
| 306 | 2.121 | 1.044 |
| 307 | 2.085 | 0.96 |
| 308 | 1.943 | 0.887 |
| 309 | 1.991 | 0.86 |
| 310 | 1.309 | 0.965 |
| 311 | 1.048 | 0.969 |
| 312 | 1.078 | 0.885 |
| 313 | 0.873 | 0.872 |
| 314 | 0.846 | 0.959 |
| 315 | 0.819 | 0.999 |
| 316 | 0.828 | 1.116 |
| 317 | 0.816 | 1.184 |
| 318 | 0.881 | 1.079 |
| 319 | 0.947 | 1.122 |
| 320 | 1.029 | 1.158 |
| 321 | 1.02 | 1.057 |
| 322 | 1.074 | 1.158 |
| 323 | 1.213 | 1.066 |
| 324 | 1.124 | 1.132 |
| 325 | 1.193 | 1.121 |
| 326 | 1.17 | 0.983 |
| 327 | 1.324 | 1.014 |
| 328 | 1.208 | 0.949 |
| 329 | 1.063 | 0.951 |
| 330 | 1.212 | 0.887 |
| 331 | 1.334 | 0.877 |
| 332 | 1.198 | 0.89 |
| 333 | 1.328 | 0.897 |
| 334 | 1.373 | 0.85 |
| 335 | 1.129 | 0.937 |
| 336 | 1.084 | 1.083 |
| 337 | 0.973 | 1.077 |
| 338 | 0.897 | 1.136 |
| 339 | 0.857 | 1.251 |
| 340 | 0.847 | 1.081 |
| 341 | 0.836 | 1.154 |
| 342 | 0.875 | 1.097 |
| 343 | 0.907 | 1.07 |
| 344 | 0.983 | 1.236 |
| 345 | 0.996 | 1.092 |
| 346 | 0.985 | 1.2 |
| 347 | 0.996 | 1.169 |
| 348 | 1.101 | 1.045 |
| 349 | 1.042 | 1.089 |
| 350 | 0.986 | 0.981 |
| 351 | 1.099 | 0.953 |
| 352 | 1.14 | 0.921 |
| 353 | 1.075 | 0.889 |
| 354 | 1.116 | 0.928 |
| 355 | 1.269 | 0.962 |
| 356 | 1.394 | 0.967 |
| 357 | 1.385 | 0.964 |
| 358 | 1.142 | 0.994 |
| 359 | 1.122 | 1.014 |
| 360 | 1.369 | 1.012 |
| 361 | 1.232 | 1.188 |
| 362 | 1.109 | 1.174 |
| 363 | 1.125 | 1.058 |
| 364 | 1.033 | 1.128 |
| 365 | 0.973 | 1.257 |
| 366 | 0.927 | 1.272 |
| 367 | 0.874 | 1.462 |
| 368 | 0.897 | 1.57 |
| 369 | 0.951 | 1.534 |
| 370 | 0.923 | 1.628 |
| 371 | 1.007 | 1.505 |
| 372 | 1.002 | 1.411 |
| 373 | 1.169 | 1.448 |
| 374 | 1.17 | 1.192 |
| 375 | 1.175 | 1.207 |
| 376 | 1.227 | 1.033 |
| 377 | 1.132 | 0.949 |
| 378 | 1.029 | 0.904 |
| 379 | 1.129 | 0.859 |
| 380 | 1.192 | 0.802 |
| 381 | 1.166 | 0.87 |
| 382 | 1.199 | 0.913 |
| 383 | 1.26 | 0.98 |
| 384 | 1.45 | 1.008 |
| 385 | 1.38 | 0.975 |
| 386 | 1.561 | 1.062 |
| 387 | 1.425 | 1.1 |
| 388 | 1.242 | 1.191 |
| 389 | 1.27 | 1.224 |
| 390 | 1.141 | 1.207 |
| 391 | 1.031 | 1.229 |
| 392 | 0.96 | 1.412 |
| 393 | 0.946 | 1.374 |
| 394 | 1.025 | 1.275 |
| 395 | 1.054 | 1.368 |
| 396 | 1.03 | 1.284 |
| 397 | 1.201 | 1.073 |
| 398 | 1.214 | 1.139 |
| 399 | 1.541 | 1.011 |
| 400 | 1.585 | 0.919 |
| 401 | 1.596 | 0.813 |
| 402 | 1.675 | 0.795 |
| 403 | 1.784 | 0.803 |
| 404 | 1.579 | 0.787 |
| 405 | 1.373 | 0.798 |
| 406 | 1.349 | 0.96 |
| 407 | 1.236 | 0.904 |
| 408 | 1.355 | 1.022 |
| 409 | 1.598 | 1.104 |
| 410 | 1.442 | 1.122 |
| 411 | 1.263 | 1.442 |
| 412 | 1.324 | 1.362 |
| 413 | 1.21 | 1.381 |
| 414 | 1.139 | 1.387 |
| 415 | 1.095 | 1.269 |
| 416 | 1.131 | 1.377 |
| 417 | 1.154 | 1.555 |
| 418 | 1.231 | 1.534 |
| 419 | 1.226 | 1.443 |
| 420 | 1.43 | 1.657 |
| 421 | 1.47 | 1.469 |
| 422 | 1.652 | 1.21 |
| 423 | 1.683 | 1.238 |
| 424 | 1.601 | 1.086 |
| 425 | 1.634 | 0.962 |
| 426 | 1.62 | 0.887 |
| 427 | 1.4 | 0.857 |
| 428 | 1.378 | 0.889 |
| 429 | 1.439 | 0.848 |
| 430 | 1.65 | 0.782 |
| 431 | 1.593 | 0.837 |
| 432 | 1.406 | 0.913 |
| 433 | 1.467 | 1.018 |
| 434 | 1.352 | 1.117 |
| 435 | 1.29 | 1.244 |
| 436 | 1.225 | 1.197 |
| 437 | 1.269 | 1.32 |
| 438 | 1.335 | 1.425 |
| 439 | 1.385 | 1.232 |
| 440 | 1.396 | 1.309 |
| 441 | 1.624 | 1.425 |
| 442 | 1.664 | 1.667 |
| 443 | 2.022 | 1.364 |
| 444 | 2.107 | 1.465 |
| 445 | 1.953 | 1.385 |
| 446 | 2.143 | 1.165 |
| 447 | 1.987 | 1.247 |
| 448 | 2.03 | 1.131 |
| 449 | 1.816 | 0.999 |
| 450 | 1.983 | 0.924 |
| 451 | 1.771 | 0.863 |
| 452 | 1.577 | 0.882 |
| 453 | 1.597 | 0.862 |
| 454 | 1.496 | 0.861 |
| 455 | 1.441 | 1.015 |
| 456 | 1.394 | 1.057 |
| 457 | 1.461 | 1.306 |
| 458 | 1.561 | 1.396 |
| 459 | 1.642 | 1.189 |
| 460 | 1.663 | 1.222 |
| 461 | 2.048 | 1.109 |
| 462 | 2.156 | 1.284 |
| 463 | 2.494 | 1.397 |
| 464 | 2.289 | 1.402 |
| 465 | 2.142 | 1.496 |
| 466 | 2.212 | 1.881 |
| 467 | 2.312 | 1.73 |
| 468 | 2.058 | 1.393 |
| 469 | 2.246 | 1.709 |
| 470 | 2.017 | 1.49 |
| 471 | 1.788 | 1.297 |
| 472 | 1.785 | 1.4 |
| 473 | 1.847 | 1.253 |
| 474 | 1.713 | 1.058 |
| 475 | 1.829 | 0.98 |
| 476 | 1.74 | 0.915 |
| 477 | 1.722 | 0.975 |
| 478 | 1.657 | 0.945 |
| 479 | 1.78 | 0.9 |
| 480 | 1.905 | 1.034 |
| 481 | 2.034 | 1.065 |
| 482 | 2.032 | 1.276 |
| 483 | 2.328 | 1.33 |
| 484 | 2.316 | 1.33 |
| 485 | 2.608 | 1.438 |
| 486 | 2.721 | 1.638 |
| 487 | 2.643 | 1.713 |
| 488 | 2.72 | 1.535 |
| 489 | 2.754 | 1.462 |
| 490 | 2.65 | 1.814 |
| 491 | 2.346 | 1.942 |
| 492 | 2.348 | 1.666 |
| 493 | 2.513 | 1.887 |
| 494 | 2.372 | 1.765 |
| 495 | 2.153 | 1.443 |
| 496 | 2.386 | 1.576 |
| 497 | 2.134 | 1.382 |
| 498 | 2.049 | 1.172 |
| 499 | 2.128 | 1.107 |
| 500 | 2.067 | 1.029 |
| 501 | 1.999 | 1.097 |
| 502 | 2.065 | 1.104 |
| 503 | 2.171 | 1.083 |
| 504 | 2.294 | 1.435 |
| 505 | 2.288 | 1.44 |
| 506 | 2.281 | 1.739 |
| 507 | 2.552 | 1.783 |
| 508 | 2.598 | 1.731 |
| 509 | 2.893 | 1.832 |
| 510 | 3.111 | 1.685 |
| 511 | 3.357 | 1.801 |
| 512 | 3.433 | 1.7 |
| 513 | 3.496 | 1.646 |
| 514 | 3.233 | 1.901 |
| 515 | 2.898 | 2.037 |
| 516 | 3.016 | 1.786 |
| 517 | 2.791 | 2.016 |
| 518 | 2.626 | 1.863 |
| 519 | 2.834 | 1.64 |
| 520 | 2.915 | 1.762 |
| 521 | 2.666 | 1.595 |
| 522 | 2.661 | 1.377 |
| 523 | 2.922 | 1.28 |
| 524 | 2.862 | 1.225 |
| 525 | 2.738 | 1.343 |
| 526 | 3.075 | 1.373 |
| 527 | 2.888 | 1.272 |
| 528 | 2.447 | 1.566 |
| 529 | 2.434 | 1.717 |
| 530 | 2.888 | 2.093 |
| 531 | 2.551 | 2.073 |
| 532 | 2.705 | 2.279 |
| 533 | 2.586 | 2.509 |
| 534 | 2.854 | 2.627 |
| 535 | 2.989 | 2.254 |
| 536 | 3.101 | 2.28 |
| 537 | 3.569 | 2.115 |
| 538 | 3.607 | 2.212 |
| 539 | 3.395 | 1.957 |
| 540 | 3.29 | 1.923 |
| 541 | 2.994 | 2.15 |
| 542 | 2.895 | 2.472 |
| 543 | 2.959 | 2.113 |
| 544 | 3.272 | 1.938 |
| 545 | 3.305 | 2.08 |
| 546 | 3.441 | 1.939 |
| 547 | 3.751 | 1.662 |
| 548 | 4.621 | 1.598 |
| 549 | 3.204 | 1.625 |
| 550 | 3.152 | 1.756 |
| 551 | 3.033 | 1.785 |
| 552 | 2.894 | 1.656 |
| 553 | 3.515 | 1.76 |
| 554 | 3.95 | 1.825 |
| 555 | 5.037 | 2.084 |
| 556 | 6.188 | 2.322 |
| 557 | 5.278 | 2.486 |
| 558 | 5.625 | 2.721 |
| 559 | 5.862 | 2.635 |
| 560 | 5.51 | 2.26 |
| 561 | 4.659 | 2.491 |
| 562 | 5.256 | 2.439 |
| 563 | 5.812 | 2.158 |
| 564 | 5.439 | 2.275 |
| 565 | 5.149 | 2.418 |
| 566 | 4.397 | 2.269 |
| 567 | 4.777 | 2.284 |
| 568 | 4.984 | 2.396 |
| 569 | 4.293 | 2.657 |
| 570 | 4.171 | 2.42 |
| 571 | 4.914 | 2.235 |
| 572 | 4.933 | 2.306 |
| 573 | 4.65 | 2.122 |
| 574 | 5.21 | 2.016 |
| 575 | 5.482 | 2.12 |
| 576 | 5.133 | 2.138 |
| 577 | 5.202 | 2.194 |
| 578 | 5.357 | 2.525 |
| 579 | 5.781 | 2.431 |
| 580 | 5.415 | 2.202 |
| 581 | 4.894 | 2.19 |
| 582 | 5.011 | 2.303 |
| 583 | 3.666 | 2.624 |
| 584 | 3.242 | 3.045 |
| 585 | 2.774 | 3.45 |
| 586 | 2.667 | 3.846 |
| 587 | 3.005 | 3.995 |
| 588 | 2.631 | 3.563 |
| 589 | 2.93 | 3.374 |
| 590 | 2.515 | 3.19 |
| 591 | 2.045 | 2.741 |
| 592 | 2.347 | 2.355 |
| 593 | 2.197 | 2.313 |
| 594 | 2.325 | 2.395 |
| 595 | 2.953 | 2.604 |
| 596 | 2.777 | 2.697 |
| 597 | 2.638 | 3.047 |
| 598 | 2.603 | 2.957 |
| 599 | 2.931 | 3.493 |
| 600 | 2.698 | 3.613 |
| 601 | 2.736 | 8.135 |
| 602 | 2.568 | 5.939 |
| 603 | 3.074 | 4.242 |
| 604 | 3.33 | 3.642 |
| 605 | 4.979 | 3.855 |
| 606 | 5.129 | 3.843 |
| 607 | 4.287 | 3.363 |
| 608 | 4.346 | 3.291 |
| 609 | 4.09 | 3.609 |
| 610 | 3.523 | 3.093 |
| 611 | 3.301 | 3.304 |
| 612 | 3.198 | 3.533 |
| 613 | 3.504 | 2.757 |
| 614 | 3.928 | 2.821 |
| 615 | 4.572 | 3.391 |
| 616 | 5.401 | 3.218 |
| 617 | 6.479 | 3.362 |
| 618 | 8.319 | 3.308 |
| 619 | 8.9 | 3.251 |
| 620 | 9.55 | 3.303 |
| 621 | 8.559 | 3.307 |
| 622 | 7.941 | 3.244 |
| 623 | 8.455 | 3.284 |
| 624 | 6.478 | 3.347 |
| 625 | 5.75 | 3.507 |
| 626 | 5.847 | 3.821 |
| 627 | 5.997 | 3.203 |
| 628 | 5.444 | 2.87 |
| 629 | 5.176 | 3.021 |
| 630 | 5.718 | 3.548 |
| 631 | 5.631 | 3.411 |
| 632 | 5.235 | 2.576 |
| 633 | 5.569 | 2.259 |
| 634 | 6.613 | 1.77 |
| 635 | 6.7 | 1.543 |
| 636 | 7.483 | 1.403 |
| 637 | 7.32 | 1.754 |
| 638 | 6.544 | 1.999 |
| 639 | 6.552 | 2.192 |
| 640 | 6.369 | 2.019 |
| 641 | 5.851 | 2.539 |
| 642 | 6.018 | 2.254 |
| 643 | 6.091 | 1.673 |
| 644 | 5.263 | 1.356 |
| 645 | 5.712 | 1.477 |
| 646 | 6.462 | 1.563 |
| 647 | 7.015 | 2.184 |
| 648 | 6.584 | 2.034 |
| 649 | 5.463 | 1.555 |
| 650 | 4.55 | 1.825 |
| 651 | 3.801 | 1.848 |
| 652 | 3.881 | 2.221 |
| 653 | 3.491 | 2.995 |
| 654 | 3.359 | 2.501 |
| 655 | 3.303 | 2.105 |
| 656 | 3.5 | 1.711 |
| 657 | 3.898 | 1.681 |
| 658 | 4.384 | 2.111 |
| 659 | 4.818 | 2.238 |
| 660 | 4.31 | 2.602 |
| 661 | 3.918 | 2.581 |
| 662 | 4.664 | 2.479 |
| 663 | 4.587 | 2.112 |
| 664 | 4.121 | 1.837 |
| 665 | 4.613 | 1.902 |
| 666 | 5.231 | 1.854 |
| 667 | 5.212 | 2.382 |
| 668 | 5.153 | 2.949 |
| 669 | 5.712 | 3.511 |
| 670 | 6.027 | 3.832 |
| 671 | 5.959 | 5.645 |
| 672 | 6.119 | 5.223 |
| 673 | 5.681 | 4.428 |
| 674 | 5.658 | 4.049 |
| 675 | 4.809 | 4.286 |
| 676 | 4.346 | 3.988 |
| 677 | 3.599 | 3.444 |
| 678 | 2.908 | 3.543 |
| 679 | 3.541 | 3.631 |
| 680 | 3.754 | 3.161 |
| 681 | 4.067 | 2.91 |
| 682 | 3.385 | 3.187 |
| 683 | 3.21 | 3.457 |
| 684 | 3.19 | 3.216 |
| 685 | 3.203 | 2.76 |
| 686 | 2.743 | 2.623 |
| 687 | 1.919 | 2.173 |
| 688 | 1.783 | 2.143 |
| 689 | 1.779 | 2.122 |
| 690 | 1.835 | 2.537 |
| 691 | 1.751 | 2.564 |
| 692 | 1.766 | 2.369 |
| 693 | 1.833 | 3.255 |
| 694 | 1.836 | 3.329 |
| 695 | 1.789 | 3.519 |
| 696 | 1.802 | 3.462 |
| 697 | 1.82 | 4.712 |
| 698 | 1.813 | 4.396 |
| 699 | 1.796 | 3.572 |
| 700 | 1.918 | 2.904 |
| 701 | 2.011 | 3.021 |
| 702 | 1.944 | 2.304 |
| 703 | 2.227 | 1.971 |
| 704 | 2.479 | 2.47 |
| 705 | 2.542 | 2.33 |
| 706 | 3.013 | 2.406 |
| 707 | 2.894 | 2.311 |
| 708 | 3.595 | 2.505 |
| 709 | 3.567 | 2.789 |
| 710 | 2.776 | 3.02 |
| 711 | 2.429 | 3.749 |
| 712 | 2.073 | 3.553 |
| 713 | 1.67 | 2.957 |
| 714 | 1.726 | 3.488 |
| 715 | 1.661 | 3.687 |
| 716 | 1.422 | 3.127 |
| 717 | 1.37 | 3.177 |
| 718 | 1.495 | 3.822 |
| 719 | 1.487 | 3.633 |
| 720 | 1.381 | 3.319 |
| 721 | 1.416 | 3.852 |
| 722 | 1.631 | 4.208 |
| 723 | 2.175 | 3.933 |
| 724 | 2.411 | 4.204 |
| 725 | 2.458 | 3.652 |
| 726 | 2.031 | 3.687 |
| 727 | 2.129 | 2.88 |
| 728 | 2.487 | 2.562 |
| 729 | 1.95 | 2.349 |
| 730 | 1.834 | 2.465 |
| 731 | 1.559 | 2.894 |
| 732 | 1.573 | 2.968 |
| 733 | 1.736 | 2.429 |
| 734 | 1.653 | 1.972 |
| 735 | 1.334 | 2.386 |
| 736 | 1.346 | 2.658 |
| 737 | 1.583 | 2.865 |
| 738 | 1.87 | 3.147 |
| 739 | 1.83 | 3.847 |
| 740 | 2.659 | 2.032 |
| 741 | 2.526 | 1.753 |
| 742 | 2.213 | 1.623 |
| 743 | 1.623 | 1.31 |
| 744 | 1.832 | 1.281 |
| 745 | 1.543 | 1.322 |
| 746 | 1.127 | 1.209 |
| 747 | 1.37 | 1.181 |
| 748 | 1.341 | 1.371 |
| 749 | 1.264 | 1.425 |
| 750 | 1.226 | 1.392 |
| 751 | 1.385 | 1.503 |
| 752 | 1.365 | 1.571 |
| 753 | 1.757 | 1.539 |
| 754 | 1.617 | 1.427 |
| 755 | 1.487 | 1.637 |
| 756 | 1.435 | 1.929 |
| 757 | 1.725 | 1.945 |
| 758 | 1.552 | 2.079 |
| 759 | 1.503 | 1.867 |
| 760 | 1.632 | 1.948 |
| 761 | 1.404 | 2.132 |
| 762 | 1.43 | 1.942 |
| 763 | 1.7 | 1.847 |
| 764 | 1.68 | 1.835 |
| 765 | 2.077 | 1.34 |
| 766 | 2.152 | 1.562 |
| 767 | 1.896 | 1.995 |
| 768 | 1.855 | 2.001 |
| 769 | 2.142 | 2.174 |
| 770 | 2.274 | 2.108 |
| 771 | 2.016 | 1.581 |
| 772 | 2.086 | 1.411 |
| 773 | 2.309 | 1.554 |
| 774 | 2.219 | 1.564 |
| 775 | 2.015 | 1.564 |
| 776 | 1.812 | 1.571 |
| 777 | 1.789 | 2.16 |
| 778 | 1.788 | 2 |
| 779 | 1.53 | 2.797 |
| 780 | 1.488 | 2.443 |
| 781 | 1.65 | 2.578 |
| 782 | 1.531 | 2.374 |
| 783 | 1.29 | 1.265 |
| 784 | 1.419 | 1.115 |
| 785 | 1.455 | 1.238 |
| 786 | 1.285 | 1.474 |
| 787 | 1.245 | 1.482 |
| 788 | 1.313 | 1.378 |
| 789 | 1.327 | 1.908 |
| 790 | 1.331 | 2.624 |
| 791 | 1.387 | 2.578 |
| 792 | 1.719 | 1.981 |
| 793 | 1.991 | 1.961 |
| 794 | 2.726 | 1.905 |
| 795 | 3.212 | 1.363 |
| 796 | 2.978 | 1.554 |
| 797 | 3.848 | 1.57 |
| 798 | 4.52 | 1.532 |
| 799 | 3.902 | 1.551 |
| 800 | 3.939 | 1.688 |
| 801 | 4.652 | 1.517 |
| 802 | 4.083 | 1.315 |
| 803 | 3.156 | 1.324 |
| 804 | 2.189 | 1.723 |
| 805 | 2.135 | 1.766 |
| 806 | 1.917 | 1.643 |
| 807 | 1.674 | 1.968 |
| 808 | 2.167 | 1.665 |
| 809 | 2.087 | 1.758 |
| 810 | 2.408 | 1.819 |
| 811 | 2.477 | 1.998 |
| 812 | 2.045 | 1.978 |
| 813 | 2.452 | 1.579 |
| 814 | 2.722 | 1.563 |
| 815 | 2.927 | 1.589 |
| 816 | 3.362 | 1.361 |
| 817 | 3.375 | 1.543 |
| 818 | 3.522 | 1.879 |
| 819 | 4.101 | 1.912 |
| 820 | 4.645 | 1.955 |
| 821 | 5.147 | 2.33 |
| 822 | 5.341 | 2.488 |
| 823 | 4.254 | 2.428 |
| 824 | 3.745 | 2.634 |
| 825 | 4.167 | 2.686 |
| 826 | 4.676 | 3.642 |
| 827 | 4.401 | 4.997 |
| 828 | 3.393 | 3.783 |
| 829 | 2.732 | 2.908 |
| 830 | 2.917 | 2.854 |
| 831 | 2.663 | 2.535 |
| 832 | 2.548 | 1.959 |
| 833 | 1.901 | 2.035 |
| 834 | 1.481 | 1.952 |
| 835 | 2.071 | 1.943 |
| 836 | 1.324 | 1.841 |
| 837 | 1.217 | 1.826 |
| 838 | 1.447 | 1.843 |
| 839 | 1.494 | 1.478 |
| 840 | 1.438 | 1.461 |
| 841 | 1.749 | 1.906 |
| 842 | 1.91 | 2.185 |
| 843 | 1.871 | 2.103 |
| 844 | 1.81 | 2.187 |
| 845 | 1.854 | 1.625 |
| 846 | 2.016 | 1.602 |
| 847 | 2.507 | 1.679 |
| 848 | 2.985 | 1.611 |
| 849 | 2.811 | 1.893 |
| 850 | 3.358 | 1.649 |
| 851 | 3.38 | 1.649 |
| 852 | 4.021 | 1.763 |
| 853 | 3.554 | 2.023 |
| 854 | 3.294 | 2.182 |
| 855 | 3.44 | 2.245 |
| 856 | 3.515 | 2.585 |
| 857 | 3.262 | 3.356 |
| 858 | 2.823 | 3.267 |
| 859 | 2.486 | 2.956 |
| 860 | 2.421 | 3.1 |
| 861 | 2.269 | 3.684 |
| 862 | 1.775 | 3.122 |
| 863 | 1.733 | 2.899 |
| 864 | 1.744 | 2.782 |
| 865 | 1.719 | 2.799 |
| 866 | 1.676 | 3.565 |
| 867 | 1.575 | 3.89 |
| 868 | 1.632 | 2.985 |
| 869 | 1.952 | 2.459 |
| 870 | 2.09 | 3.089 |
| 871 | 1.723 | 2.849 |
| 872 | 1.762 | 2.49 |
| 873 | 1.475 | 2.679 |
| 874 | 1.581 | 2.074 |
| 875 | 2.861 | 2.086 |
| 876 | 2.677 | 2.109 |
| 877 | 2.742 | 2.25 |
| 878 | 2.685 | 1.971 |
| 879 | 2.69 | 1.779 |
| 880 | 3.069 | 1.259 |
| 881 | 3.344 | 1.059 |
| 882 | 3.593 | 1.368 |
| 883 | 4.406 | 1.394 |
| 884 | 4.053 | 1.423 |
| 885 | 3.683 | 1.433 |
| 886 | 4.151 | 1.702 |
| 887 | 3.365 | 1.6 |
| 888 | 2.643 | 1.206 |
| 889 | 2.722 | 1.409 |
| 890 | 3.097 | 1.673 |
| 891 | 3.918 | 1.422 |
| 892 | 4.364 | 1.472 |
| 893 | 4.962 | 1.711 |
| 894 | 5.812 | 1.823 |
| 895 | 5.941 | 1.86 |
| 896 | 5.481 | 2.302 |
| 897 | 4.692 | 2.489 |
| 898 | 4.415 | 2.229 |
| 899 | 5.178 | 2.172 |
| 900 | 6.197 | 2.089 |
| 901 | 5.878 | 2.167 |
| 902 | 5.982 | 2.192 |
| 903 | 6.664 | 2.111 |
| 904 | 6.916 | 2.286 |
| 905 | 8.286 | 2.319 |
| 906 | 9.718 | 2.147 |
| 907 | 0.331 | 2.062 |
| 908 | 8.47 | 1.964 |
| 909 | 7.758 | 1.955 |
| 910 | 6.496 | 1.892 |
| 911 | 5.808 | 1.888 |
| 912 | 5.95 | 1.928 |
| 913 | 7.299 | 1.847 |
| 914 | 8.727 | 1.711 |
| 915 | 9.471 | 1.671 |
| 916 | 9.411 | 1.786 |
| 917 | 9.732 | 1.693 |
| 918 | 8.024 | 1.407 |
| 919 | 7.022 | 1.526 |
| 920 | 7.803 | 1.7 |
| 921 | 7.115 | 1.528 |
| 922 | 6.305 | 1.71 |
| 923 | 6.559 | 1.709 |
| 924 | 6.446 | 2.126 |
| 925 | 5.161 | 2.062 |
| 926 | 4.85 | 2.65 |
| 927 | 4.417 | 3.524 |
| 928 | 4.077 | 2.911 |
| 929 | 3.142 | 2.624 |
| 930 | 2.263 | 1.977 |
| 931 | 2.108 | 1.924 |
| 932 | 2.018 | 1.852 |
| 933 | 2.629 | 1.725 |
| 934 | 2.669 | 2.318 |
| 935 | 2.628 | 2.59 |
| 936 | 2.784 | 3.343 |
| 937 | 2.538 | 2.82 |
| 938 | 2.22 | 2.23 |
| 939 | 2.135 | 2.89 |
| 940 | 2.153 | 3.076 |
| 941 | 2.13 | 3.038 |
| 942 | 1.826 | 3.371 |
| 943 | 1.844 | 4.453 |
| 944 | 1.757 | 4.774 |
| 945 | 1.686 | 4.259 |
| 946 | 1.823 | 3.535 |
| 947 | 2.004 | 3.376 |
| 948 | 2.248 | 2.989 |
| 949 | 2.142 | 3.656 |
| 950 | 2.044 | 3.001 |
| 951 | 2.382 | 2.97 |
| 952 | 2.71 | 3.261 |
| 953 | 3.037 | 3.177 |
| 954 | 4.098 | 2.881 |
| 955 | 4.376 | 3.185 |
| 956 | 5.823 | 3.324 |
| 957 | 6.821 | 2.969 |
| 958 | 6.821 | 3.552 |
| 959 | 7.818 | 4.062 |
| 960 | 6.923 | 3.927 |
| 961 | 5.192 | 3.754 |
| 962 | 5.842 | 3.665 |
| 963 | 5.064 | 3.276 |
| 964 | 3.717 | 3.954 |
| 965 | 3.85 | 4.077 |
| 966 | 3.57 | 3.67 |
| 967 | 3.788 | 3.103 |
| 968 | 3.144 | 3.061 |
| 969 | 2.308 | 3.182 |
| 970 | 2.714 | 2.784 |
| 971 | 2.773 | 2.454 |
| 972 | 2.038 | 2.395 |
| 973 | 1.783 | 2.569 |
| 974 | 2.038 | 2.551 |
| 975 | 2.037 | 2.412 |
| 976 | 1.926 | 2.514 |
| 977 | 1.957 | 2.332 |
| 978 | 1.688 | 2.311 |
| 979 | 1.385 | 2.478 |
| 980 | 1.261 | 2.532 |
| 981 | 1.108 | 2.318 |
| 982 | 1.13 | 2.213 |
| 983 | 1.054 | 2.5 |
| 984 | 1.066 | 2.025 |
| 985 | 1.341 | 2.083 |
| 986 | 1.903 | 2.518 |
| 987 |  | 3.424 |
| 988 |  | 3.795 |
| 989 |  | 4.351 |
| 990 |  | 3.427 |
| 991 |  | 2.694 |
| 992 |  | 2.883 |
| 993 |  | 2.378 |
| 994 |  | 2.127 |
| 995 |  | 1.934 |
| 996 |  | 2.058 |
| 997 |  | 2.227 |
| 998 |  | 1.993 |
| 999 |  | 1.888 |
| 1000 |  | 1.974 |
| 1001 |  | 1.985 |
| 1002 |  | 2.045 |
| 1003 |  | 2.668 |
| 1004 |  | 3.091 |
| 1005 |  | 4.049 |
| 1006 |  | 3.128 |
| 1007 |  | 3.169 |
| 1008 |  | 3.582 |
| 1009 |  | 2.794 |
| 1010 |  | 2.748 |
| 1011 |  | 3.208 |
| 1012 |  | 2.647 |
| 1013 |  | 2.432 |
| 1014 |  | 1.887 |
| 1015 |  | 1.739 |
| 1016 |  | 1.698 |
| 1017 |  | 1.736 |
| 1018 |  | 1.612 |
| 1019 |  | 1.685 |
| 1020 |  | 1.755 |
| 1021 |  | 1.663 |
| 1022 |  | 1.697 |
| 1023 |  | 1.609 |
| 1024 |  | 1.703 |
| 1025 |  | 1.997 |
| 1026 |  | 1.492 |
| 1027 |  | 1.675 |
| 1028 |  | 1.739 |
| 1029 |  | 1.885 |
| 1030 |  | 1.601 |
| 1031 |  | 1.26 |
| 1032 |  | 1.343 |
| 1033 |  | 0.996 |
| 1034 |  | 1.139 |
| 1035 |  | 1.424 |
| 1036 |  | 1.687 |
| 1037 |  | 2.028 |
| 1038 |  | 1.852 |
