# Supplementary material for: Immunoinformatics and reverse vaccinology design of self-amplifying RNA vaccine against marburg virus with structural dynamics and immune simulation analysis
Source: PLoS One. 2026 Oct 23;21(10):e0361136. doi: 10.1371/journal.pone.0361136 (PMC13693908; doi:10.1371/journal.pone.0361136)
Supplement: Supplementary file 5 [file pone.0361136.s004.docx]

**Protein rGyr**

| **Frame** | **Time(ns)** | **MB-mVax-TLR2** | **MB-mVax-TLR4** |
| --- | --- | --- | --- |
| 0 | 0 | 29.855 | 31.748 |
| 1 | 0.2 | 30.11 | 32.817 |
| 2 | 0.4 | 29.972 | 32.757 |
| 3 | 0.6 | 29.915 | 33.555 |
| 4 | 0.8 | 30.034 | 33.252 |
| 5 | 1 | 29.739 | 33.317 |
| 6 | 1.2 | 29.768 | 33.833 |
| 7 | 1.4 | 29.785 | 33.611 |
| 8 | 1.6 | 30.068 | 34.716 |
| 9 | 1.8 | 30.198 | 34.771 |
| 10 | 2 | 30.357 | 35.165 |
| 11 | 2.2 | 30.19 | 35.708 |
| 12 | 2.4 | 30.272 | 35.631 |
| 13 | 2.6 | 29.734 | 35.141 |
| 14 | 2.8 | 30.084 | 35.489 |
| 15 | 3 | 29.642 | 34.893 |
| 16 | 3.2 | 30.023 | 35.38 |
| 17 | 3.4 | 30.938 | 34.847 |
| 18 | 3.6 | 30.534 | 35.961 |
| 19 | 3.8 | 30.17 | 36.301 |
| 20 | 4 | 30.299 | 36.283 |
| 21 | 4.2 | 30.751 | 37.405 |
| 22 | 4.4 | 32.07 | 37.262 |
| 23 | 4.6 | 32.163 | 37.152 |
| 24 | 4.8 | 32.634 | 37.155 |
| 25 | 5 | 32.792 | 37.58 |
| 26 | 5.2 | 32.176 | 37.167 |
| 27 | 5.4 | 32.82 | 36.749 |
| 28 | 5.6 | 30.989 | 36.63 |
| 29 | 5.8 | 30.989 | 36.43 |
| 30 | 6 | 31.361 | 35.843 |
| 31 | 6.2 | 31.058 | 35.737 |
| 32 | 6.4 | 30.276 | 35.543 |
| 33 | 6.6 | 29.971 | 36.436 |
| 34 | 6.8 | 30.218 | 35.951 |
| 35 | 7 | 30.411 | 36.373 |
| 36 | 7.2 | 30.005 | 36.275 |
| 37 | 7.4 | 30.098 | 36.296 |
| 38 | 7.6 | 29.648 | 36.619 |
| 39 | 7.8 | 30.115 | 36.282 |
| 40 | 8 | 29.896 | 36.146 |
| 41 | 8.2 | 29.879 | 35.817 |
| 42 | 8.4 | 30.013 | 35.987 |
| 43 | 8.6 | 30.18 | 36.319 |
| 44 | 8.8 | 29.723 | 36.243 |
| 45 | 9 | 30.4 | 36.313 |
| 46 | 9.2 | 30.609 | 36.827 |
| 47 | 9.4 | 30.553 | 37.349 |
| 48 | 9.6 | 30.803 | 38.461 |
| 49 | 9.8 | 31.377 | 38.7 |
| 50 | 10 | 33.471 | 39.262 |
| 51 | 10.2 | 34.424 | 39.401 |
| 52 | 10.4 | 34.584 | 39.536 |
| 53 | 10.6 | 36.456 | 38.877 |
| 54 | 10.8 | 37.166 | 37.582 |
| 55 | 11 | 37.291 | 36.589 |
| 56 | 11.2 | 37.389 | 34.053 |
| 57 | 11.4 | 37.617 | 32.096 |
| 58 | 11.6 | 36.62 | 32.202 |
| 59 | 11.8 | 37.308 | 32.297 |
| 60 | 12 | 37.558 | 32.056 |
| 61 | 12.2 | 37.626 | 32.108 |
| 62 | 12.4 | 36.981 | 32.642 |
| 63 | 12.6 | 36.58 | 32.917 |
| 64 | 12.8 | 36.367 | 34.009 |
| 65 | 13 | 36.365 | 35.451 |
| 66 | 13.2 | 37.11 | 35.364 |
| 67 | 13.4 | 36.585 | 35.639 |
| 68 | 13.6 | 36.171 | 35.483 |
| 69 | 13.8 | 35.973 | 35.692 |
| 70 | 14 | 35.437 | 35.825 |
| 71 | 14.2 | 34.115 | 36.42 |
| 72 | 14.4 | 33.008 | 36.833 |
| 73 | 14.6 | 29.8 | 36.45 |
| 74 | 14.8 | 32.006 | 37.173 |
| 75 | 15 | 31.907 | 37.475 |
| 76 | 15.2 | 31.544 | 37.515 |
| 77 | 15.4 | 30.925 | 38.517 |
| 78 | 15.6 | 30.415 | 38.743 |
| 79 | 15.8 | 29.713 | 38.974 |
| 80 | 16 | 29.543 | 37.902 |
| 81 | 16.2 | 29.874 | 35.636 |
| 82 | 16.4 | 30.013 | 33.884 |
| 83 | 16.6 | 29.997 | 32.052 |
| 84 | 16.8 | 29.995 | 32.118 |
| 85 | 17 | 29.926 | 32.271 |
| 86 | 17.2 | 29.559 | 32.286 |
| 87 | 17.4 | 30.152 | 32.793 |
| 88 | 17.6 | 30.057 | 34.032 |
| 89 | 17.8 | 30.209 | 35.145 |
| 90 | 18 | 30.436 | 35.755 |
| 91 | 18.2 | 30.465 | 35.611 |
| 92 | 18.4 | 30.352 | 35.204 |
| 93 | 18.6 | 30.213 | 35.629 |
| 94 | 18.8 | 30.008 | 36.08 |
| 95 | 19 | 30.128 | 36.418 |
| 96 | 19.2 | 30.145 | 36.786 |
| 97 | 19.4 | 30.263 | 38.027 |
| 98 | 19.6 | 30.263 | 38.892 |
| 99 | 19.8 | 30.561 | 37.505 |
| 100 | 20 | 30.39 | 34.156 |
| 101 | 20.2 | 30.772 | 32.827 |
| 102 | 20.4 | 30.243 | 32.915 |
| 103 | 20.6 | 30.194 | 33.154 |
| 104 | 20.8 | 30.168 | 34.319 |
| 105 | 21 | 30.37 | 35.28 |
| 106 | 21.2 | 30.667 | 35.865 |
| 107 | 21.4 | 30.663 | 35.577 |
| 108 | 21.6 | 30.152 | 35.813 |
| 109 | 21.8 | 30.299 | 35.978 |
| 110 | 22 | 30.184 | 36.182 |
| 111 | 22.2 | 30.247 | 36.867 |
| 112 | 22.4 | 30.383 | 36.994 |
| 113 | 22.6 | 30.245 | 37.188 |
| 114 | 22.8 | 30.099 | 37.161 |
| 115 | 23 | 30.213 | 37.378 |
| 116 | 23.2 | 29.819 | 37.17 |
| 117 | 23.4 | 29.807 | 36.358 |
| 118 | 23.6 | 30.376 | 36.928 |
| 119 | 23.8 | 30.054 | 37.997 |
| 120 | 24 | 30.167 | 38.923 |
| 121 | 24.2 | 30.447 | 38.74 |
| 122 | 24.4 | 30.787 | 39.536 |
| 123 | 24.6 | 30.38 | 40.355 |
| 124 | 24.8 | 30.16 | 39.783 |
| 125 | 25 | 29.954 | 38.937 |
| 126 | 25.2 | 29.952 | 37.855 |
| 127 | 25.4 | 30.332 | 37.172 |
| 128 | 25.6 | 30.449 | 37.088 |
| 129 | 25.8 | 30.906 | 35.605 |
| 130 | 26 | 30.288 | 34.228 |
| 131 | 26.2 | 30.315 | 32.885 |
| 132 | 26.4 | 29.856 | 32.758 |
| 133 | 26.6 | 30.055 | 33.133 |
| 134 | 26.8 | 29.915 | 33.457 |
| 135 | 27 | 29.551 | 33.506 |
| 136 | 27.2 | 30.037 | 33.305 |
| 137 | 27.4 | 30.381 | 33.029 |
| 138 | 27.6 | 29.968 | 33.068 |
| 139 | 27.8 | 30.279 | 32.819 |
| 140 | 28 | 30.328 | 32.632 |
| 141 | 28.2 | 30.744 | 32.975 |
| 142 | 28.4 | 30.417 | 33.06 |
| 143 | 28.6 | 29.571 | 32.966 |
| 144 | 28.8 | 29.662 | 33.039 |
| 145 | 29 | 29.777 | 32.977 |
| 146 | 29.2 | 30.026 | 33.653 |
| 147 | 29.4 | 30.268 | 34.731 |
| 148 | 29.6 | 29.685 | 36.57 |
| 149 | 29.8 | 30.124 | 37.976 |
| 150 | 30 | 30.204 | 38.485 |
| 151 | 30.2 | 30.424 | 37.775 |
| 152 | 30.4 | 30.138 | 37.106 |
| 153 | 30.6 | 30.517 | 36.832 |
| 154 | 30.8 | 29.976 | 37.382 |
| 155 | 31 | 30.187 | 36.791 |
| 156 | 31.2 | 30.371 | 37.632 |
| 157 | 31.4 | 30.546 | 38.093 |
| 158 | 31.6 | 30.214 | 37.742 |
| 159 | 31.8 | 30.32 | 37.911 |
| 160 | 32 | 30.167 | 37.387 |
| 161 | 32.2 | 30.121 | 37.013 |
| 162 | 32.4 | 29.757 | 36.918 |
| 163 | 32.6 | 29.775 | 37.51 |
| 164 | 32.8 | 30.072 | 38.139 |
| 165 | 33 | 29.914 | 38.608 |
| 166 | 33.2 | 30.27 | 38.391 |
| 167 | 33.4 | 30.504 | 38.301 |
| 168 | 33.6 | 30.618 | 38.24 |
| 169 | 33.8 | 29.682 | 37.661 |
| 170 | 34 | 29.835 | 36.812 |
| 171 | 34.2 | 30.086 | 36.542 |
| 172 | 34.4 | 30.34 | 34.968 |
| 173 | 34.6 | 30.351 | 33.384 |
| 174 | 34.8 | 29.888 | 32.904 |
| 175 | 35 | 29.942 | 32.96 |
| 176 | 35.2 | 30.454 | 32.86 |
| 177 | 35.4 | 30.444 | 33.194 |
| 178 | 35.6 | 30.365 | 32.894 |
| 179 | 35.8 | 30.372 | 32.46 |
| 180 | 36 | 30.713 | 32.931 |
| 181 | 36.2 | 30.551 | 33.391 |
| 182 | 36.4 | 30.409 | 33.183 |
| 183 | 36.6 | 30.482 | 34.907 |
| 184 | 36.8 | 30.463 | 36.562 |
| 185 | 37 | 30.408 | 37.579 |
| 186 | 37.2 | 30.569 | 38.071 |
| 187 | 37.4 | 30.394 | 38.808 |
| 188 | 37.6 | 30.449 | 39.608 |
| 189 | 37.8 | 30.64 | 39.747 |
| 190 | 38 | 30.633 | 39.857 |
| 191 | 38.2 | 29.967 | 40.209 |
| 192 | 38.4 | 30.5 | 40.373 |
| 193 | 38.6 | 29.967 | 40.507 |
| 194 | 38.8 | 30.098 | 32.563 |
| 195 | 39 | 30.257 | 32.493 |
| 196 | 39.2 | 30.601 | 32.333 |
| 197 | 39.4 | 30.267 | 32.041 |
| 198 | 39.6 | 30.451 | 31.973 |
| 199 | 39.8 | 30.089 | 32.647 |
| 200 | 40 | 30.276 | 32.224 |
| 201 | 40.2 | 30.562 | 32.079 |
| 202 | 40.4 | 29.62 | 32.315 |
| 203 | 40.6 | 30.28 | 32.26 |
| 204 | 40.8 | 30.302 | 32.402 |
| 205 | 41 | 30.587 | 31.889 |
| 206 | 41.2 | 30.888 | 31.88 |
| 207 | 41.4 | 30.559 | 32.236 |
| 208 | 41.6 | 30.917 | 32.311 |
| 209 | 41.8 | 30.869 | 32.075 |
| 210 | 42 | 30.244 | 32.562 |
| 211 | 42.2 | 30.476 | 31.94 |
| 212 | 42.4 | 30.529 | 32.087 |
| 213 | 42.6 | 30.562 | 37.661 |
| 214 | 42.8 | 30.033 | 36.72 |
| 215 | 43 | 30.176 | 36.399 |
| 216 | 43.2 | 30.662 | 35.291 |
| 217 | 43.4 | 30.081 | 34.829 |
| 218 | 43.6 | 30.271 | 35.38 |
| 219 | 43.8 | 29.953 | 35.655 |
| 220 | 44 | 30.109 | 36.192 |
| 221 | 44.2 | 30.055 | 36.133 |
| 222 | 44.4 | 30.007 | 35.134 |
| 223 | 44.6 | 29.88 | 34.794 |
| 224 | 44.8 | 30.44 | 33.959 |
| 225 | 45 | 30.619 | 34.677 |
| 226 | 45.2 | 30.404 | 34.989 |
| 227 | 45.4 | 30.676 | 34.402 |
| 228 | 45.6 | 30.619 | 34.518 |
| 229 | 45.8 | 30.702 | 34.318 |
| 230 | 46 | 30.538 | 34.312 |
| 231 | 46.2 | 30.365 | 34.44 |
| 232 | 46.4 | 30.684 | 34.29 |
| 233 | 46.6 | 30.246 | 34.37 |
| 234 | 46.8 | 30.606 | 34.335 |
| 235 | 47 | 30.206 | 34.316 |
| 236 | 47.2 | 30.353 | 33.942 |
| 237 | 47.4 | 29.998 | 34.86 |
| 238 | 47.6 | 30.495 | 35.892 |
| 239 | 47.8 | 29.939 | 35.708 |
| 240 | 48 | 30.439 | 36.252 |
| 241 | 48.2 | 30.154 | 35.992 |
| 242 | 48.4 | 30.273 | 36.172 |
| 243 | 48.6 | 30.554 | 36.362 |
| 244 | 48.8 | 30.77 | 36.247 |
| 245 | 49 | 30.885 | 36.363 |
| 246 | 49.2 | 30.376 | 36.655 |
| 247 | 49.4 | 30.255 | 36.389 |
| 248 | 49.6 | 29.99 | 35.601 |
| 249 | 49.8 | 30.446 | 35.923 |
| 250 | 50 | 30.004 | 36.142 |
| 251 | 50.2 | 30.224 | 36.496 |
| 252 | 50.4 | 29.995 | 36.512 |
| 253 | 50.6 | 30.445 | 36.74 |
| 254 | 50.8 | 29.71 | 36.561 |
| 255 | 51 | 30.403 | 36.444 |
| 256 | 51.2 | 30.308 | 36.518 |
| 257 | 51.4 | 30.557 | 36.844 |
| 258 | 51.6 | 30.677 | 36.803 |
| 259 | 51.8 | 30.788 | 36.81 |
| 260 | 52 | 30.213 | 36.886 |
| 261 | 52.2 | 29.982 | 36.919 |
| 262 | 52.4 | 30.242 | 36.706 |
| 263 | 52.6 | 30.197 | 37.154 |
| 264 | 52.8 | 30.402 | 36.808 |
| 265 | 53 | 29.916 | 36.522 |
| 266 | 53.2 | 30.489 | 36.341 |
| 267 | 53.4 | 30.315 | 36.271 |
| 268 | 53.6 | 30.276 | 36.141 |
| 269 | 53.8 | 29.789 | 36.071 |
| 270 | 54 | 30.216 | 36.865 |
| 271 | 54.2 | 30.132 | 36.572 |
| 272 | 54.4 | 29.841 | 36.827 |
| 273 | 54.6 | 30.214 | 36.371 |
| 274 | 54.8 | 30.312 | 36.978 |
| 275 | 55 | 30.248 | 37.487 |
| 276 | 55.2 | 30.509 | 38.1 |
| 277 | 55.4 | 30.879 | 37.106 |
| 278 | 55.6 | 30.441 | 37.292 |
| 279 | 55.8 | 30.184 | 37.205 |
| 280 | 56 | 30.327 | 37.152 |
| 281 | 56.2 | 30.457 | 37.162 |
| 282 | 56.4 | 30.439 | 36.661 |
| 283 | 56.6 | 29.983 | 36.38 |
| 284 | 56.8 | 30.443 | 36.42 |
| 285 | 57 | 30.169 | 36.688 |
| 286 | 57.2 | 30.265 | 36.875 |
| 287 | 57.4 | 30.218 | 37.312 |
| 288 | 57.6 | 30.162 | 37.275 |
| 289 | 57.8 | 29.914 | 37.091 |
| 290 | 58 | 29.806 | 37.434 |
| 291 | 58.2 | 30.413 | 37.407 |
| 292 | 58.4 | 30.988 | 37.278 |
| 293 | 58.6 | 30.524 | 37.634 |
| 294 | 58.8 | 30.163 | 38.5 |
| 295 | 59 | 30.379 | 38.532 |
| 296 | 59.2 | 30.444 | 38.122 |
| 297 | 59.4 | 30.265 | 31.564 |
| 298 | 59.6 | 30.068 | 31.748 |
| 299 | 59.8 | 29.845 | 31.633 |
| 300 | 60 | 30.11 | 39.425 |
| 301 | 60.2 | 30.29 | 39.428 |
| 302 | 60.4 | 30.173 | 39.069 |
| 303 | 60.6 | 30.051 | 31.631 |
| 304 | 60.8 | 30.281 | 31.325 |
| 305 | 61 | 29.625 | 31.615 |
| 306 | 61.2 | 30.119 | 31.405 |
| 307 | 61.4 | 29.824 | 31.68 |
| 308 | 61.6 | 30.114 | 31.824 |
| 309 | 61.8 | 30.115 | 31.916 |
| 310 | 62 | 30.086 | 32.193 |
| 311 | 62.2 | 29.592 | 31.849 |
| 312 | 62.4 | 29.803 | 31.747 |
| 313 | 62.6 | 29.413 | 32.148 |
| 314 | 62.8 | 29.666 | 32.059 |
| 315 | 63 | 30.392 | 31.966 |
| 316 | 63.2 | 30.214 | 32.591 |
| 317 | 63.4 | 29.733 | 32.138 |
| 318 | 63.6 | 29.813 | 31.966 |
| 319 | 63.8 | 29.83 | 31.775 |
| 320 | 64 | 30.018 | 31.807 |
| 321 | 64.2 | 30.093 | 31.904 |
| 322 | 64.4 | 30.172 | 31.879 |
| 323 | 64.6 | 30.022 | 31.802 |
| 324 | 64.8 | 30.037 | 32.223 |
| 325 | 65 | 29.599 | 32.214 |
| 326 | 65.2 | 29.579 | 31.721 |
| 327 | 65.4 | 30.126 | 32.092 |
| 328 | 65.6 | 30.241 | 32.323 |
| 329 | 65.8 | 29.933 | 32.335 |
| 330 | 66 | 29.885 | 32.506 |
| 331 | 66.2 | 29.656 | 32.348 |
| 332 | 66.4 | 29.86 | 32.388 |
| 333 | 66.6 | 30.071 | 31.624 |
| 334 | 66.8 | 29.848 | 32.123 |
| 335 | 67 | 30.359 | 32.455 |
| 336 | 67.2 | 29.886 | 32.613 |
| 337 | 67.4 | 29.568 | 32.516 |
| 338 | 67.6 | 29.588 | 32.895 |
| 339 | 67.8 | 30.158 | 32.548 |
| 340 | 68 | 30.367 | 32.45 |
| 341 | 68.2 | 30.457 | 31.941 |
| 342 | 68.4 | 30.462 | 32.236 |
| 343 | 68.6 | 29.846 | 32.297 |
| 344 | 68.8 | 29.802 | 32.229 |
| 345 | 69 | 29.888 | 32.264 |
| 346 | 69.2 | 30.05 | 31.661 |
| 347 | 69.4 | 30.11 | 32.097 |
| 348 | 69.6 | 29.676 | 32.441 |
| 349 | 69.8 | 29.563 | 32.317 |
| 350 | 70 | 29.59 | 32.326 |
| 351 | 70.2 | 29.61 | 32.062 |
| 352 | 70.4 | 29.45 | 32.168 |
| 353 | 70.6 | 29.553 | 32.492 |
| 354 | 70.8 | 29.908 | 32.232 |
| 355 | 71 | 30.135 | 32.074 |
| 356 | 71.2 | 30.014 | 32.338 |
| 357 | 71.4 | 29.81 | 32.171 |
| 358 | 71.6 | 30.025 | 32.077 |
| 359 | 71.8 | 30.233 | 32.222 |
| 360 | 72 | 29.94 | 32.108 |
| 361 | 72.2 | 29.779 | 31.779 |
| 362 | 72.4 | 30.259 | 32.478 |
| 363 | 72.6 | 30.169 | 32.55 |
| 364 | 72.8 | 29.761 | 32.656 |
| 365 | 73 | 29.735 | 32.627 |
| 366 | 73.2 | 30.161 | 32.573 |
| 367 | 73.4 | 30.128 | 32.579 |
| 368 | 73.6 | 29.817 | 32.401 |
| 369 | 73.8 | 30.286 | 32.534 |
| 370 | 74 | 29.455 | 32.376 |
| 371 | 74.2 | 30.187 | 32.369 |
| 372 | 74.4 | 30.31 | 32.244 |
| 373 | 74.6 | 29.952 | 32.105 |
| 374 | 74.8 | 29.915 | 31.955 |
| 375 | 75 | 29.893 | 32.234 |
| 376 | 75.2 | 29.588 | 32.221 |
| 377 | 75.4 | 29.337 | 32.196 |
| 378 | 75.6 | 29.819 | 32.26 |
| 379 | 75.8 | 29.909 | 32.09 |
| 380 | 76 | 30.23 | 32.119 |
| 381 | 76.2 | 30.541 | 32.269 |
| 382 | 76.4 | 30.256 | 32.41 |
| 383 | 76.6 | 30.208 | 32.163 |
| 384 | 76.8 | 30.347 | 32.238 |
| 385 | 77 | 30.267 | 32.143 |
| 386 | 77.2 | 30.31 | 32.355 |
| 387 | 77.4 | 29.962 | 32.77 |
| 388 | 77.6 | 30.795 | 32.576 |
| 389 | 77.8 | 30.088 | 32.81 |
| 390 | 78 | 30.395 | 32.386 |
| 391 | 78.2 | 29.985 | 32.522 |
| 392 | 78.4 | 29.902 | 32.219 |
| 393 | 78.6 | 29.624 | 32.095 |
| 394 | 78.8 | 29.697 | 32.331 |
| 395 | 79 | 29.672 | 32.88 |
| 396 | 79.2 | 29.62 | 32.302 |
| 397 | 79.4 | 29.44 | 32.429 |
| 398 | 79.6 | 29.341 | 32.228 |
| 399 | 79.8 | 29.876 | 32.46 |
| 400 | 80 | 29.858 | 32.13 |
| 401 | 80.2 | 29.263 | 31.979 |
| 402 | 80.4 | 29.307 | 31.976 |
| 403 | 80.6 | 29.551 | 32.151 |
| 404 | 80.8 | 29.849 | 31.948 |
| 405 | 81 | 30.018 | 32.586 |
| 406 | 81.2 | 30.015 | 32.198 |
| 407 | 81.4 | 30.081 | 32.321 |
| 408 | 81.6 | 29.768 | 32.312 |
| 409 | 81.8 | 29.148 | 32.127 |
| 410 | 82 | 29.294 | 32.144 |
| 411 | 82.2 | 29.45 | 32.525 |
| 412 | 82.4 | 29.588 | 32.153 |
| 413 | 82.6 | 29.703 | 31.918 |
| 414 | 82.8 | 29.95 | 32.277 |
| 415 | 83 | 30.171 | 32.189 |
| 416 | 83.2 | 29.879 | 32.489 |
| 417 | 83.4 | 29.921 | 32.121 |
| 418 | 83.6 | 30.086 | 32.271 |
| 419 | 83.8 | 29.628 | 31.858 |
| 420 | 84 | 29.743 | 31.79 |
| 421 | 84.2 | 29.819 | 32.214 |
| 422 | 84.4 | 29.845 | 31.811 |
| 423 | 84.6 | 29.49 | 31.999 |
| 424 | 84.8 | 29.765 | 32.065 |
| 425 | 85 | 30.272 | 31.919 |
| 426 | 85.2 | 30.147 | 32.355 |
| 427 | 85.4 | 29.909 | 32.188 |
| 428 | 85.6 | 30.097 | 32.118 |
| 429 | 85.8 | 30 | 32.239 |
| 430 | 86 | 29.649 | 32.062 |
| 431 | 86.2 | 29.652 | 31.914 |
| 432 | 86.4 | 29.542 | 31.756 |
| 433 | 86.6 | 29.067 | 32.026 |
| 434 | 86.8 | 28.843 | 31.357 |
| 435 | 87 | 28.869 | 31.723 |
| 436 | 87.2 | 29.496 | 32.161 |
| 437 | 87.4 | 29.149 | 32.365 |
| 438 | 87.6 | 29.389 | 32.737 |
| 439 | 87.8 | 29.678 | 32.15 |
| 440 | 88 | 29.597 | 32.198 |
| 441 | 88.2 | 29.476 | 32.124 |
| 442 | 88.4 | 29.379 | 32.12 |
| 443 | 88.6 | 29.137 | 32.266 |
| 444 | 88.8 | 29.379 | 32.489 |
| 445 | 89 | 29.662 | 32.139 |
| 446 | 89.2 | 29.593 | 32.202 |
| 447 | 89.4 | 29.425 | 32.009 |
| 448 | 89.6 | 29.537 | 32.039 |
| 449 | 89.8 | 29.669 | 32.22 |
| 450 | 90 | 29.36 | 32.13 |
| 451 | 90.2 | 29.946 | 32.325 |
| 452 | 90.4 | 29.864 | 32.19 |
| 453 | 90.6 | 30.378 | 32.646 |
| 454 | 90.8 | 29.419 | 32.482 |
| 455 | 91 | 29.901 | 32.32 |
| 456 | 91.2 | 29.76 | 32.457 |
| 457 | 91.4 | 29.827 | 32.323 |
| 458 | 91.6 | 29.693 | 32.342 |
| 459 | 91.8 | 30.104 | 32.131 |
| 460 | 92 | 29.843 | 32.187 |
| 461 | 92.2 | 30.509 | 32.56 |
| 462 | 92.4 | 30.06 | 32.284 |
| 463 | 92.6 | 30.071 | 32.286 |
| 464 | 92.8 | 30.44 | 32.202 |
| 465 | 93 | 30.07 | 32.708 |
| 466 | 93.2 | 29.808 | 32.315 |
| 467 | 93.4 | 29.969 | 32.452 |
| 468 | 93.6 | 30.576 | 32.457 |
| 469 | 93.8 | 29.833 | 32.316 |
| 470 | 94 | 29.718 | 32.493 |
| 471 | 94.2 | 29.661 | 32.08 |
| 472 | 94.4 | 30.279 | 31.784 |
| 473 | 94.6 | 30.268 | 32.394 |
| 474 | 94.8 | 30.596 | 32.002 |
| 475 | 95 | 30.175 | 32.239 |
| 476 | 95.2 | 30.085 | 32.291 |
| 477 | 95.4 | 30.121 | 32.096 |
| 478 | 95.6 | 29.777 | 31.944 |
| 479 | 95.8 | 29.759 | 32.064 |
| 480 | 96 | 29.802 | 32.15 |
| 481 | 96.2 | 29.801 | 32.069 |
| 482 | 96.4 | 30.004 | 32.016 |
| 483 | 96.6 | 30.002 | 32.073 |
| 484 | 96.8 | 30.224 | 32.143 |
| 485 | 97 | 30.451 | 32.165 |
| 486 | 97.2 | 31.057 | 32.72 |
| 487 | 97.4 | 29.912 | 32.15 |
| 488 | 97.6 | 30.044 | 32.296 |
| 489 | 97.8 | 30.124 | 32.804 |
| 490 | 98 | 30.136 | 32.693 |
| 491 | 98.2 | 30.39 | 32.284 |
| 492 | 98.4 | 30.135 | 32.026 |
| 493 | 98.6 | 30.111 | 32.233 |
| 494 | 98.8 | 30.194 | 32.341 |
| 495 | 99 | 29.95 | 32.341 |
| 496 | 99.2 | 30.338 | 32.434 |
| 497 | 99.4 | 30.292 | 32.448 |
| 498 | 99.6 | 30.43 | 32.577 |
| 499 | 99.8 | 30.168 | 32.198 |
| 500 | 100 | 29.794 | 32.415 |
| 501 | 100.2 | 29.891 | 32.144 |
| 502 | 100.4 | 30.194 | 31.961 |
| 503 | 100.6 | 30.672 | 32.033 |
| 504 | 100.8 | 30.131 | 32.418 |
| 505 | 101 | 30.116 | 32.269 |
| 506 | 101.2 | 29.73 | 31.882 |
| 507 | 101.4 | 29.971 | 32.189 |
| 508 | 101.6 | 29.746 | 32.86 |
| 509 | 101.8 | 29.915 | 32.171 |
| 510 | 102 | 29.949 | 32.69 |
| 511 | 102.2 | 30.42 | 31.804 |
| 512 | 102.4 | 30.037 | 31.836 |
| 513 | 102.6 | 29.783 | 31.994 |
| 514 | 102.8 | 30.098 | 32.002 |
| 515 | 103 | 29.942 | 32.061 |
| 516 | 103.2 | 30.027 | 32.579 |
| 517 | 103.4 | 29.673 | 32.434 |
| 518 | 103.6 | 29.891 | 32.402 |
| 519 | 103.8 | 29.828 | 31.829 |
| 520 | 104 | 30.027 | 32.376 |
| 521 | 104.2 | 30.001 | 32.201 |
| 522 | 104.4 | 29.642 | 32.154 |
| 523 | 104.6 | 29.578 | 31.849 |
| 524 | 104.8 | 29.462 | 32.15 |
| 525 | 105 | 29.755 | 32.099 |
| 526 | 105.2 | 29.875 | 31.847 |
| 527 | 105.4 | 30.216 | 31.641 |
| 528 | 105.6 | 30.701 | 31.771 |
| 529 | 105.8 | 30.599 | 32.463 |
| 530 | 106 | 29.966 | 32.305 |
| 531 | 106.2 | 30.079 | 32.248 |
| 532 | 106.4 | 30.791 | 32.46 |
| 533 | 106.6 | 30.464 | 32.566 |
| 534 | 106.8 | 30.359 | 31.946 |
| 535 | 107 | 30.153 | 32.155 |
| 536 | 107.2 | 30.002 | 32.041 |
| 537 | 107.4 | 30.082 | 31.815 |
| 538 | 107.6 | 30.421 | 31.88 |
| 539 | 107.8 | 30.245 | 31.64 |
| 540 | 108 | 30.218 | 32.053 |
| 541 | 108.2 | 30.339 | 31.929 |
| 542 | 108.4 | 30.358 | 32.184 |
| 543 | 108.6 | 30.627 | 31.665 |
| 544 | 108.8 | 30.618 | 31.985 |
| 545 | 109 | 30.274 | 31.882 |
| 546 | 109.2 | 30.173 | 31.986 |
| 547 | 109.4 | 30.223 | 31.582 |
| 548 | 109.6 | 30.524 | 32.109 |
| 549 | 109.8 | 30.387 | 31.473 |
| 550 | 110 | 30.286 | 31.649 |
| 551 | 110.2 | 30.237 | 32.134 |
| 552 | 110.4 | 30.418 | 31.93 |
| 553 | 110.6 | 30.059 | 31.894 |
| 554 | 110.8 | 30.417 | 32.095 |
| 555 | 111 | 30.541 | 34.61 |
| 556 | 111.2 | 29.992 | 31.452 |
| 557 | 111.4 | 29.964 | 35.095 |
| 558 | 111.6 | 30.174 | 32.426 |
| 559 | 111.8 | 29.952 | 32.356 |
| 560 | 112 | 30.012 | 35.828 |
| 561 | 112.2 | 30.355 | 36.747 |
| 562 | 112.4 | 30.046 | 37.346 |
| 563 | 112.6 | 30.09 | 38.399 |
| 564 | 112.8 | 30.101 | 38.125 |
| 565 | 113 | 29.724 | 38.044 |
| 566 | 113.2 | 29.793 | 38.124 |
| 567 | 113.4 | 29.839 | 38.353 |
| 568 | 113.6 | 29.663 | 38.209 |
| 569 | 113.8 | 30.176 | 38.358 |
| 570 | 114 | 30.474 | 38.262 |
| 571 | 114.2 | 30.088 | 38.122 |
| 572 | 114.4 | 30.245 | 38.56 |
| 573 | 114.6 | 30.589 | 37.929 |
| 574 | 114.8 | 30.301 | 38.052 |
| 575 | 115 | 30.352 | 38.057 |
| 576 | 115.2 | 30.112 | 38.026 |
| 577 | 115.4 | 30.298 | 38.221 |
| 578 | 115.6 | 30.119 | 38.194 |
| 579 | 115.8 | 30.447 | 38.345 |
| 580 | 116 | 29.796 | 38.371 |
| 581 | 116.2 | 30.37 | 38.819 |
| 582 | 116.4 | 30.407 | 38.783 |
| 583 | 116.6 | 30.395 | 38.419 |
| 584 | 116.8 | 30.022 | 37.95 |
| 585 | 117 | 29.967 | 37.803 |
| 586 | 117.2 | 30.407 | 37.851 |
| 587 | 117.4 | 30.232 | 37.846 |
| 588 | 117.6 | 30.26 | 38.501 |
| 589 | 117.8 | 29.87 | 38.121 |
| 590 | 118 | 29.75 | 38.044 |
| 591 | 118.2 | 29.388 | 38.25 |
| 592 | 118.4 | 29.477 | 37.939 |
| 593 | 118.6 | 29.749 | 38.423 |
| 594 | 118.8 | 29.846 | 38.485 |
| 595 | 119 | 29.894 | 38.383 |
| 596 | 119.2 | 30.42 | 38.006 |
| 597 | 119.4 | 30.27 | 37.798 |
| 598 | 119.6 | 29.802 | 37.741 |
| 599 | 119.8 | 30.139 | 38.27 |
| 600 | 120 | 30.622 | 38.245 |
| 601 | 120.2 | 30.324 | 38.304 |
| 602 | 120.4 | 30.523 | 38.451 |
| 603 | 120.6 | 30.475 | 37.639 |
| 604 | 120.8 | 30.435 | 37.695 |
| 605 | 121 | 30.451 | 36.894 |
| 606 | 121.2 | 30.25 | 35.807 |
| 607 | 121.4 | 30.098 | 34.782 |
| 608 | 121.6 | 29.723 | 33.923 |
| 609 | 121.8 | 30.088 | 33.291 |
| 610 | 122 | 30.278 | 32.145 |
| 611 | 122.2 | 30.477 | 31.997 |
| 612 | 122.4 | 30.011 | 32.138 |
| 613 | 122.6 | 30.177 | 31.843 |
| 614 | 122.8 | 30.293 | 31.863 |
| 615 | 123 | 30.238 | 31.929 |
| 616 | 123.2 | 30.112 | 31.94 |
| 617 | 123.4 | 30.037 | 31.499 |
| 618 | 123.6 | 30.309 | 31.779 |
| 619 | 123.8 | 31.134 | 31.575 |
| 620 | 124 | 30.947 | 32.38 |
| 621 | 124.2 | 30.316 | 32.927 |
| 622 | 124.4 | 30.355 | 32.825 |
| 623 | 124.6 | 30.714 | 32.923 |
| 624 | 124.8 | 31.01 | 33.191 |
| 625 | 125 | 30.911 | 33.954 |
| 626 | 125.2 | 30.587 | 34.559 |
| 627 | 125.4 | 30.942 | 35.12 |
| 628 | 125.6 | 30.782 | 36.132 |
| 629 | 125.8 | 30.398 | 36.645 |
| 630 | 126 | 30.181 | 36.928 |
| 631 | 126.2 | 29.983 | 37.293 |
| 632 | 126.4 | 30.193 | 37.596 |
| 633 | 126.6 | 30.177 | 37.765 |
| 634 | 126.8 | 30.806 | 37.746 |
| 635 | 127 | 30.65 | 37.828 |
| 636 | 127.2 | 30.343 | 37.715 |
| 637 | 127.4 | 30.783 | 37.519 |
| 638 | 127.6 | 30.526 | 37.361 |
| 639 | 127.8 | 30.468 | 37.57 |
| 640 | 128 | 30.412 | 37.467 |
| 641 | 128.2 | 30.954 | 37.123 |
| 642 | 128.4 | 30.597 | 37.085 |
| 643 | 128.6 | 30.674 | 37.135 |
| 644 | 128.8 | 30.511 | 37.415 |
| 645 | 129 | 30.559 | 37.315 |
| 646 | 129.2 | 30.669 | 37.204 |
| 647 | 129.4 | 30.554 | 37.426 |
| 648 | 129.6 | 30.38 | 37.38 |
| 649 | 129.8 | 30.583 | 37.076 |
| 650 | 130 | 30.799 | 37.503 |
| 651 | 130.2 | 30.674 | 37.432 |
| 652 | 130.4 | 30.739 | 37.002 |
| 653 | 130.6 | 30.781 | 36.935 |
| 654 | 130.8 | 30.242 | 36.63 |
| 655 | 131 | 30.28 | 36.298 |
| 656 | 131.2 | 30.221 | 35.656 |
| 657 | 131.4 | 30.163 | 35.697 |
| 658 | 131.6 | 30.145 | 35.727 |
| 659 | 131.8 | 30.128 | 36.131 |
| 660 | 132 | 30.209 | 36.774 |
| 661 | 132.2 | 29.638 | 36.554 |
| 662 | 132.4 | 29.792 | 36.914 |
| 663 | 132.6 | 30.283 | 37.542 |
| 664 | 132.8 | 30.09 | 37.973 |
| 665 | 133 | 30.095 | 38.318 |
| 666 | 133.2 | 30.351 | 38.409 |
| 667 | 133.4 | 30.349 | 37.991 |
| 668 | 133.6 | 29.984 | 37.59 |
| 669 | 133.8 | 29.77 | 35.817 |
| 670 | 134 | 30.212 | 33.579 |
| 671 | 134.2 | 30.671 | 32.235 |
| 672 | 134.4 | 30.15 | 32.138 |
| 673 | 134.6 | 29.812 | 31.637 |
| 674 | 134.8 | 30.428 | 31.397 |
| 675 | 135 | 30.451 | 31.732 |
| 676 | 135.2 | 30.053 | 32.47 |
| 677 | 135.4 | 30.509 | 32.967 |
| 678 | 135.6 | 30.791 | 34.38 |
| 679 | 135.8 | 30.852 | 35.49 |
| 680 | 136 | 29.962 | 36.873 |
| 681 | 136.2 | 30.106 | 38.069 |
| 682 | 136.4 | 30.638 | 38.002 |
| 683 | 136.6 | 30.521 | 36.239 |
| 684 | 136.8 | 30.566 | 33.354 |
| 685 | 137 | 30.524 | 31.729 |
| 686 | 137.2 | 30.477 | 31.658 |
| 687 | 137.4 | 30.581 | 31.782 |
| 688 | 137.6 | 30.687 | 31.986 |
| 689 | 137.8 | 31.302 | 33.025 |
| 690 | 138 | 30.505 | 34.65 |
| 691 | 138.2 | 30.111 | 36.411 |
| 692 | 138.4 | 30.257 | 36.979 |
| 693 | 138.6 | 30.246 | 37.527 |
| 694 | 138.8 | 30.514 | 38.236 |
| 695 | 139 | 30.352 | 37.554 |
| 696 | 139.2 | 30.005 | 35.385 |
| 697 | 139.4 | 30.548 | 34.293 |
| 698 | 139.6 | 30.192 | 32.295 |
| 699 | 139.8 | 30.198 | 31.984 |
| 700 | 140 | 30.111 | 31.712 |
| 701 | 140.2 | 30.011 | 31.9 |
| 702 | 140.4 | 30.082 | 32.8 |
| 703 | 140.6 | 29.642 | 33.282 |
| 704 | 140.8 | 30.141 | 34.339 |
| 705 | 141 | 30.324 | 34.338 |
| 706 | 141.2 | 30.404 | 35.53 |
| 707 | 141.4 | 30.356 | 36.648 |
| 708 | 141.6 | 30.463 | 37.17 |
| 709 | 141.8 | 30.715 | 38.169 |
| 710 | 142 | 30.783 | 38.137 |
| 711 | 142.2 | 30.555 | 38.394 |
| 712 | 142.4 | 30.699 | 38.525 |
| 713 | 142.6 | 30.1 | 37.366 |
| 714 | 142.8 | 30.444 | 36.208 |
| 715 | 143 | 30.636 | 34.203 |
| 716 | 143.2 | 30.399 | 33.372 |
| 717 | 143.4 | 30.513 | 32.747 |
| 718 | 143.6 | 30.618 | 31.927 |
| 719 | 143.8 | 30.737 | 31.945 |
| 720 | 144 | 30.719 | 31.919 |
| 721 | 144.2 | 30.457 | 32.33 |
| 722 | 144.4 | 30.503 | 32.364 |
| 723 | 144.6 | 30.257 | 33.06 |
| 724 | 144.8 | 30.303 | 33.417 |
| 725 | 145 | 30.144 | 34.714 |
| 726 | 145.2 | 30.247 | 36.072 |
| 727 | 145.4 | 30.297 | 36.943 |
| 728 | 145.6 | 30.261 | 37.931 |
| 729 | 145.8 | 29.952 | 38.284 |
| 730 | 146 | 30.287 | 37.979 |
| 731 | 146.2 | 30.113 | 36.579 |
| 732 | 146.4 | 30.079 | 34.907 |
| 733 | 146.6 | 30.472 | 33.163 |
| 734 | 146.8 | 30.013 | 32.5 |
| 735 | 147 | 30.423 | 32.171 |
| 736 | 147.2 | 29.869 | 31.962 |
| 737 | 147.4 | 30.105 | 32.417 |
| 738 | 147.6 | 29.928 | 33.248 |
| 739 | 147.8 | 30.012 | 34.628 |
| 740 | 148 | 30.113 | 35.965 |
| 741 | 148.2 | 30.313 | 36.926 |
| 742 | 148.4 | 30.024 | 38.135 |
| 743 | 148.6 | 30.586 | 37.974 |
| 744 | 148.8 | 30.166 | 35.854 |
| 745 | 149 | 30.335 | 34.072 |
| 746 | 149.2 | 29.965 | 32.221 |
| 747 | 149.4 | 29.875 | 31.83 |
| 748 | 149.6 | 29.94 | 31.972 |
| 749 | 149.8 | 30.157 | 32.684 |
| 750 | 150 | 30.216 | 33.704 |
| 751 | 150.2 | 30.467 | 36.103 |
| 752 | 150.4 | 30.039 | 37.808 |
| 753 | 150.6 | 30.186 | 38.275 |
| 754 | 150.8 | 30.489 | 37.422 |
| 755 | 151 | 30.584 | 34.701 |
| 756 | 151.2 | 30.936 | 32.721 |
| 757 | 151.4 | 30.618 | 32.368 |
| 758 | 151.6 | 30.466 | 31.897 |
| 759 | 151.8 | 30.815 | 32.57 |
| 760 | 152 | 30.599 | 33.158 |
| 761 | 152.2 | 30.347 | 34.99 |
| 762 | 152.4 | 30.268 | 35.976 |
| 763 | 152.6 | 30.14 | 37.498 |
| 764 | 152.8 | 30.09 | 38.099 |
| 765 | 153 | 30.072 | 37.892 |
| 766 | 153.2 | 30.014 | 36.051 |
| 767 | 153.4 | 30.601 | 33.208 |
| 768 | 153.6 | 30.606 | 31.787 |
| 769 | 153.8 | 30.779 | 31.623 |
| 770 | 154 | 30.838 | 31.962 |
| 771 | 154.2 | 30.826 | 32.755 |
| 772 | 154.4 | 30.544 | 33.942 |
| 773 | 154.6 | 30.716 | 34.99 |
| 774 | 154.8 | 30.778 | 36.303 |
| 775 | 155 | 30.723 | 37.388 |
| 776 | 155.2 | 30.517 | 38.015 |
| 777 | 155.4 | 30.827 | 37.772 |
| 778 | 155.6 | 30.703 | 36.712 |
| 779 | 155.8 | 30.815 | 34.912 |
| 780 | 156 | 31.102 | 33.426 |
| 781 | 156.2 | 30.978 | 32.078 |
| 782 | 156.4 | 30.509 | 31.761 |
| 783 | 156.6 | 30.422 | 31.605 |
| 784 | 156.8 | 30.832 | 31.793 |
| 785 | 157 | 30.9 | 32.763 |
| 786 | 157.2 | 30.931 | 33.822 |
| 787 | 157.4 | 30.347 | 35.246 |
| 788 | 157.6 | 30.935 | 36.716 |
| 789 | 157.8 | 30.799 | 37.929 |
| 790 | 158 | 30.799 | 37.909 |
| 791 | 158.2 | 30.496 | 36.691 |
| 792 | 158.4 | 30.858 | 34.667 |
| 793 | 158.6 | 30.601 | 32.363 |
| 794 | 158.8 | 30.737 | 31.983 |
| 795 | 159 | 30.394 | 32.322 |
| 796 | 159.2 | 30.944 | 32.317 |
| 797 | 159.4 | 30.69 | 32.459 |
| 798 | 159.6 | 30.961 | 32.7 |
| 799 | 159.8 | 30.372 | 33.204 |
| 800 | 160 | 30.064 | 34.31 |
| 801 | 160.2 | 30.323 | 35.883 |
| 802 | 160.4 | 30.259 | 36.727 |
| 803 | 160.6 | 30.182 | 37.591 |
| 804 | 160.8 | 30.117 | 38.463 |
| 805 | 161 | 29.833 | 37.073 |
| 806 | 161.2 | 30.357 | 34.494 |
| 807 | 161.4 | 30.366 | 33.276 |
| 808 | 161.6 | 30.749 | 32.446 |
| 809 | 161.8 | 30.278 | 32.222 |
| 810 | 162 | 30.698 | 31.869 |
| 811 | 162.2 | 30.123 | 32.047 |
| 812 | 162.4 | 30.312 | 31.848 |
| 813 | 162.6 | 30.349 | 32.191 |
| 814 | 162.8 | 30.882 | 32.717 |
| 815 | 163 | 30.184 | 33.099 |
| 816 | 163.2 | 30.073 | 33.759 |
| 817 | 163.4 | 30.032 | 34.488 |
| 818 | 163.6 | 30.127 | 35.42 |
| 819 | 163.8 | 30.186 | 36.448 |
| 820 | 164 | 29.941 | 37.182 |
| 821 | 164.2 | 30.376 | 37.276 |
| 822 | 164.4 | 30.501 | 37.963 |
| 823 | 164.6 | 30.552 | 38.285 |
| 824 | 164.8 | 30.286 | 38.283 |
| 825 | 165 | 30.503 | 38.27 |
| 826 | 165.2 | 30.251 | 38.149 |
| 827 | 165.4 | 30.064 | 37.482 |
| 828 | 165.6 | 29.618 | 37.681 |
| 829 | 165.8 | 29.861 | 37.182 |
| 830 | 166 | 29.952 | 36.279 |
| 831 | 166.2 | 30.143 | 35.674 |
| 832 | 166.4 | 30.706 | 36.097 |
| 833 | 166.6 | 30.229 | 34.821 |
| 834 | 166.8 | 30.859 | 35.026 |
| 835 | 167 | 30.554 | 33.97 |
| 836 | 167.2 | 30.153 | 32.852 |
| 837 | 167.4 | 30.406 | 32.416 |
| 838 | 167.6 | 30.451 | 32.111 |
| 839 | 167.8 | 30.712 | 31.875 |
| 840 | 168 | 30.652 | 31.865 |
| 841 | 168.2 | 30.348 | 31.794 |
| 842 | 168.4 | 30.385 | 31.902 |
| 843 | 168.6 | 30.675 | 32.445 |
| 844 | 168.8 | 30.344 | 32.625 |
| 845 | 169 | 30.453 | 32.698 |
| 846 | 169.2 | 30.328 | 33.308 |
| 847 | 169.4 | 30.378 | 33.456 |
| 848 | 169.6 | 30.539 | 34.604 |
| 849 | 169.8 | 30.455 | 35.456 |
| 850 | 170 | 30.232 | 36.552 |
| 851 | 170.2 | 30.592 | 37.591 |
| 852 | 170.4 | 29.887 | 38.117 |
| 853 | 170.6 | 30.402 | 38.246 |
| 854 | 170.8 | 29.735 | 38.141 |
| 855 | 171 | 30.448 | 37.99 |
| 856 | 171.2 | 30.31 | 37.139 |
| 857 | 171.4 | 30.582 | 36.171 |
| 858 | 171.6 | 30.293 | 34.772 |
| 859 | 171.8 | 30.296 | 33.484 |
| 860 | 172 | 30.562 | 31.919 |
| 861 | 172.2 | 30.437 | 32.026 |
| 862 | 172.4 | 30.61 | 31.807 |
| 863 | 172.6 | 30.372 | 31.552 |
| 864 | 172.8 | 30.377 | 31.944 |
| 865 | 173 | 30.3 | 32.049 |
| 866 | 173.2 | 30.713 | 32.259 |
| 867 | 173.4 | 30.723 | 32.726 |
| 868 | 173.6 | 30.429 | 33.038 |
| 869 | 173.8 | 30.356 | 33.562 |
| 870 | 174 | 30.826 | 35.124 |
| 871 | 174.2 | 30.312 | 35.953 |
| 872 | 174.4 | 30.47 | 36.918 |
| 873 | 174.6 | 30.165 | 37.713 |
| 874 | 174.8 | 29.882 | 38.131 |
| 875 | 175 | 30.528 | 38.389 |
| 876 | 175.2 | 30.444 | 37.26 |
| 877 | 175.4 | 30.571 | 36.034 |
| 878 | 175.6 | 30.349 | 35.135 |
| 879 | 175.8 | 30.633 | 34.918 |
| 880 | 176 | 30.411 | 34.755 |
| 881 | 176.2 | 30.474 | 34.038 |
| 882 | 176.4 | 30.625 | 33.79 |
| 883 | 176.6 | 30.571 | 33.303 |
| 884 | 176.8 | 31.117 | 31.839 |
| 885 | 177 | 30.6 | 31.768 |
| 886 | 177.2 | 30.607 | 31.559 |
| 887 | 177.4 | 30.575 | 31.456 |
| 888 | 177.6 | 30.707 | 31.899 |
| 889 | 177.8 | 30.107 | 32.162 |
| 890 | 178 | 30.5 | 32.979 |
| 891 | 178.2 | 30.866 | 33.519 |
| 892 | 178.4 | 30.654 | 34.528 |
| 893 | 178.6 | 30.724 | 35.744 |
| 894 | 178.8 | 30.811 | 37.185 |
| 895 | 179 | 30.409 | 37.821 |
| 896 | 179.2 | 30.314 | 37.628 |
| 897 | 179.4 | 30.394 | 35.636 |
| 898 | 179.6 | 30.751 | 33.333 |
| 899 | 179.8 | 30.263 | 32.051 |
| 900 | 180 | 30.641 | 31.863 |
| 901 | 180.2 | 30.491 | 31.805 |
| 902 | 180.4 | 30.429 | 31.858 |
| 903 | 180.6 | 29.969 | 32.694 |
| 904 | 180.8 | 30.158 | 32.962 |
| 905 | 181 | 29.682 | 33.509 |
| 906 | 181.2 | 30.088 | 34.147 |
| 907 | 181.4 | 30.032 | 34.903 |
| 908 | 181.6 | 30.361 | 35.156 |
| 909 | 181.8 | 30.696 | 35.757 |
| 910 | 182 | 30.762 | 36.378 |
| 911 | 182.2 | 30.681 | 36.662 |
| 912 | 182.4 | 30.468 | 37.086 |
| 913 | 182.6 | 30.512 | 37.796 |
| 914 | 182.8 | 30.124 | 38.121 |
| 915 | 183 | 30.503 | 37.89 |
| 916 | 183.2 | 30.191 | 37.068 |
| 917 | 183.4 | 30.958 | 35.61 |
| 918 | 183.6 | 30.437 | 34.512 |
| 919 | 183.8 | 29.848 | 33.032 |
| 920 | 184 | 30.164 | 32.176 |
| 921 | 184.2 | 30.302 | 32.167 |
| 922 | 184.4 | 30.207 | 31.845 |
| 923 | 184.6 | 30.665 | 31.623 |
| 924 | 184.8 | 30.375 | 31.794 |
| 925 | 185 | 30.522 | 32.049 |
| 926 | 185.2 | 30.468 | 32.52 |
| 927 | 185.4 | 30.605 | 33.182 |
| 928 | 185.6 | 30.76 | 33.563 |
| 929 | 185.8 | 30.65 | 33.928 |
| 930 | 186 | 30.119 | 34.849 |
| 931 | 186.2 | 30.929 | 36.016 |
| 932 | 186.4 | 30.704 | 36.851 |
| 933 | 186.6 | 30.885 | 37.314 |
| 934 | 186.8 | 30.294 | 37.516 |
| 935 | 187 | 30.761 | 38.092 |
| 936 | 187.2 | 30.486 | 38.106 |
| 937 | 187.4 | 30.487 | 36.415 |
| 938 | 187.6 | 30.469 | 35.264 |
| 939 | 187.8 | 30.686 | 33.513 |
| 940 | 188 | 30.778 | 31.902 |
| 941 | 188.2 | 30.608 | 31.874 |
| 942 | 188.4 | 30.89 | 31.644 |
| 943 | 188.6 | 30.889 | 31.762 |
| 944 | 188.8 | 30.664 | 32.301 |
| 945 | 189 | 30.469 | 32.868 |
| 946 | 189.2 | 30.263 | 33.595 |
| 947 | 189.4 | 30.368 | 34.16 |
| 948 | 189.6 | 30.396 | 34.187 |
| 949 | 189.8 | 30.98 | 34.778 |
| 950 | 190 | 30.404 | 35.606 |
| 951 | 190.2 | 30.497 | 35.99 |
| 952 | 190.4 | 30.429 | 36.625 |
| 953 | 190.6 | 30.789 | 37.198 |
| 954 | 190.8 | 30.705 | 37.52 |
| 955 | 191 | 30.863 | 37.447 |
| 956 | 191.2 | 31.076 | 37.991 |
| 957 | 191.4 | 31.123 | 38.138 |
| 958 | 191.6 | 30.92 | 37.237 |
| 959 | 191.8 | 30.763 | 35.332 |
| 960 | 192 | 31.138 | 33.482 |
| 961 | 192.2 | 30.818 | 32.324 |
| 962 | 192.4 | 30.954 | 32.003 |
| 963 | 192.6 | 31.112 | 31.35 |
| 964 | 192.8 | 30.843 | 31.523 |
| 965 | 193 | 30.687 | 31.386 |
| 966 | 193.2 | 30.394 | 31.672 |
| 967 | 193.4 | 30.537 | 31.411 |
| 968 | 193.6 | 30.503 | 31.855 |
| 969 | 193.8 | 30.875 | 32.099 |
| 970 | 194 | 30.695 | 32.435 |
| 971 | 194.2 | 30.94 | 33.172 |
| 972 | 194.4 | 30.666 | 34.111 |
| 973 | 194.6 | 30.648 | 35.435 |
| 974 | 194.8 | 30.368 | 36.209 |
| 975 | 195 | 30.51 | 37.388 |
| 976 | 195.2 | 30.876 | 37.615 |
| 977 | 195.4 | 30.231 | 37.525 |
| 978 | 195.6 | 30.353 | 36.174 |
| 979 | 195.8 | 30.409 | 32.914 |
| 980 | 196 | 30.414 | 31.499 |
| 981 | 196.2 | 29.712 | 31.039 |
| 982 | 196.4 | 30.237 | 31.248 |
| 983 | 196.6 | 30.073 | 31.77 |
| 984 | 196.8 | 30.581 | 32.464 |
| 985 | 197 | 30.322 | 33.505 |
| 986 | 197.2 | 30.391 | 34.429 |
| 987 | 197.4 | 30.44 | 35.641 |
| 988 | 197.6 | 30.434 | 36.712 |
| 989 | 197.8 | 30.417 | 37.629 |
| 990 | 198 | 30.736 | 38.291 |
| 991 | 198.2 | 30.77 | 38.43 |
| 992 | 198.4 | 30.176 | 36.955 |
| 993 | 198.6 | 30.464 | 34.987 |
| 994 | 198.8 | 30.603 | 33.115 |
| 995 | 199 | 30.511 | 32.122 |
| 996 | 199.2 | 30.513 | 31.834 |
| 997 | 199.4 | 30.225 | 31.677 |
| 998 | 199.6 | 30.462 | 31.917 |
| 999 | 199.8 | 30.487 | 31.859 |
| 1000 | 200 | 30.643 | 32.189 |
| 1001 | 200.2 | 30.578 | 32.128 |
