# Supplementary material for: Immunoinformatics and reverse vaccinology design of self-amplifying RNA vaccine against marburg virus with structural dynamics and immune simulation analysis
Source: PLoS One. 2026 Oct 23;21(10):e0361136. doi: 10.1371/journal.pone.0361136 (PMC13693908; doi:10.1371/journal.pone.0361136)
Supplement: Supplementary file 6 [file pone.0361136.s005.docx]

**Protein H Bond**

| **Frame** | **Time(ns)** | **MB-mVax-TLR2** | **MB-mVax-TLR4** |
| --- | --- | --- | --- |
| 0 | 0 | 510 | 547 |
| 1 | 0.2 | 465 | 507 |
| 2 | 0.4 | 473 | 469 |
| 3 | 0.6 | 469 | 498 |
| 4 | 0.8 | 479 | 478 |
| 5 | 1 | 451 | 490 |
| 6 | 1.2 | 446 | 472 |
| 7 | 1.4 | 457 | 478 |
| 8 | 1.6 | 458 | 478 |
| 9 | 1.8 | 465 | 484 |
| 10 | 2 | 457 | 502 |
| 11 | 2.2 | 468 | 485 |
| 12 | 2.4 | 441 | 494 |
| 13 | 2.6 | 442 | 497 |
| 14 | 2.8 | 446 | 511 |
| 15 | 3 | 448 | 492 |
| 16 | 3.2 | 467 | 492 |
| 17 | 3.4 | 453 | 475 |
| 18 | 3.6 | 466 | 502 |
| 19 | 3.8 | 464 | 482 |
| 20 | 4 | 457 | 487 |
| 21 | 4.2 | 467 | 490 |
| 22 | 4.4 | 456 | 489 |
| 23 | 4.6 | 468 | 487 |
| 24 | 4.8 | 461 | 479 |
| 25 | 5 | 472 | 474 |
| 26 | 5.2 | 458 | 487 |
| 27 | 5.4 | 468 | 494 |
| 28 | 5.6 | 454 | 495 |
| 29 | 5.8 | 453 | 467 |
| 30 | 6 | 452 | 499 |
| 31 | 6.2 | 456 | 490 |
| 32 | 6.4 | 464 | 490 |
| 33 | 6.6 | 466 | 502 |
| 34 | 6.8 | 473 | 496 |
| 35 | 7 | 454 | 487 |
| 36 | 7.2 | 468 | 491 |
| 37 | 7.4 | 462 | 477 |
| 38 | 7.6 | 462 | 482 |
| 39 | 7.8 | 475 | 483 |
| 40 | 8 | 460 | 484 |
| 41 | 8.2 | 469 | 475 |
| 42 | 8.4 | 483 | 483 |
| 43 | 8.6 | 463 | 489 |
| 44 | 8.8 | 469 | 472 |
| 45 | 9 | 474 | 489 |
| 46 | 9.2 | 455 | 494 |
| 47 | 9.4 | 442 | 477 |
| 48 | 9.6 | 455 | 509 |
| 49 | 9.8 | 472 | 490 |
| 50 | 10 | 465 | 494 |
| 51 | 10.2 | 458 | 479 |
| 52 | 10.4 | 478 | 477 |
| 53 | 10.6 | 457 | 471 |
| 54 | 10.8 | 478 | 480 |
| 55 | 11 | 471 | 489 |
| 56 | 11.2 | 485 | 473 |
| 57 | 11.4 | 489 | 490 |
| 58 | 11.6 | 480 | 468 |
| 59 | 11.8 | 473 | 492 |
| 60 | 12 | 457 | 479 |
| 61 | 12.2 | 454 | 485 |
| 62 | 12.4 | 446 | 485 |
| 63 | 12.6 | 471 | 488 |
| 64 | 12.8 | 469 | 482 |
| 65 | 13 | 462 | 508 |
| 66 | 13.2 | 469 | 481 |
| 67 | 13.4 | 458 | 500 |
| 68 | 13.6 | 464 | 488 |
| 69 | 13.8 | 474 | 503 |
| 70 | 14 | 481 | 490 |
| 71 | 14.2 | 466 | 496 |
| 72 | 14.4 | 466 | 484 |
| 73 | 14.6 | 465 | 495 |
| 74 | 14.8 | 471 | 492 |
| 75 | 15 | 453 | 465 |
| 76 | 15.2 | 466 | 480 |
| 77 | 15.4 | 446 | 482 |
| 78 | 15.6 | 474 | 489 |
| 79 | 15.8 | 472 | 475 |
| 80 | 16 | 464 | 490 |
| 81 | 16.2 | 474 | 493 |
| 82 | 16.4 | 478 | 481 |
| 83 | 16.6 | 471 | 486 |
| 84 | 16.8 | 475 | 502 |
| 85 | 17 | 474 | 480 |
| 86 | 17.2 | 467 | 483 |
| 87 | 17.4 | 467 | 492 |
| 88 | 17.6 | 471 | 504 |
| 89 | 17.8 | 461 | 497 |
| 90 | 18 | 466 | 475 |
| 91 | 18.2 | 461 | 472 |
| 92 | 18.4 | 465 | 479 |
| 93 | 18.6 | 468 | 494 |
| 94 | 18.8 | 468 | 465 |
| 95 | 19 | 472 | 470 |
| 96 | 19.2 | 466 | 500 |
| 97 | 19.4 | 472 | 481 |
| 98 | 19.6 | 458 | 489 |
| 99 | 19.8 | 471 | 474 |
| 100 | 20 | 468 | 473 |
| 101 | 20.2 | 458 | 485 |
| 102 | 20.4 | 468 | 462 |
| 103 | 20.6 | 479 | 480 |
| 104 | 20.8 | 461 | 484 |
| 105 | 21 | 474 | 474 |
| 106 | 21.2 | 463 | 474 |
| 107 | 21.4 | 471 | 488 |
| 108 | 21.6 | 485 | 483 |
| 109 | 21.8 | 466 | 475 |
| 110 | 22 | 473 | 474 |
| 111 | 22.2 | 464 | 462 |
| 112 | 22.4 | 470 | 471 |
| 113 | 22.6 | 448 | 481 |
| 114 | 22.8 | 476 | 476 |
| 115 | 23 | 453 | 473 |
| 116 | 23.2 | 459 | 469 |
| 117 | 23.4 | 465 | 485 |
| 118 | 23.6 | 453 | 485 |
| 119 | 23.8 | 455 | 486 |
| 120 | 24 | 475 | 493 |
| 121 | 24.2 | 460 | 467 |
| 122 | 24.4 | 467 | 479 |
| 123 | 24.6 | 473 | 478 |
| 124 | 24.8 | 475 | 478 |
| 125 | 25 | 466 | 479 |
| 126 | 25.2 | 456 | 496 |
| 127 | 25.4 | 456 | 487 |
| 128 | 25.6 | 455 | 487 |
| 129 | 25.8 | 461 | 477 |
| 130 | 26 | 446 | 505 |
| 131 | 26.2 | 454 | 491 |
| 132 | 26.4 | 462 | 490 |
| 133 | 26.6 | 456 | 494 |
| 134 | 26.8 | 455 | 472 |
| 135 | 27 | 452 | 483 |
| 136 | 27.2 | 469 | 489 |
| 137 | 27.4 | 466 | 496 |
| 138 | 27.6 | 468 | 477 |
| 139 | 27.8 | 479 | 489 |
| 140 | 28 | 459 | 490 |
| 141 | 28.2 | 478 | 488 |
| 142 | 28.4 | 477 | 478 |
| 143 | 28.6 | 483 | 477 |
| 144 | 28.8 | 479 | 472 |
| 145 | 29 | 482 | 490 |
| 146 | 29.2 | 483 | 486 |
| 147 | 29.4 | 478 | 470 |
| 148 | 29.6 | 466 | 471 |
| 149 | 29.8 | 472 | 483 |
| 150 | 30 | 471 | 500 |
| 151 | 30.2 | 484 | 476 |
| 152 | 30.4 | 476 | 485 |
| 153 | 30.6 | 493 | 474 |
| 154 | 30.8 | 477 | 487 |
| 155 | 31 | 494 | 494 |
| 156 | 31.2 | 478 | 500 |
| 157 | 31.4 | 470 | 482 |
| 158 | 31.6 | 482 | 490 |
| 159 | 31.8 | 493 | 483 |
| 160 | 32 | 477 | 479 |
| 161 | 32.2 | 478 | 477 |
| 162 | 32.4 | 483 | 494 |
| 163 | 32.6 | 491 | 493 |
| 164 | 32.8 | 504 | 471 |
| 165 | 33 | 500 | 486 |
| 166 | 33.2 | 483 | 490 |
| 167 | 33.4 | 467 | 476 |
| 168 | 33.6 | 461 | 480 |
| 169 | 33.8 | 464 | 480 |
| 170 | 34 | 475 | 459 |
| 171 | 34.2 | 459 | 488 |
| 172 | 34.4 | 461 | 482 |
| 173 | 34.6 | 470 | 482 |
| 174 | 34.8 | 448 | 496 |
| 175 | 35 | 476 | 482 |
| 176 | 35.2 | 481 | 469 |
| 177 | 35.4 | 487 | 480 |
| 178 | 35.6 | 456 | 472 |
| 179 | 35.8 | 466 | 481 |
| 180 | 36 | 473 | 488 |
| 181 | 36.2 | 478 | 480 |
| 182 | 36.4 | 468 | 482 |
| 183 | 36.6 | 469 | 477 |
| 184 | 36.8 | 454 | 481 |
| 185 | 37 | 480 | 461 |
| 186 | 37.2 | 467 | 469 |
| 187 | 37.4 | 459 | 482 |
| 188 | 37.6 | 472 | 465 |
| 189 | 37.8 | 466 | 475 |
| 190 | 38 | 456 | 476 |
| 191 | 38.2 | 471 | 500 |
| 192 | 38.4 | 465 | 481 |
| 193 | 38.6 | 456 | 475 |
| 194 | 38.8 | 466 | 475 |
| 195 | 39 | 488 | 492 |
| 196 | 39.2 | 480 | 461 |
| 197 | 39.4 | 465 | 482 |
| 198 | 39.6 | 454 | 465 |
| 199 | 39.8 | 473 | 465 |
| 200 | 40 | 471 | 467 |
| 201 | 40.2 | 460 | 462 |
| 202 | 40.4 | 464 | 464 |
| 203 | 40.6 | 459 | 470 |
| 204 | 40.8 | 453 | 492 |
| 205 | 41 | 468 | 480 |
| 206 | 41.2 | 461 | 462 |
| 207 | 41.4 | 463 | 483 |
| 208 | 41.6 | 462 | 477 |
| 209 | 41.8 | 460 | 471 |
| 210 | 42 | 460 | 482 |
| 211 | 42.2 | 458 | 463 |
| 212 | 42.4 | 475 | 472 |
| 213 | 42.6 | 452 | 479 |
| 214 | 42.8 | 462 | 462 |
| 215 | 43 | 462 | 463 |
| 216 | 43.2 | 459 | 455 |
| 217 | 43.4 | 453 | 465 |
| 218 | 43.6 | 469 | 465 |
| 219 | 43.8 | 461 | 457 |
| 220 | 44 | 469 | 468 |
| 221 | 44.2 | 468 | 469 |
| 222 | 44.4 | 463 | 467 |
| 223 | 44.6 | 442 | 475 |
| 224 | 44.8 | 481 | 465 |
| 225 | 45 | 461 | 469 |
| 226 | 45.2 | 471 | 474 |
| 227 | 45.4 | 472 | 480 |
| 228 | 45.6 | 459 | 475 |
| 229 | 45.8 | 458 | 476 |
| 230 | 46 | 465 | 482 |
| 231 | 46.2 | 470 | 476 |
| 232 | 46.4 | 467 | 477 |
| 233 | 46.6 | 466 | 467 |
| 234 | 46.8 | 470 | 476 |
| 235 | 47 | 461 | 471 |
| 236 | 47.2 | 466 | 484 |
| 237 | 47.4 | 465 | 464 |
| 238 | 47.6 | 468 | 485 |
| 239 | 47.8 | 481 | 481 |
| 240 | 48 | 466 | 478 |
| 241 | 48.2 | 472 | 475 |
| 242 | 48.4 | 462 | 468 |
| 243 | 48.6 | 475 | 478 |
| 244 | 48.8 | 448 | 477 |
| 245 | 49 | 461 | 475 |
| 246 | 49.2 | 475 | 476 |
| 247 | 49.4 | 463 | 481 |
| 248 | 49.6 | 477 | 483 |
| 249 | 49.8 | 462 | 463 |
| 250 | 50 | 471 | 474 |
| 251 | 50.2 | 470 | 485 |
| 252 | 50.4 | 467 | 497 |
| 253 | 50.6 | 471 | 478 |
| 254 | 50.8 | 457 | 492 |
| 255 | 51 | 470 | 487 |
| 256 | 51.2 | 485 | 493 |
| 257 | 51.4 | 459 | 485 |
| 258 | 51.6 | 459 | 485 |
| 259 | 51.8 | 466 | 486 |
| 260 | 52 | 467 | 461 |
| 261 | 52.2 | 478 | 483 |
| 262 | 52.4 | 462 | 479 |
| 263 | 52.6 | 473 | 474 |
| 264 | 52.8 | 457 | 466 |
| 265 | 53 | 471 | 463 |
| 266 | 53.2 | 463 | 466 |
| 267 | 53.4 | 473 | 474 |
| 268 | 53.6 | 463 | 487 |
| 269 | 53.8 | 445 | 470 |
| 270 | 54 | 449 | 490 |
| 271 | 54.2 | 472 | 486 |
| 272 | 54.4 | 472 | 466 |
| 273 | 54.6 | 460 | 477 |
| 274 | 54.8 | 477 | 488 |
| 275 | 55 | 472 | 480 |
| 276 | 55.2 | 469 | 470 |
| 277 | 55.4 | 460 | 481 |
| 278 | 55.6 | 466 | 483 |
| 279 | 55.8 | 460 | 487 |
| 280 | 56 | 460 | 475 |
| 281 | 56.2 | 473 | 469 |
| 282 | 56.4 | 451 | 485 |
| 283 | 56.6 | 465 | 475 |
| 284 | 56.8 | 469 | 484 |
| 285 | 57 | 465 | 483 |
| 286 | 57.2 | 479 | 485 |
| 287 | 57.4 | 481 | 499 |
| 288 | 57.6 | 465 | 479 |
| 289 | 57.8 | 477 | 482 |
| 290 | 58 | 468 | 480 |
| 291 | 58.2 | 461 | 476 |
| 292 | 58.4 | 471 | 478 |
| 293 | 58.6 | 484 | 490 |
| 294 | 58.8 | 451 | 481 |
| 295 | 59 | 474 | 483 |
| 296 | 59.2 | 447 | 482 |
| 297 | 59.4 | 464 | 472 |
| 298 | 59.6 | 468 | 480 |
| 299 | 59.8 | 474 | 486 |
| 300 | 60 | 473 | 486 |
| 301 | 60.2 | 479 | 483 |
| 302 | 60.4 | 467 | 486 |
| 303 | 60.6 | 487 | 470 |
| 304 | 60.8 | 473 | 474 |
| 305 | 61 | 479 | 491 |
| 306 | 61.2 | 460 | 490 |
| 307 | 61.4 | 467 | 486 |
| 308 | 61.6 | 475 | 471 |
| 309 | 61.8 | 469 | 484 |
| 310 | 62 | 464 | 469 |
| 311 | 62.2 | 487 | 486 |
| 312 | 62.4 | 490 | 472 |
| 313 | 62.6 | 485 | 466 |
| 314 | 62.8 | 478 | 466 |
| 315 | 63 | 476 | 470 |
| 316 | 63.2 | 475 | 474 |
| 317 | 63.4 | 484 | 472 |
| 318 | 63.6 | 476 | 462 |
| 319 | 63.8 | 492 | 475 |
| 320 | 64 | 497 | 480 |
| 321 | 64.2 | 463 | 471 |
| 322 | 64.4 | 457 | 483 |
| 323 | 64.6 | 481 | 483 |
| 324 | 64.8 | 472 | 490 |
| 325 | 65 | 480 | 473 |
| 326 | 65.2 | 466 | 470 |
| 327 | 65.4 | 479 | 469 |
| 328 | 65.6 | 466 | 483 |
| 329 | 65.8 | 467 | 474 |
| 330 | 66 | 475 | 476 |
| 331 | 66.2 | 477 | 477 |
| 332 | 66.4 | 471 | 477 |
| 333 | 66.6 | 471 | 496 |
| 334 | 66.8 | 477 | 485 |
| 335 | 67 | 459 | 464 |
| 336 | 67.2 | 482 | 476 |
| 337 | 67.4 | 474 | 493 |
| 338 | 67.6 | 462 | 467 |
| 339 | 67.8 | 453 | 478 |
| 340 | 68 | 473 | 478 |
| 341 | 68.2 | 470 | 476 |
| 342 | 68.4 | 475 | 481 |
| 343 | 68.6 | 473 | 469 |
| 344 | 68.8 | 472 | 470 |
| 345 | 69 | 486 | 463 |
| 346 | 69.2 | 476 | 488 |
| 347 | 69.4 | 469 | 478 |
| 348 | 69.6 | 484 | 475 |
| 349 | 69.8 | 463 | 481 |
| 350 | 70 | 464 | 475 |
| 351 | 70.2 | 469 | 468 |
| 352 | 70.4 | 475 | 477 |
| 353 | 70.6 | 462 | 482 |
| 354 | 70.8 | 454 | 480 |
| 355 | 71 | 461 | 463 |
| 356 | 71.2 | 466 | 484 |
| 357 | 71.4 | 480 | 488 |
| 358 | 71.6 | 476 | 482 |
| 359 | 71.8 | 459 | 492 |
| 360 | 72 | 477 | 487 |
| 361 | 72.2 | 446 | 477 |
| 362 | 72.4 | 466 | 478 |
| 363 | 72.6 | 460 | 465 |
| 364 | 72.8 | 458 | 469 |
| 365 | 73 | 465 | 481 |
| 366 | 73.2 | 470 | 479 |
| 367 | 73.4 | 453 | 467 |
| 368 | 73.6 | 467 | 482 |
| 369 | 73.8 | 471 | 487 |
| 370 | 74 | 449 | 482 |
| 371 | 74.2 | 463 | 493 |
| 372 | 74.4 | 451 | 485 |
| 373 | 74.6 | 452 | 480 |
| 374 | 74.8 | 447 | 477 |
| 375 | 75 | 457 | 476 |
| 376 | 75.2 | 464 | 487 |
| 377 | 75.4 | 453 | 458 |
| 378 | 75.6 | 457 | 479 |
| 379 | 75.8 | 472 | 495 |
| 380 | 76 | 462 | 478 |
| 381 | 76.2 | 461 | 512 |
| 382 | 76.4 | 446 | 505 |
| 383 | 76.6 | 460 | 492 |
| 384 | 76.8 | 480 | 476 |
| 385 | 77 | 468 | 513 |
| 386 | 77.2 | 489 | 486 |
| 387 | 77.4 | 464 | 488 |
| 388 | 77.6 | 475 | 485 |
| 389 | 77.8 | 456 | 486 |
| 390 | 78 | 472 | 491 |
| 391 | 78.2 | 465 | 476 |
| 392 | 78.4 | 475 | 485 |
| 393 | 78.6 | 484 | 496 |
| 394 | 78.8 | 460 | 484 |
| 395 | 79 | 457 | 472 |
| 396 | 79.2 | 456 | 488 |
| 397 | 79.4 | 444 | 473 |
| 398 | 79.6 | 453 | 479 |
| 399 | 79.8 | 476 | 468 |
| 400 | 80 | 470 | 506 |
| 401 | 80.2 | 456 | 495 |
| 402 | 80.4 | 467 | 488 |
| 403 | 80.6 | 468 | 486 |
| 404 | 80.8 | 461 | 500 |
| 405 | 81 | 461 | 497 |
| 406 | 81.2 | 445 | 490 |
| 407 | 81.4 | 461 | 483 |
| 408 | 81.6 | 479 | 491 |
| 409 | 81.8 | 463 | 506 |
| 410 | 82 | 456 | 484 |
| 411 | 82.2 | 451 | 484 |
| 412 | 82.4 | 468 | 490 |
| 413 | 82.6 | 467 | 476 |
| 414 | 82.8 | 451 | 485 |
| 415 | 83 | 469 | 506 |
| 416 | 83.2 | 455 | 495 |
| 417 | 83.4 | 464 | 494 |
| 418 | 83.6 | 454 | 496 |
| 419 | 83.8 | 472 | 496 |
| 420 | 84 | 446 | 490 |
| 421 | 84.2 | 472 | 500 |
| 422 | 84.4 | 465 | 468 |
| 423 | 84.6 | 445 | 485 |
| 424 | 84.8 | 469 | 492 |
| 425 | 85 | 457 | 489 |
| 426 | 85.2 | 448 | 502 |
| 427 | 85.4 | 462 | 490 |
| 428 | 85.6 | 455 | 481 |
| 429 | 85.8 | 463 | 484 |
| 430 | 86 | 459 | 494 |
| 431 | 86.2 | 470 | 501 |
| 432 | 86.4 | 459 | 501 |
| 433 | 86.6 | 480 | 483 |
| 434 | 86.8 | 453 | 484 |
| 435 | 87 | 456 | 487 |
| 436 | 87.2 | 463 | 483 |
| 437 | 87.4 | 453 | 471 |
| 438 | 87.6 | 463 | 491 |
| 439 | 87.8 | 454 | 480 |
| 440 | 88 | 464 | 493 |
| 441 | 88.2 | 447 | 480 |
| 442 | 88.4 | 464 | 497 |
| 443 | 88.6 | 459 | 488 |
| 444 | 88.8 | 470 | 497 |
| 445 | 89 | 435 | 482 |
| 446 | 89.2 | 451 | 501 |
| 447 | 89.4 | 448 | 489 |
| 448 | 89.6 | 466 | 496 |
| 449 | 89.8 | 466 | 507 |
| 450 | 90 | 465 | 488 |
| 451 | 90.2 | 450 | 508 |
| 452 | 90.4 | 468 | 498 |
| 453 | 90.6 | 458 | 500 |
| 454 | 90.8 | 457 | 499 |
| 455 | 91 | 459 | 497 |
| 456 | 91.2 | 474 | 474 |
| 457 | 91.4 | 447 | 487 |
| 458 | 91.6 | 474 | 495 |
| 459 | 91.8 | 447 | 498 |
| 460 | 92 | 466 | 489 |
| 461 | 92.2 | 458 | 473 |
| 462 | 92.4 | 461 | 493 |
| 463 | 92.6 | 455 | 502 |
| 464 | 92.8 | 449 | 483 |
| 465 | 93 | 480 | 476 |
| 466 | 93.2 | 457 | 510 |
| 467 | 93.4 | 445 | 499 |
| 468 | 93.6 | 457 | 495 |
| 469 | 93.8 | 456 | 494 |
| 470 | 94 | 460 | 509 |
| 471 | 94.2 | 456 | 487 |
| 472 | 94.4 | 445 | 494 |
| 473 | 94.6 | 451 | 492 |
| 474 | 94.8 | 461 | 486 |
| 475 | 95 | 452 | 485 |
| 476 | 95.2 | 482 | 486 |
| 477 | 95.4 | 449 | 499 |
| 478 | 95.6 | 455 | 495 |
| 479 | 95.8 | 454 | 480 |
| 480 | 96 | 461 | 501 |
| 481 | 96.2 | 471 | 481 |
| 482 | 96.4 | 457 | 483 |
| 483 | 96.6 | 470 | 505 |
| 484 | 96.8 | 461 | 503 |
| 485 | 97 | 465 | 498 |
| 486 | 97.2 | 440 | 492 |
| 487 | 97.4 | 469 | 507 |
| 488 | 97.6 | 460 | 498 |
| 489 | 97.8 | 458 | 492 |
| 490 | 98 | 467 | 498 |
| 491 | 98.2 | 477 | 487 |
| 492 | 98.4 | 467 | 495 |
| 493 | 98.6 | 470 | 486 |
| 494 | 98.8 | 454 | 487 |
| 495 | 99 | 457 | 475 |
| 496 | 99.2 | 444 | 483 |
| 497 | 99.4 | 469 | 481 |
| 498 | 99.6 | 463 | 466 |
| 499 | 99.8 | 476 | 498 |
| 500 | 100 | 474 | 470 |
| 501 | 100.2 | 461 | 474 |
| 502 | 100.4 | 471 | 498 |
| 503 | 100.6 | 465 | 481 |
| 504 | 100.8 | 466 | 478 |
| 505 | 101 | 468 | 489 |
| 506 | 101.2 | 463 | 520 |
| 507 | 101.4 | 466 | 489 |
| 508 | 101.6 | 481 | 503 |
| 509 | 101.8 | 469 | 496 |
| 510 | 102 | 473 | 466 |
| 511 | 102.2 | 488 | 488 |
| 512 | 102.4 | 469 | 472 |
| 513 | 102.6 | 460 | 475 |
| 514 | 102.8 | 468 | 491 |
| 515 | 103 | 460 | 496 |
| 516 | 103.2 | 466 | 496 |
| 517 | 103.4 | 471 | 480 |
| 518 | 103.6 | 459 | 468 |
| 519 | 103.8 | 469 | 461 |
| 520 | 104 | 459 | 472 |
| 521 | 104.2 | 454 | 477 |
| 522 | 104.4 | 451 | 468 |
| 523 | 104.6 | 453 | 481 |
| 524 | 104.8 | 458 | 463 |
| 525 | 105 | 454 | 472 |
| 526 | 105.2 | 447 | 470 |
| 527 | 105.4 | 456 | 479 |
| 528 | 105.6 | 449 | 494 |
| 529 | 105.8 | 458 | 492 |
| 530 | 106 | 467 | 474 |
| 531 | 106.2 | 458 | 478 |
| 532 | 106.4 | 446 | 491 |
| 533 | 106.6 | 456 | 488 |
| 534 | 106.8 | 462 | 485 |
| 535 | 107 | 454 | 489 |
| 536 | 107.2 | 466 | 493 |
| 537 | 107.4 | 469 | 481 |
| 538 | 107.6 | 471 | 482 |
| 539 | 107.8 | 458 | 485 |
| 540 | 108 | 472 | 499 |
| 541 | 108.2 | 460 | 505 |
| 542 | 108.4 | 476 | 485 |
| 543 | 108.6 | 466 | 485 |
| 544 | 108.8 | 489 | 486 |
| 545 | 109 | 480 | 465 |
| 546 | 109.2 | 477 | 458 |
| 547 | 109.4 | 454 | 483 |
| 548 | 109.6 | 474 | 475 |
| 549 | 109.8 | 459 | 502 |
| 550 | 110 | 462 | 499 |
| 551 | 110.2 | 458 | 489 |
| 552 | 110.4 | 444 | 483 |
| 553 | 110.6 | 470 | 484 |
| 554 | 110.8 | 462 | 475 |
| 555 | 111 | 464 | 473 |
| 556 | 111.2 | 453 | 480 |
| 557 | 111.4 | 474 | 475 |
| 558 | 111.6 | 465 | 491 |
| 559 | 111.8 | 474 | 479 |
| 560 | 112 | 472 | 492 |
| 561 | 112.2 | 472 | 483 |
| 562 | 112.4 | 461 | 484 |
| 563 | 112.6 | 467 | 485 |
| 564 | 112.8 | 467 | 500 |
| 565 | 113 | 478 | 483 |
| 566 | 113.2 | 456 | 499 |
| 567 | 113.4 | 477 | 489 |
| 568 | 113.6 | 481 | 475 |
| 569 | 113.8 | 474 | 502 |
| 570 | 114 | 470 | 505 |
| 571 | 114.2 | 459 | 479 |
| 572 | 114.4 | 453 | 489 |
| 573 | 114.6 | 452 | 488 |
| 574 | 114.8 | 449 | 498 |
| 575 | 115 | 469 | 487 |
| 576 | 115.2 | 453 | 493 |
| 577 | 115.4 | 469 | 491 |
| 578 | 115.6 | 455 | 479 |
| 579 | 115.8 | 463 | 496 |
| 580 | 116 | 463 | 484 |
| 581 | 116.2 | 454 | 492 |
| 582 | 116.4 | 457 | 484 |
| 583 | 116.6 | 457 | 475 |
| 584 | 116.8 | 451 | 483 |
| 585 | 117 | 461 | 476 |
| 586 | 117.2 | 464 | 478 |
| 587 | 117.4 | 466 | 489 |
| 588 | 117.6 | 476 | 480 |
| 589 | 117.8 | 480 | 486 |
| 590 | 118 | 464 | 467 |
| 591 | 118.2 | 474 | 476 |
| 592 | 118.4 | 466 | 480 |
| 593 | 118.6 | 449 | 478 |
| 594 | 118.8 | 459 | 494 |
| 595 | 119 | 464 | 480 |
| 596 | 119.2 | 464 | 490 |
| 597 | 119.4 | 473 | 496 |
| 598 | 119.6 | 471 | 484 |
| 599 | 119.8 | 455 | 476 |
| 600 | 120 | 465 | 482 |
| 601 | 120.2 | 464 | 465 |
| 602 | 120.4 | 466 | 476 |
| 603 | 120.6 | 448 | 470 |
| 604 | 120.8 | 447 | 483 |
| 605 | 121 | 455 | 501 |
| 606 | 121.2 | 465 | 503 |
| 607 | 121.4 | 472 | 485 |
| 608 | 121.6 | 463 | 483 |
| 609 | 121.8 | 472 | 479 |
| 610 | 122 | 462 | 496 |
| 611 | 122.2 | 474 | 505 |
| 612 | 122.4 | 458 | 485 |
| 613 | 122.6 | 457 | 507 |
| 614 | 122.8 | 474 | 496 |
| 615 | 123 | 468 | 485 |
| 616 | 123.2 | 484 | 489 |
| 617 | 123.4 | 471 | 484 |
| 618 | 123.6 | 471 | 487 |
| 619 | 123.8 | 475 | 480 |
| 620 | 124 | 491 | 493 |
| 621 | 124.2 | 484 | 492 |
| 622 | 124.4 | 476 | 494 |
| 623 | 124.6 | 469 | 476 |
| 624 | 124.8 | 479 | 489 |
| 625 | 125 | 495 | 498 |
| 626 | 125.2 | 472 | 490 |
| 627 | 125.4 | 505 | 499 |
| 628 | 125.6 | 478 | 484 |
| 629 | 125.8 | 469 | 486 |
| 630 | 126 | 477 | 484 |
| 631 | 126.2 | 465 | 479 |
| 632 | 126.4 | 466 | 488 |
| 633 | 126.6 | 472 | 476 |
| 634 | 126.8 | 466 | 494 |
| 635 | 127 | 473 | 484 |
| 636 | 127.2 | 464 | 499 |
| 637 | 127.4 | 471 | 487 |
| 638 | 127.6 | 462 | 490 |
| 639 | 127.8 | 476 | 479 |
| 640 | 128 | 482 | 496 |
| 641 | 128.2 | 477 | 487 |
| 642 | 128.4 | 470 | 497 |
| 643 | 128.6 | 463 | 486 |
| 644 | 128.8 | 459 | 492 |
| 645 | 129 | 460 | 495 |
| 646 | 129.2 | 453 | 487 |
| 647 | 129.4 | 458 | 479 |
| 648 | 129.6 | 447 | 490 |
| 649 | 129.8 | 479 | 495 |
| 650 | 130 | 467 | 476 |
| 651 | 130.2 | 457 | 481 |
| 652 | 130.4 | 469 | 493 |
| 653 | 130.6 | 460 | 499 |
| 654 | 130.8 | 474 | 497 |
| 655 | 131 | 474 | 492 |
| 656 | 131.2 | 460 | 495 |
| 657 | 131.4 | 466 | 498 |
| 658 | 131.6 | 489 | 485 |
| 659 | 131.8 | 479 | 486 |
| 660 | 132 | 486 | 478 |
| 661 | 132.2 | 485 | 485 |
| 662 | 132.4 | 482 | 487 |
| 663 | 132.6 | 480 | 491 |
| 664 | 132.8 | 487 | 487 |
| 665 | 133 | 477 | 478 |
| 666 | 133.2 | 481 | 468 |
| 667 | 133.4 | 497 | 478 |
| 668 | 133.6 | 469 | 473 |
| 669 | 133.8 | 487 | 469 |
| 670 | 134 | 469 | 468 |
| 671 | 134.2 | 478 | 475 |
| 672 | 134.4 | 474 | 495 |
| 673 | 134.6 | 480 | 486 |
| 674 | 134.8 | 485 | 493 |
| 675 | 135 | 476 | 474 |
| 676 | 135.2 | 478 | 482 |
| 677 | 135.4 | 468 | 483 |
| 678 | 135.6 | 472 | 494 |
| 679 | 135.8 | 447 | 494 |
| 680 | 136 | 468 | 485 |
| 681 | 136.2 | 461 | 483 |
| 682 | 136.4 | 478 | 492 |
| 683 | 136.6 | 473 | 502 |
| 684 | 136.8 | 483 | 492 |
| 685 | 137 | 467 | 501 |
| 686 | 137.2 | 475 | 480 |
| 687 | 137.4 | 465 | 491 |
| 688 | 137.6 | 471 | 494 |
| 689 | 137.8 | 455 | 496 |
| 690 | 138 | 476 | 479 |
| 691 | 138.2 | 481 | 485 |
| 692 | 138.4 | 489 | 497 |
| 693 | 138.6 | 464 | 479 |
| 694 | 138.8 | 483 | 468 |
| 695 | 139 | 472 | 493 |
| 696 | 139.2 | 485 | 480 |
| 697 | 139.4 | 480 | 488 |
| 698 | 139.6 | 478 | 493 |
| 699 | 139.8 | 468 | 480 |
| 700 | 140 | 469 | 472 |
| 701 | 140.2 | 471 | 471 |
| 702 | 140.4 | 478 | 482 |
| 703 | 140.6 | 473 | 495 |
| 704 | 140.8 | 491 | 471 |
| 705 | 141 | 457 | 489 |
| 706 | 141.2 | 467 | 487 |
| 707 | 141.4 | 479 | 473 |
| 708 | 141.6 | 463 | 478 |
| 709 | 141.8 | 475 | 480 |
| 710 | 142 | 461 | 472 |
| 711 | 142.2 | 467 | 471 |
| 712 | 142.4 | 457 | 461 |
| 713 | 142.6 | 463 | 467 |
| 714 | 142.8 | 457 | 478 |
| 715 | 143 | 473 | 465 |
| 716 | 143.2 | 474 | 475 |
| 717 | 143.4 | 472 | 471 |
| 718 | 143.6 | 483 | 484 |
| 719 | 143.8 | 457 | 473 |
| 720 | 144 | 472 | 482 |
| 721 | 144.2 | 479 | 489 |
| 722 | 144.4 | 483 | 476 |
| 723 | 144.6 | 483 | 472 |
| 724 | 144.8 | 463 | 499 |
| 725 | 145 | 500 | 483 |
| 726 | 145.2 | 472 | 477 |
| 727 | 145.4 | 489 | 482 |
| 728 | 145.6 | 487 | 493 |
| 729 | 145.8 | 472 | 478 |
| 730 | 146 | 479 | 479 |
| 731 | 146.2 | 483 | 459 |
| 732 | 146.4 | 470 | 472 |
| 733 | 146.6 | 485 | 486 |
| 734 | 146.8 | 479 | 490 |
| 735 | 147 | 484 | 478 |
| 736 | 147.2 | 489 | 487 |
| 737 | 147.4 | 484 | 480 |
| 738 | 147.6 | 467 | 475 |
| 739 | 147.8 | 473 | 474 |
| 740 | 148 | 472 | 472 |
| 741 | 148.2 | 471 | 483 |
| 742 | 148.4 | 473 | 468 |
| 743 | 148.6 | 476 | 480 |
| 744 | 148.8 | 467 | 482 |
| 745 | 149 | 482 | 475 |
| 746 | 149.2 | 482 | 470 |
| 747 | 149.4 | 467 | 485 |
| 748 | 149.6 | 462 | 485 |
| 749 | 149.8 | 465 | 472 |
| 750 | 150 | 452 | 475 |
| 751 | 150.2 | 463 | 478 |
| 752 | 150.4 | 492 | 488 |
| 753 | 150.6 | 465 | 488 |
| 754 | 150.8 | 479 | 495 |
| 755 | 151 | 483 | 490 |
| 756 | 151.2 | 464 | 488 |
| 757 | 151.4 | 473 | 481 |
| 758 | 151.6 | 476 | 495 |
| 759 | 151.8 | 479 | 486 |
| 760 | 152 | 483 | 489 |
| 761 | 152.2 | 465 | 496 |
| 762 | 152.4 | 483 | 489 |
| 763 | 152.6 | 478 | 490 |
| 764 | 152.8 | 467 | 476 |
| 765 | 153 | 472 | 497 |
| 766 | 153.2 | 477 | 471 |
| 767 | 153.4 | 468 | 488 |
| 768 | 153.6 | 487 | 494 |
| 769 | 153.8 | 474 | 471 |
| 770 | 154 | 475 | 487 |
| 771 | 154.2 | 475 | 480 |
| 772 | 154.4 | 471 | 479 |
| 773 | 154.6 | 476 | 473 |
| 774 | 154.8 | 474 | 491 |
| 775 | 155 | 472 | 496 |
| 776 | 155.2 | 488 | 488 |
| 777 | 155.4 | 485 | 480 |
| 778 | 155.6 | 488 | 489 |
| 779 | 155.8 | 465 | 491 |
| 780 | 156 | 491 | 488 |
| 781 | 156.2 | 471 | 485 |
| 782 | 156.4 | 466 | 483 |
| 783 | 156.6 | 477 | 506 |
| 784 | 156.8 | 470 | 475 |
| 785 | 157 | 486 | 507 |
| 786 | 157.2 | 483 | 489 |
| 787 | 157.4 | 467 | 485 |
| 788 | 157.6 | 478 | 470 |
| 789 | 157.8 | 478 | 480 |
| 790 | 158 | 482 | 489 |
| 791 | 158.2 | 488 | 499 |
| 792 | 158.4 | 473 | 473 |
| 793 | 158.6 | 481 | 470 |
| 794 | 158.8 | 482 | 480 |
| 795 | 159 | 487 | 487 |
| 796 | 159.2 | 482 | 496 |
| 797 | 159.4 | 477 | 501 |
| 798 | 159.6 | 494 | 492 |
| 799 | 159.8 | 494 | 497 |
| 800 | 160 | 496 | 490 |
| 801 | 160.2 | 464 | 494 |
| 802 | 160.4 | 475 | 500 |
| 803 | 160.6 | 477 | 492 |
| 804 | 160.8 | 483 | 489 |
| 805 | 161 | 473 | 493 |
| 806 | 161.2 | 473 | 496 |
| 807 | 161.4 | 474 | 510 |
| 808 | 161.6 | 480 | 495 |
| 809 | 161.8 | 474 | 488 |
| 810 | 162 | 479 | 489 |
| 811 | 162.2 | 487 | 504 |
| 812 | 162.4 | 475 | 491 |
| 813 | 162.6 | 473 | 473 |
| 814 | 162.8 | 467 | 479 |
| 815 | 163 | 467 | 481 |
| 816 | 163.2 | 478 | 500 |
| 817 | 163.4 | 477 | 496 |
| 818 | 163.6 | 472 | 497 |
| 819 | 163.8 | 472 | 481 |
| 820 | 164 | 484 | 482 |
| 821 | 164.2 | 492 | 484 |
| 822 | 164.4 | 485 | 490 |
| 823 | 164.6 | 476 | 477 |
| 824 | 164.8 | 483 | 492 |
| 825 | 165 | 483 | 488 |
| 826 | 165.2 | 482 | 496 |
| 827 | 165.4 | 484 | 495 |
| 828 | 165.6 | 486 | 500 |
| 829 | 165.8 | 485 | 492 |
| 830 | 166 | 469 | 519 |
| 831 | 166.2 | 498 | 497 |
| 832 | 166.4 | 488 | 478 |
| 833 | 166.6 | 472 | 503 |
| 834 | 166.8 | 464 | 497 |
| 835 | 167 | 474 | 504 |
| 836 | 167.2 | 478 | 494 |
| 837 | 167.4 | 463 | 484 |
| 838 | 167.6 | 459 | 493 |
| 839 | 167.8 | 491 | 498 |
| 840 | 168 | 474 | 494 |
| 841 | 168.2 | 476 | 495 |
| 842 | 168.4 | 489 | 499 |
| 843 | 168.6 | 482 | 515 |
| 844 | 168.8 | 483 | 497 |
| 845 | 169 | 471 | 485 |
| 846 | 169.2 | 470 | 505 |
| 847 | 169.4 | 479 | 498 |
| 848 | 169.6 | 474 | 501 |
| 849 | 169.8 | 487 | 497 |
| 850 | 170 | 473 | 499 |
| 851 | 170.2 | 461 | 495 |
| 852 | 170.4 | 480 | 498 |
| 853 | 170.6 | 479 | 491 |
| 854 | 170.8 | 465 | 495 |
| 855 | 171 | 480 | 489 |
| 856 | 171.2 | 469 | 482 |
| 857 | 171.4 | 476 | 501 |
| 858 | 171.6 | 453 | 485 |
| 859 | 171.8 | 482 | 485 |
| 860 | 172 | 501 | 491 |
| 861 | 172.2 | 473 | 498 |
| 862 | 172.4 | 484 | 485 |
| 863 | 172.6 | 485 | 486 |
| 864 | 172.8 | 470 | 494 |
| 865 | 173 | 485 | 488 |
| 866 | 173.2 | 480 | 497 |
| 867 | 173.4 | 483 | 485 |
| 868 | 173.6 | 487 | 490 |
| 869 | 173.8 | 477 | 499 |
| 870 | 174 | 473 | 475 |
| 871 | 174.2 | 478 | 483 |
| 872 | 174.4 | 482 | 498 |
| 873 | 174.6 | 484 | 480 |
| 874 | 174.8 | 482 | 476 |
| 875 | 175 | 466 | 488 |
| 876 | 175.2 | 475 | 481 |
| 877 | 175.4 | 462 | 496 |
| 878 | 175.6 | 462 | 483 |
| 879 | 175.8 | 473 | 482 |
| 880 | 176 | 473 | 485 |
| 881 | 176.2 | 464 | 501 |
| 882 | 176.4 | 473 | 480 |
| 883 | 176.6 | 478 | 496 |
| 884 | 176.8 | 459 | 474 |
| 885 | 177 | 471 | 495 |
| 886 | 177.2 | 471 | 483 |
| 887 | 177.4 | 469 | 467 |
| 888 | 177.6 | 483 | 476 |
| 889 | 177.8 | 473 | 493 |
| 890 | 178 | 466 | 494 |
| 891 | 178.2 | 465 | 487 |
| 892 | 178.4 | 464 | 474 |
| 893 | 178.6 | 476 | 472 |
| 894 | 178.8 | 459 | 477 |
| 895 | 179 | 464 | 482 |
| 896 | 179.2 | 465 | 479 |
| 897 | 179.4 | 467 | 468 |
| 898 | 179.6 | 473 | 487 |
| 899 | 179.8 | 480 | 497 |
| 900 | 180 | 474 | 491 |
| 901 | 180.2 | 463 | 471 |
| 902 | 180.4 | 475 | 469 |
| 903 | 180.6 | 473 | 477 |
| 904 | 180.8 | 478 | 474 |
| 905 | 181 | 475 | 474 |
| 906 | 181.2 | 467 | 485 |
| 907 | 181.4 | 481 | 487 |
| 908 | 181.6 | 491 | 470 |
| 909 | 181.8 | 482 | 489 |
| 910 | 182 | 470 | 477 |
| 911 | 182.2 | 483 | 495 |
| 912 | 182.4 | 478 | 467 |
| 913 | 182.6 | 469 | 477 |
| 914 | 182.8 | 483 | 483 |
| 915 | 183 | 473 | 486 |
| 916 | 183.2 | 481 | 477 |
| 917 | 183.4 | 471 | 486 |
| 918 | 183.6 | 476 | 473 |
| 919 | 183.8 | 494 | 485 |
| 920 | 184 | 484 | 468 |
| 921 | 184.2 | 490 | 464 |
| 922 | 184.4 | 483 | 478 |
| 923 | 184.6 | 482 | 461 |
| 924 | 184.8 | 495 | 477 |
| 925 | 185 | 488 | 471 |
| 926 | 185.2 | 482 | 468 |
| 927 | 185.4 | 476 | 460 |
| 928 | 185.6 | 491 | 486 |
| 929 | 185.8 | 485 | 482 |
| 930 | 186 | 451 | 465 |
| 931 | 186.2 | 480 | 453 |
| 932 | 186.4 | 495 | 469 |
| 933 | 186.6 | 486 | 459 |
| 934 | 186.8 | 498 | 467 |
| 935 | 187 | 487 | 454 |
| 936 | 187.2 | 468 | 458 |
| 937 | 187.4 | 483 | 471 |
| 938 | 187.6 | 486 | 490 |
| 939 | 187.8 | 491 | 481 |
| 940 | 188 | 482 | 474 |
| 941 | 188.2 | 488 | 476 |
| 942 | 188.4 | 477 | 469 |
| 943 | 188.6 | 497 | 466 |
| 944 | 188.8 | 487 | 476 |
| 945 | 189 | 472 | 466 |
| 946 | 189.2 | 475 | 487 |
| 947 | 189.4 | 484 | 504 |
| 948 | 189.6 | 471 | 477 |
| 949 | 189.8 | 468 | 497 |
| 950 | 190 | 480 | 497 |
| 951 | 190.2 | 483 | 486 |
| 952 | 190.4 | 476 | 488 |
| 953 | 190.6 | 471 | 481 |
| 954 | 190.8 | 471 | 476 |
| 955 | 191 | 473 | 474 |
| 956 | 191.2 | 461 | 481 |
| 957 | 191.4 | 465 | 492 |
| 958 | 191.6 | 481 | 494 |
| 959 | 191.8 | 478 | 487 |
| 960 | 192 | 480 | 494 |
| 961 | 192.2 | 486 | 493 |
| 962 | 192.4 | 473 | 482 |
| 963 | 192.6 | 468 | 503 |
| 964 | 192.8 | 480 | 492 |
| 965 | 193 | 486 | 488 |
| 966 | 193.2 | 475 | 501 |
| 967 | 193.4 | 475 | 488 |
| 968 | 193.6 | 483 | 476 |
| 969 | 193.8 | 476 | 496 |
| 970 | 194 | 481 | 499 |
| 971 | 194.2 | 468 | 483 |
| 972 | 194.4 | 478 | 492 |
| 973 | 194.6 | 467 | 503 |
| 974 | 194.8 | 461 | 490 |
| 975 | 195 | 477 | 482 |
| 976 | 195.2 | 477 | 485 |
| 977 | 195.4 | 469 | 481 |
| 978 | 195.6 | 469 | 481 |
| 979 | 195.8 | 476 | 484 |
| 980 | 196 | 472 | 471 |
| 981 | 196.2 | 481 | 476 |
| 982 | 196.4 | 485 | 485 |
| 983 | 196.6 | 477 | 492 |
| 984 | 196.8 | 471 | 477 |
| 985 | 197 | 458 | 494 |
| 986 | 197.2 | 480 | 475 |
| 987 | 197.4 | 482 | 496 |
| 988 | 197.6 | 477 | 492 |
| 989 | 197.8 | 485 | 496 |
| 990 | 198 | 494 | 498 |
| 991 | 198.2 | 497 | 500 |
| 992 | 198.4 | 484 | 495 |
| 993 | 198.6 | 490 | 493 |
| 994 | 198.8 | 469 | 466 |
| 995 | 199 | 485 | 483 |
| 996 | 199.2 | 484 | 489 |
| 997 | 199.4 | 496 | 487 |
| 998 | 199.6 | 477 | 495 |
| 999 | 199.8 | 486 | 492 |
| 1000 | 200 | 488 | 479 |
| 1001 | 200.2 | 474 | 486 |
