# Supplementary material for: Immunoinformatics and reverse vaccinology design of self-amplifying RNA vaccine against marburg virus with structural dynamics and immune simulation analysis
Source: PLoS One. 2026 Oct 23;21(10):e0361136. doi: 10.1371/journal.pone.0361136 (PMC13693908; doi:10.1371/journal.pone.0361136)
Supplement: Supplementary file 7 [file pone.0361136.s006.docx]

**Supplementary Table 1:** Sequences of saRNA Vaccine Components.

| **Components** | **Seq** |
| --- | --- |
| CAP | 5’m27,2′−OGppSpG |
| 5’UTR (human β globin) | TTGCAATGAAAATAAATGTTTTTTATTAGGCAGAATCCAGATGCTCAAGGC CCTTCATAATATCCCCCAGTTTAGTAGTTGGACTTAGGGAACAAAGGAACC TTTAATAGAAATTGGACAGCAAGAAAGCGAGCTTAGTGATACTTGTGGGC CAGGGCATTAGCCACACCAGCCACCACTTTCTGATAGGCAGCCTGCACTG GTGGGGTGAATTCTTTGCCAAAGTGATGGGCCAGCACACAGACCAGCACG TTGCCCAGGAGCTGTGGGAGGAAGATAAGAGGTATGAACATGATTAGCAA AAGGGCCTAGCTTGGACTCAGAATAATCCAGCCTTATCCCAACCATAAAA TAAAAGCAGAATGGTAGCTGGATTGTAGCTGCTATTAGCAATATGAAACC TCTTACATCAGTTACAATTTATATGCAGAAATATTTATATGCAGAGATATT GCTATTGCCTTAACCCAGAAATTATCACTGTTATTCTTTAGAATGGTGCAA AGAGGCATGATACATTGTATCATTATTGCCCTGAAAGAAAGAGATTAGGG AAAGTATTAGAAATAAGATAAACAAAAAAGTATATTAAAAGAAGAAAGC ATTTTTTAAAATTACAAATGCAAAATTACCCTGATTTGGTCAATATGTGTA CACATATTAAAACATTACACTTTAACCCATAAATATGTATAATGATTATGT ATCAATTAAAAATAAAAGAAAATAAAGTAGGGAGATTATGAATATGCAAATAAGCACACATATATTCCAAATAGTAATGTACTAGGCAGACTGTGTAAAGTTTTTTTTTAAGTTACTTAATGTATCTCAGAGATATTTCCTTTTGTTATACACAATGTTAAGGCATTAAGTATAATAGTAAAAATTGCGGAGAAGAAAAAAAAAGAAAGCAAGAATTAAACAAAAGAAAACAATTGTTATGAACAGCAAATAAAAGAAACTAAAACGATCCTGAGACTTCCACACTGATGCAATCATTCGTCTGTTTCCCATTCTAAACTGTACCCTGTTACTTATCCCCTTCCTATGACATGAACTTAACCATAGAAAAGAAGGGGAAAGAAAACATCAAGCGTCCCATAGACTCACCCTGAAGTTCTCAGGATCCACGTGCAGCTTGTCACAGTGCAGCTCACTCAGTGTGGCAAAGGTGCCCTTGAGGTTGTCCAGGTGAGCCAGGCCATCACTAAAGGCACCGAGCACTTTCTTGCCATGAGCCTTCACCTTAGGGTTGCCCATAACAGCATCAGGAGTGGACAGATCCCCAAAGGACTCAAAGAACCTCTGGGTCCAAGGGTAGACCACCAGCAGCCTAAGGGTGGGAAAATAGACCAATAGGCAGAGAGAGTCAGTGCCTATCAGAAACCCAAGAGTCTTCTCTGTCTCCACATGCCCAGTTTCTATTGGTCTCCTTAAACCTGTCTTGTAACCTTGATACCAACCTGCCCAGGGCCTCACCACCAACTTCATCCACGTTCACCTTGCCCCACAGGGCAGTAACGGCAGACTTCTCCTCAGGAGTCAGATGCACCATGGTGTCTGTTTGAGGTTGCTAGTGAACACAGTTGTGTCAGAAGCAAATGT |
| ORF1 (NS1-4 genes) | ATGGGCGGCGCAAGAGAGAAGCCCAAACCAATTACCTACCCAAAATGGA GAAAGTTCACGTTGACATCGAGGAAGACAGCCCATTCCTCAGAGCTTTAC AACGGAGCTTCCCGCAGTTTGAGGTAGAAGCCAAGCAGGTCACTGATAAT GACCATGCTAATGCCAGAGCGTTTTCGCATCTGGCTTCAAAACTGATCGAA ACGGAGGTGGACCCATCCGACACGATCCTTGACATTGGAAGTGCGCCCGC CCGCAGAATGTATTCTAAGCATAAGTATCATTGCATCTGTCCGATGAGATG TGCGGAAGATCCGGACAGATTGTACAAGTATGCAACTAAGCTGAAGAAAA ATTGCAAGGAAATAACTGACAAGGAATTGGACAAGAAAATGAAGGAGCT CGCCGCCGTCATGAGCGACCCTGACCTGGAAACTGAGACTATGTGCCTCC ACGACGATGAGTCATGTCGCTACGAGGGGCAAGTCGCTGTTTACCAGGAT GTATACGCAGTTGACGGACCGACAAGTCTCTATCACCAAGCCAACAAGGG AGTTAGAGTCGCCTACTGGATAGGCTTTGACACCACCCCTTTTATGTTTAA GAACTTGGCTGGAGCATATCCATCATACTCTACCAACTGGGCCGACGAAA CCGTGTTAACGGCTCGTAACATAGGCCTATGCAGCTCCGACGTCATGGAGCGGTCACGTAGAGGGATGTCCATTCTTAGGAAGAAGTATTTGAAACCATCCAATAATGTCCTATTCTCTGTTGGCTCGACCATCTACCACGAGAAGAGGGACTTACTGAGGAGCTGGCACCTGCCGTCTGTATTTCACTTACGTGGCAAGCAAAATTACACATGTCGGTGTGAGACTATAGTTAGTTGCGACGGGTACGTCGTTAAAAGAATAGCTATCAGTCCAGGCCTGTATGGGAAGCCTTCAGGCTATGCTGCTACGATGCACCGCGAGGGATTCTTGTGCTGCAAAGTGACAGACACATTGAACGGGGAGAGGGTCTCTTTTCCCGTGTGCACGTATGTGCCAGCTACAT TGTGTGACCAAATGACTGGCATACTGGCAACAGATGTCAGTGCGGACGAC GCGCAAAAACTGCTGGTTGGGCTCAACCAGCGCATAGTCGTCAACGGTCG CACCCAAAGAAACACCAATACCATGAAGAATTATCTTTTGCCCGTAGTGG CCCAGGCATTTGCTAGGTGGGCAAAGGAATATAAGGAAGATCAAGAAGATGAGAGGCCACTAGGACTACGAGATAGACAGTTAGTCATGGGGTGCTGCTG GGCTTTTAGAAGGCACAAGATAACATCTATTTATAAGCGCCCAGATACCC AAACCATCATCAAAGTGAACAGCGATTTCCACTCATTCGTGCTGCCCAGGA TAGGCAGTAACACACTGGAGATCGGGCTGAGAACGAGAATCAGGAAAAT GCTAGAAGAGCACAAGGAGCCGTCACCTCTCATTACTGCCGAGGACATAC AAGAGGCTAAGTGCGCAGCCGATGAGGCTAAGGAAGTGCGTGAAGCCGA GGAGCTGCGCGCTGCTCTACCACCTTTGGCAGCTGATTTTGAGGAGCCCAC TCTGGAAGCCGATGTCGACTTGATGTTACAAGAGGCTGGGGCCGGCTCAG TGGAGACACCTCGTGGCTTGATAAAGGTTACCAGCTATGCCGGCGAGGAC AAGATCGGCTCTTACGCAGTGCTTTCTCCACAGGCTGTACTCAAGAGTGAG AAACTATCTTGCATTCACCCTCTCGCTGAACAAGTCATAGTGATAACACAC TCTGGCCGAAAAGGGCGTTATGCCGTGGAACCCTACCATGGAAAAGTAGT GGTGCCAGAGGGACATGCAATACCCGTCCAGGACTTTCAAGCTCTGAGTG AAAGTGCCACCATCGTGTACAACGAACGAGAGTTCGTAAACAGGTACCTG CACCATATTGCCACACATGGAGGAGCGCTGAACACAGATGAAGAATATTA CAAAACTGTCAAGCCCAGCGAGCACGACGGCGAATACCTGTACGACATCG ACAGGAAACAATGCGTCAAGAAAGAATTAGTCACTGGGCTAGGGCTTACA GGCGAGCTGGTGGATCCTCCCTTCCATGAATTTGCCTACGAGAGTCTGAGA ACACGTCCGGCCGCTCCTTACCAAGTACCAACCATAGGGGTGTATGGCGT GCCGGGGTCAGGCAAGTCTGGCATCATTAAAAGCGCAGTCACCAAAAAAG ATCTGGTGGTGAGCGCCAAGAAAGAAAACTGCGCAGAAATAATAAGGGA CGTCAAGAAAATGAAAGGGCTGGACGTCAATGCCAGAACTGTGGACTCAG TGCTCTTGAATGGATGCAAACACCCCGTAGAGACCCTGTATATTGACGAA GCTTTTGCTTGTCATGCAGGCACTCTCAGAGCGCTCATAGCCATCATAAGA CCTAAAAAGGCAGTGCTCTGCGGGGATCCAAAACAGTGTGGCTTTTTCAAT ATGATGTGCCTGAAAGTGCATTTTAACCACGAGATTTGCACGCAGGTCTTCC  ACAAAAGCATCTCTCGCCGTTGCACTAAATCCGTGACTTCGGTCGTCTCA ACCTTGTTTTACGACAAAAGGATGAGAACGACGAACCCGAAAGAGACTAA GATTGTGATTGACACTACTGGCAGTACCAAACCGAAGCAGGACGATCTCA TTCTCACTTGTTTCAGAGGGTGGGTGAAGCAGTTGCAAATAGATTACAAAG GCAACGAAATAATGACGGCAGCTGCCTCTCAAGGGCTGACCCGTAAAGGC GTGTATGCCGTTCGGTACAAGGTGAATGAAAATCCCCTGTACGCACCCACC TCAGAACATGTGAACGTCCTACTGACCCGCACGGAGGACCGTATCGTGTG GAAAACACTAGCCGGTGATCCATGGATAAAAATACTGACGGCCAAGTATC CTGGGAACTTCACTGCCACGATAGAGGAATGGCAAGCAGAGCATGATGCC ATCATGAGGCACATCTTGGAGAGACCGGACCCTACCGACGTTTTCCAAAA TAAGGCGAACGTGTGTTGGGCCAAGGCTTTGGTGCCGGTACTGAAGACTG CAGGCATAGACATGACCACTGAACAATGGAACACTGTGGATTACTTCGAA ACGGACAAAGCTCACTCAGCAGAGATAGTATTGAACCAACTATGCGTGAG GTTCTTTGGACTCGACCTGGACTCCGGTCTATTTTCTGCACCCACTGTTCCG TTATCCATTAGGAATAATCACTGGGATAATTCCCCGTCGCCTAACATGTAC GGGTTGAATAAAGAAGTGGTCCGCCAGCTCTCCCGCAGGTACCCACAACT GCCTCGAGCAGTTGCCACCGGAAGAGTCTATGACATGAACACTGGCACGC TGCGCAATTATGATCCGCGCATAAATCTAGTACCTGTGAACAGAAGACTG CCTCATGCTTTAGTCCTCCACCATAATGAACACCCACAGAGTGACTTTTCT TCATTCGTCAGCAAACTGAAGGGCAGAACTGTCTTGGTGGTCGGGGAGAA GTTGTCCGTCCCAGGCAAAAAGGTCGACTGGTTGTCAGACCAGCCTGAGG CTACCTTTAGAGCTCGGCTGGATTTAGGTATCCCAGGTGACGTGCCCAAAT ACGACATTGTATTTATTAACGTGAGGACTCCATATAAATACCATCATTATC AGCAGTGTGAAGACCACGCCATTAAGCTTAGTATGTTGACCAAGAAAGCT TGTCTGCATTTGAATCCCGGCGGAACCTGCGTCAGCATAGGTTATGGTTAC GCTGACAGGGCCAGCGAGAGCATCATTGGTGCTATAGCGCGGCAGTTCAA GTTCTCCCGGGTATGCAAACCGAAATCCTCACATGAAGAGACAGAAGTAC TGTTTGTATTCATTGGGTACGATCGCAAGGCCCGTACGCACAATCCTTACA AGCTTTCATCTACCTTGACCAACATCTATACAGGTTCCAGACTCCACGAAG CCGGATGCGCACCCTCATATCATGTGGTGCGAGGGGATATTGCCACGGCC ACCGAAGGAGTGATCATAAATGCTGCTAACAGCAAAGGACAACCTGGCGG AGGGGTGTGCGGAGCGCTGTATAAGAAATTCCCGGAAAGCTTCGATTTAC AGCCGATCGAAGTAGGAAAAGCGCGACTGGTCAAAGGTGCAGCTAAACAT ATCATTCATGCCGTAGGACCAAACTTCAACAAAGTTTCGGAAGTTGAAGG GGACAAACAGTTGGCAGAGGCTTATGAGTCCATCGCTAAAATTGTCAACG ATAACAATTACAAGTCAGTAGCGATTCCACTGTTGTCCACCGGCATCTTTT CCGGGAACAAAGATCGACTAACCCAATCATTGAACCATTTGCTGACAGCT TTAGACACCACTGATGCAGATGTAGCCATATACTGCAGGGACAAGAAATG GGAAATGACTCTCAAGGAAGCAGTGGCTAGGAGAGAAGCAGTGGAGGAG ATATGCATATCAGACGACTCTTCGGTGACAGAACCGGATGCAGAGCTGGT GAGGGTACATCCGAAGAGTTCTTTGGCTGGAAGGAAGGGCTACAGCACAA GTGATGGCAAGACTTTCTCATATTTGGAAGGGACCAAATTTCACCAGGCG GCCAAGGATATAGCAGAAATTAATGCCATGTGGCCAGTTGCAACGGAGGC CAATGAGCAAGTATGCATGTATATCCTCGGTGAAAGCATGAGCAGCATTA GGTCGAAATGCCCCGTCGAGGAGTCGGAAGCCTCCACACCACCTAGCACG CTGCCTTGCTTGTGCATCCATGCTATGACTCCAGAAAGAGTACAACGCCTA AAAGCCTCACGTCCAGAACAAATTACTGTGTGCTCATCCTTTCCATTGCCG AAGTATAGAATCACTGGTGTGCAGAAGATCCAGTGCTCCCAGCCTATACT GTTCTCACCGAAGGTGCCTGCGTACATTCATCCACGGAAGTACCTCGTGGA AACACCACCGGTAGAAGAGACTCCGGAGTCGCCGGCAGAGAACCAATCCA CAGAGGGGACACCTGAACAACCAGCACTTGTAAACGTGGATGCAACCAGG  ACTAGAATGCCTGAACCGATCATCATTGAAGAGGAAGAAGAGGATAGTAT AAGTTTGCTGTCAGACGGCCCGACCCACCAGGTGCTGCAAGTCGAGGCAG ACATTCACGGGTCGCCTTCTGTATCCAGCTCATCCTGGTCCATTCCTCATGC ATCCGACTTTGATGTGGACAGCTTATCCATCCTTGACACCCTGGATGGAGC TAGCGTGACCAGCGGGGCAGTGTCAGCCGAGACTAACTCCTACTTCGCAA GGAGCATGGAGTTTCGGGCGCGACCGGTGCCTGCGCCTCGAACCGTATTC AGGAACCCTCCACATCCCGCACCGCGCACAAGAACACCGCCACTTGCACA CAGCAGGGCCAGCTCGAGAACTAGCCTAGTTTCCACCCCGCCAGGCGTGA ATAGGGTGATTACTAGAGAGGAGCTCGAGGCGCTTACCCCGTCCCGCGCT CCTAGCAGGTCGGCCTCAAGAACTAGCCTGGTCTCTAACCCGCCAGGCGT AAATAGGGTGATTACAAGAGAGGAGTTTGAGGCGTTCGTAGCACAACAAC AATGACGGTTTGACGCGGGTGCATACATCTTTTCCTCCGATACCGGTCAAG GGCATTTACAACAAAAATCAGTAAGGCAAACGGTGTTATCCGAAGTGGTG TTGGAGAGGACCGAATTGGAGATTTCGTATGCCCCGCGCCTCGACCAGGA AAAAGAAGAACTACTACGCAAGAAATTACAGCTGAATCCCACACCTGCTA ACAGAAGCAGATACCAGTCCAGGAGGGTGGAGAATATGAAAGCCATAAC AGCTAGACGTATTCTGCAAGGCCTAGGGCATTATTTGAAGGCAGAAGGAA AAGTGGAGTGCTATCGAACCCTGCATCCTGTTCCTTTGTATTCATCTAGTGT GAATCGTGCTTTTTCAAGCCCCAAGGTCGCAGTGGAAGCCTGCAATGCCAT GCTGAAAGAAAATTTTCCGACTGTAGCTTCCTACTGTATTATTCCAGAGTA CGATGCCTATCTGGACATGGTTGACGGCGCTTCTTGTTGCTTAGACACTGC CAGTTTTTGCCCTGCGAAGCTGCGCAGCTTTCCAAAGAAACACTCCTATTT GGAACCCACAATACGGTCGGCAGTGCCATCAGCGATTCAGAACACGCTCC AGAACGTCCTGGCAGCTGCCACAAAAAGAAATTGCAACGTCACGCAAATG AGAGAATTGCCCGTATTGGATTCGGCTGCCTTTAATGTGGAATGCTTCAAG AAATATGCGTGCAATAATGAATATTGGGAAACGTTTAAAGAAAACCCCAT CAGGCTTACTGAAGAAAATGTGGTAAATTACATTACTAAATTAAAAGGAC CAAAAGCTGCTGCTCTTTTTGCGAAGACACATAATTTGAATATGTTACAGG ACATACCAATGGACAGGTTTGTAATGGACTTAAAGAGGGACGTGAAAGTG ACTCCAGGAACAAAACATACTGAAGAACGGCCCAAGGTACAGGTGATTCA GGCTGCCGATCCACTAGCGACAGCGGATCTGTGCGGAATCCACCGGGAGT TGGTTAGGAGATTAAATGCTGTCCTGCTTCCGAACATCCATACACTGTTTG ACATGTCGGCTGAAGACTTTGACGCTATTATTGCCGAGCATTTCCAGCCTG GGGACTGTGTACTGGAAACTGACATTGCGTCGTTTGATAAAAGTGAGGAC GACGCCATGGCTCTGACCGCGTTAATGATTCTGGAAGACCTAGGAGTGGA CGCAGAGCTGTTGACGCTGATTGAGGCGGCTTTCGGCGAAATATCATCAAT ACATTTGCCCACCAAAACTAAATTTAAATTCGGAGCCATGATGAAATCCG GAATGTTCCTCACACTGTTTGTGAACACAGTCATCAACATCGTAATCGCAA GCAGAGTGTTAAGAGAGCGGCTAACCGGATCACCATGTGCAGCATTCATT GGAGATGACAATATCGTGAAAGGAGTCAAATCTGACAAATTAATGGCAGA CAGGTGCGCCACTTGGTTGAACATGGAAGTCAAGATCATAGACGCCGTGG TGGGCGAGAAAGCGCCCTATTTTTGTGGAGGGTTTATCTTGTGTGACTCCG TGACCGGCACAGCGTGCCGTGTGGCAGACCCCCTAAAAAGGCTGTTTAAG CTTGGCAAACCCCTGGCAGTAGACGATGAACATGACGATGACAGGAGAAG GGCATTACACGAAGAGTCAACACGCTGGAATCGAGTGGGAATTCTTCCAG AGCTGTGTAAGGCAGTAGAATCAAGGTATGAAACCGTAGGAACTTCCATC ATAGTTATGGCCATGACTACTCTAGCTAGCAGTGTTAAATCATTCAGCTAC CTGAGAGGGGCCCCTATAACTCTCTACGGCTAA |
| Kozak sequence | GCCGCCACCATGG |
| tPA (Signal peptide) | MDAMKRGLCCVLLLCGAVFVSPSQEIHARFRRGARSYQVICRDEKTQMIYQQHQSWLRPVLRSNRVEYCWCNSGRAQCHSVPVKSCSEPRCFNGGTCQQALYFSDFVCQCPEGFAGKCCEIDTRATCYEDQGISYRGTWSTAESGAECTNWNSSALAQKPYSGRRPDAIRLGLGNHNYCRNPDRDSKPWCYVFKAGKYSSEFCSTPACSEGNSDCYFGNGSAYRGTHSLTESGASCLPWNSMILIGKVYTAQNPSAQALGLGKHNYCRNPDGDAKPWCHVLKNRRLTWEYCDVPSCSTCGLRQYSQPQFRIKGGLFADIASHPWQAAIFAKHRRSPGERFLCGGILISSCWILSAAHCFQERFPPHHLTVILGRTYRVVPGEEEQKFEVEKYIVHKEFDDDTYDNDIALLQLKSDSSRCAQESSVVRTVCLPPADLQLPDWTECELSGYGKHEALSPFYSERLKEAHVRLYPSSRCTSQHLLNRTVTDNMLCAGDTRSGGPQANLHDACQGDSGGPLVCLNDGRMTLVGIISWGLGCGQKDVPGVYTKVTNYLDWIRDNMRP |
| ORF2 (MB-saVaxI) | MAKLSTDELLDAFKEMTLLELSDFVKKFEETFEVTAAAPVAVAAAGAAPAGAAVEAAEEQSEFDVILEAAGDKKIGVIKVVREIVSGLGLKEAKDLVDGAPKPLLEKVAKEAADEAKAKLEAAGATVTVK**EAAAK**FGPGIEGLY**AAY**LLLLSIAVL**AAY**AIALGVATA**AAY**QLSAIALGV**AAY**VAALLTGSY**AAY**AQVLSKIAY**AAY**TEERTFSLINRHAID**GPGPG**EERTFSLINRHAIDF**GPGPG**HEHQEIQAIAEDDEE**GPGPG**LNLSGINNLEHGLYP**GPGPG**ITPNYVGDLNLDDQF**GPGPG**EGENAQAALTRLSRT**GPGPG**IDFDPVPNTKTIFDESSSSGASAEEDQHASPN**KK**QKVADSPLEAS**KK**TGVPPKNVEYTEGEEAK**KK**YQNPVSAKELQADWPDMSFD**KK**MASSSNYN**KK**YKSGKSG**HHHHHH** |
| ORF2 (MB-saVaxII) | MAKLSTDELLDAFKEMTLLELSDFVKKFEETFEVTAAAPVAVAAAGAAPAGAAVEAAEEQSEFDVILEAAGDKKIGVIKVVREIVSGLGLKEAKDLVDGAPKPLLEKVAKEAADEAKAKLEAAGATVTVK**EAAAK**DDLAAGLSW**AAY**HAQGIALHL**AAY**APFARVLNL**AAY**GLYPQLSAI**AAY**AYKSGKSGA**AAY**KSGKSGAFL**AAY**AYANPTLSA**AAY**DMFSVKEGM**AAY**SGQKVADSPLEASKR**GPGPG**AKTCYNISVTDPSGK**GPGPG**EQGIVTYPNHWLTTG**GPGPG**HGEYAPFARVLNLSG**GPGPG**KIAYKSGKSGAFLDA**GPGPG**SKIAYKSGKSGAFLD**GPGPG**GITPNYVGDLNLDDQ**GPGPG**LNGFNNRQVVLAYAN**GPGPG**IDFDPVPNTKTIFDESSSSGASAEEDQHASPN**KK**TKPTAPHVR**KK**VGEQYQQL**KK**LRAVPPNPTID**KK**PENFPLN**HHHHHH** |
| MITD | MAKSGLRQDPQSTAAATVLKRAVELDSESRYPQALVCYQEGIDLLLQVLKGTKDNTKRCNLREKISKYMDRAENIKKYLDQEKEDGKYHKQIKIEENATGFSYESLFREYLNETVTEVWIEDPYIRHTHQLYNFLRFCEMLIKRPCKVKTIHLLTSLDEGIEQVQQSRGLQEIEESLRSHGVLLEVQYSSSIHDREIRFNNGWMIKIGRGLDYFKKPQSRFSLGYCDFDLRPCHETTVDIFHKKHTKNI |
| Stop Codon | TGA |
| 3' UTR | ACTCTTCTGGTCCCCACAGACTCAGAGAGAACCCACCATGGTGCTGTCTCCTGCCGACAAGACCAACGTCAAGGCCGCCTGGGGTAAGGTCGGCGCGCACGCTGGCGAGTATGGTGCGGAGGCCCTGGAGAGGTGAGGCTCCCTCCCCTGCTCCGACCCGGGCTCCTCGCCCGCCCGGACCCACAGGCCACCCTCAACCGTCCTGGCCCCGGACCCAAACCCCACCCCTCACTCTGCTTCTCCCCGCAGGATGTTCCTGTCCTTCCCCACCACCAAGACCTACTTCCCGCACTTCGACCTGAGCCACGGCTCTGCCCAGGTTAAGGGCCACGGCAAGAAGGTGGCCGACGCGCTGACCAACGCCGTGGCGCACGTGGACGACATGCCCAACGCGCTGTCCGCCCTGAGCGACCTGCACGCGCACAAGCTTCGGGTGGACCCGGTCAACTTCAAGGTGAGCGGCGGGCCGGGAGCGATCTGGGTCGAGGGGCGAGATGGCGCCTTCCTCGCAGGGCAGAGGATCACGCGGGTTGCGGGAGGTGTAGCGCAGGCGGCGGCTGCGGGCCTGGGCCCTCGGCCCCACTGACCCTCTTCTCTGCACAGCTCCTAAGCCACTGCCTGCTGGTGACCCTGGCCGCCCACCTCCCCGCCGAGTTCACCCCTGCGGTGCACGCCTCCCTGGACAAGTTCCTGGCTTCTGTGAGCACCGTGCTGACCTCCAAATACCGTTAAGCTGGAGCCTCGGTGGCCATGCTTCTTGCCCCTTGGGCCTCCCCCCAGCCCCTCCTCCCCTTCCTGCACCCGTACCCC CGTGGTCTTTGAATAAAGTCTGAGTGGGCGGCA |
| Poly A tail | AAAAAAAAAAAAAAAAAAAAAAAAAAAAAAAAAAAAAAAAAAAAAAAAAAAAAAAAAAAAAAAAAAAAAAAAAAAAAAAAAAAAAAAAAAAAAAAA AAAAAAAAAAAAAAAAAAAAAAAA |
