# Supplementary material for: Immunoinformatics and reverse vaccinology design of self-amplifying RNA vaccine against marburg virus with structural dynamics and immune simulation analysis
Source: PLoS One. 2026 Oct 23;21(10):e0361136. doi: 10.1371/journal.pone.0361136 (PMC13693908; doi:10.1371/journal.pone.0361136)
Supplement: Supplementary file 8 [file pone.0361136.s009.docx]

**Response to Reviewers**

Thank you to the reviewers for their insightful comments and suggestions, which helped improve the manuscript.

**Reviewer #1**

1. In the Abstract, the statement that “Furthermore, MB-saVaxI and MB- saVaxII were effectively cloned into Escherichia coli,……” should be specified if this cloning was in vitro or in silico (predicted).

**Response:** The nature of the cloning procedure has been clarified in the Abstract of the revised manuscript as “Furthermore, MB-saVaxI and MB-saVaxII were effectively cloned *in silico* into *Escherichia coli,……….”*

2. On page 9 last line, the authors should please define CFR on first use

**Response:** The full form of CFR was mentioned in the introduction as “The largest and most lethal outbreak occurred in Angola during 2004-2005, involving 252 cases and resulting in 227 deaths, corresponding to a case fatality rate (CFR) of ~90%, thereby underscoring the extreme lethality of the virus.”

3. Page 10, 3rd to 6th line, authors report 100% fatality rate, and later contradict with a 24% to 88% mortality rate range for the disease. Please resolve.

**Response:** The apparent discrepancy between the 100% fatality rate reported for the 2025 Tanzania outbreak and the general 24–88% CFR range has been clarified in the Introduction of the revised manuscript as “The most recent outbreak occurred in the United Republic of Tanzania in March 2025, with 10 cases and 10 deaths, corresponding to a 100% case fatality rate (16). The recurring nature of MARV outbreaks underscores the urgent need for improved zoonotic surveillance, rapid diagnostic tools, and coordinated global response mechanisms to mitigate the impact of the deadly disease (8). Due to its high mortality rate, ranging from 24% to 88% depending on outbreak conditions and case management, the World Health Organization (WHO) designated MARV as a priority pathogen in 2018 (10,17).”

4. The background is well written, however, not much was discussed about the leading vaccine candidates currently in clinical trials, including the Sabin’s Marburg vaccine candidate phase II trial underway in Uganda and Kenya, and the United States. The authors might want to review the leading Marburg vaccine candidates and highlight gaps and limitations which the candidates (MB-saVaxI and MB-saVaxII) described herein are intended to overcome.

**Response:** The leading Marburg vaccine candidates and their limitations have been discussed in the Discussion section of the revised manuscript as follows: “The most clinically advanced candidate, the Sabin Vaccine Institute's chimpanzee adenovirus type 3-vectored vaccine (cAd3-MARV) encoding the MARV glycoprotein, was safe and immunogenic in a first-in-human phase 1 trial in the United States (NCT03475056), with 95% of participants developing glycoprotein-specific antibodies (1). Nevertheless, as a replication-incompetent viral vector, cAd3-MARV may be difficult to administer repeatedly because pre-existing anti-vector immunity can reduce its effectiveness, particularly in adenovirus-exposed populations. In addition, its clinical efficacy has not yet been demonstrated. Similarly, live-attenuated rVSV∆G-MARV-GP candidates (PHV01, NCT06265012; IAVI's rVSV∆G-MARV-GP, NCT07425821) have entered phase 1 testing (2). However, these replication-competent live viral vaccines may cause reactogenicity and viremia and require strict cold-chain storage. In addition, human correlates of protection remain undefined, and their clinical efficacy has not yet been established (3,4).”

5. Method’s sections “2.3. Linear B cell (LBC) epitopes identification” and “2.8. Biophysical features evaluation” both describe prediction of Toxicity, allergenicity, and antigenicity. Please clarify if these were evaluated twice, otherwise, kindly keep the description in the relevant section

**Response:** The distinction between epitope-level and vaccine-level evaluation has been clarified in the revised manuscript. Toxicity, allergenicity, and antigenicity were assessed for the predicted linear B cell epitopes in Section 2.3, whereas the same properties were subsequently evaluated for the final vaccine constructs in Section 2.8. Thus, these analyses were performed at two distinct stages rather than as duplicate evaluations.

6. In Results section 3.4, the sentence “The percentage of epitopes….” Describes what percentage? Identity? Please specify.

**Response:** The type of percentage has been clarified in the Results section (Section 3.4) of the revised manuscript as “The conservancy percentage of epitopes ranges from 94.74% to 89.847% for GP, 100% to 50% for NP, 100% to 88.89% for VP40, and 100% to 75% for VP35 for MB-saVaxI.”

7. Additionally, the supplementary data-1 tables 1 & 2 have wrongly reported percent identity ranges – e.g in supplementary table 1 for GP, I see percentages as low as 21.05% and as high as 100%, but this is different in the text where the range “94.74% to 89.847% ” reported within the text. Please the authors should kindly check across the entire manuscript to ensure data is correctly reported, matching between text and tables/figures; and appropriately cited.

**Response:** The percentage identity ranges in Supplementary Data 1, Tables 1 and 2 have been carefully checked and corrected in the revised manuscript. The reported values have been cross-checked throughout the manuscript to ensure consistency between the text, tables, and figures, with appropriate citations provided.

8. Additionally, the percent identities should be reported in-text in a range that has the smaller percentage coming first, and the larger percentage coming second as the upper limit i.e please reverse the current ranges.

**Response:** All percentage identity ranges reported in the revised manuscript have been carefully checked and revised to present the smaller percentage first and the larger percentage as the upper limit.

9. A separate expanded paragraph detailing the study limitations should be added at the end of the discussion section. This is currently only very narrowly described. Authors should have to include statements explaining how limitations were overcome or not in the current study.

**Response:** The study limitations have been addressed in an expanded paragraph at the end of the Discussion section as, “In light of this, the proposed vaccine structure will probably maintain stability throughout the processes of entry into the host, transcription, and expression. Although the findings were favorable, this study focused exclusively on the immunoinformatics process. This process has limitations, including inaccurate structural predictions and a lack of chronic immunity predictions. Furthermore, all findings were generated through computational predictions, and no experimental validation was performed. Therefore, laboratory and animal studies are needed to confirm the immunogenicity and safety of MB-saVaxI and MB-saVaxII. Likewise, epitope prediction depends on the algorithms and databases used, which may not fully represent all HLA types in the global population. To address this bias, only epitopes with high conservancy were retained. Moreover, molecular dynamics simulations were performed over a limited timescale and may not capture long-term structural behavior. Overall, the stability and expression of the designed saRNA constructs were assessed only *in-silico*. These limitations were partly addressed through the use of validated tools and strict selection criteria. However, experimental studies are required before clinical translation. Further research, including preclinical and clinical studies, will be essential to precisely verify the immunogenicity, efficacy, and stability of these vaccines.”

**Reviewer #2**

1. This is a good and relevant study addressing an important topic in Marburg virus vaccine development. The authors computationally designed two saRNA vaccine candidates, MB-saVaxI and MB-saVaxII, which showed favorable predicted structural, binding, stability, and immune-response profiles. However, several substantial issues require clarification and improvement. Therefore, I recommend Major Revision before the manuscript can be considered for publication.

**Response:** The reviewer’s positive evaluation of the study and constructive comments are sincerely appreciated. All concerns raised have been carefully addressed, and the manuscript has been substantially revised, with the corresponding changes incorporated throughout the revised version.
