# Supplementary material for: Immunoinformatics and reverse vaccinology design of self-amplifying RNA vaccine against marburg virus with structural dynamics and immune simulation analysis
Source: PLoS One. 2026 Oct 23;21(10):e0361136. doi: 10.1371/journal.pone.0361136 (PMC13693908; doi:10.1371/journal.pone.0361136)
Supplement: Peer review history

# Peer review: Decision Letter 0

**DOI:** 10.1371/journal.pone.0361136.r001

**Authors:** Mohammad Faezi Ghasemi

2 Sep 2026

Dear Dr. Mahdeen,

Thank you for submitting your manuscript to PLOS One. After careful consideration, we feel that it has merit but does not fully meet PLOS One’s publication criteria as it currently stands. Therefore, we invite you to submit a revised version of the manuscript that addresses the points raised during the review process.

==============================

**Dear authors,** **Based on the reports that I have received from our reviewers, your manuscript needs a major revision. Please follow all comments provided by reviewers and submit your revised version.** **Best regards,**

==============================

Please submit your revised manuscript by Oct 17 2026 11:59PM. If you will need more time than this to complete your revisions, please reply to this message or contact the journal office at plosone@plos.org. When you're ready to submit your revision, log on to https://www.editorialmanager.com/pone/ and select the 'Submissions Needing Revision' folder to locate your manuscript file.

- A letter that responds to each point raised by the academic editor and reviewer(s). You should upload this letter as a separate file labeled 'Response to Reviewers'.
- A marked-up copy of your manuscript that highlights changes made to the original version. You should upload this as a separate file labeled 'Revised Manuscript with Track Changes'.
- An unmarked version of your revised paper without tracked changes. You should upload this as a separate file labeled 'Manuscript'.

If you would like to make changes to your financial disclosure, please include your updated statement in your cover letter. Guidelines for resubmitting your figure files are available below the reviewer comments at the end of this letter.

If applicable, we recommend that you deposit your laboratory protocols in protocols.io to enhance the reproducibility of your results. Protocols.io assigns your protocol its own identifier (DOI) so that it can be cited independently in the future. For instructions see: https://journals.plos.org/plosone/s/submission-guidelines#loc-laboratory-protocols. Additionally, PLOS One offers an option for publishing peer-reviewed Lab Protocol articles, which describe protocols hosted on protocols.io. Read more information on sharing protocols at https://plos.org/protocols?utm_medium=editorial-email&utm_source=authorletters&utm_campaign=protocols.

As the corresponding author, your ORCID iD is verified in the submission system and will appear in the published article. PLOS supports the use of ORCID, and we encourage all coauthors to register for an ORCID iD and use it as well. Please encourage your coauthors to verify their ORCID iD within the submission system before final acceptance, as unverified ORCID iDs will not appear in the published article. *Only*  the individual author can complete the verification step; PLOS staff *cannot*  verify ORCID iDs on behalf of authors.

We look forward to receiving your revised manuscript.

Kind regards,

Mohammad Faezi Ghasemi, Ph.D

Academic Editor

PLOS One

Journal Requirements:

When submitting your revision, we need you to address these additional requirements.

1. Please ensure that your manuscript meets PLOS ONE's style requirements, including those for file naming. The PLOS ONE style templates can be found at

https://journals.plos.org/plosone/s/file?id=wjVg/PLOSOne_formatting_sample_main_body.pdf and

https://journals.plos.org/plosone/s/file?id=ba62/PLOSOne_formatting_sample_title_authors_affiliations.pdf

2. Please note that PLOS One has specific guidelines on code sharing for submissions in which author-generated code underpins the findings in the manuscript. In these cases, we expect all author-generated code to be made available without restrictions upon publication of the work. Please review our guidelines at https://journals.plos.org/plosone/s/materials-and-software-sharing#loc-sharing-code and ensure that your code is shared in a way that follows best practice and facilitates reproducibility and reuse.

3. When completing the data availability statement of the submission form, you indicated that you will make your data available on acceptance. We strongly recommend all authors decide on a data sharing plan before acceptance, as the process can be lengthy and hold up publication timelines. Please note that, though access restrictions are acceptable now, your entire data will need to be made freely accessible if your manuscript is accepted for publication. This policy applies to all data except where public deposition would breach compliance with the protocol approved by your research ethics board. If you are unable to adhere to our open data policy, please kindly revise your statement to explain your reasoning and we will seek the editor's input on an exemption. Please be assured that, once you have provided your new statement, the assessment of your exemption will not hold up the peer review process.

4. If the reviewer comments include a recommendation to cite specific previously published works, please review and evaluate these publications to determine whether they are relevant and should be cited. There is no requirement to cite these works unless the editor has indicated otherwise.

[Note: HTML markup is below. Please do not edit.]

Reviewers' comments:

Reviewer's Responses to Questions

**Comments to the Author**

1. Is the manuscript technically sound, and do the data support the conclusions?

Reviewer #1: Yes

Reviewer #2: Yes

**********

2. Has the statistical analysis been performed appropriately and rigorously?

Reviewer #1: N/A

Reviewer #2: Yes

**********

3. Have the authors made all data underlying the findings in their manuscript fully available?

The PLOS Data policy

Reviewer #1: Yes

Reviewer #2: Yes

**********

4. Is the manuscript presented in an intelligible fashion and written in standard English?

Reviewer #1: Yes

Reviewer #2: Yes

**********

Reviewer #1: Summary of the Manuscript

The study by Rahman et al., explores the Marbug virus genome to design two T- and B- cell multiple epitopes containing saRNA vaccine candidates, MB-saVaxI and MB- saVaxII, targeting infection with the virus. They use immunoinformatics and structural biology to achieve the study aims.

Strengths

Currently, there is limited research that specifically addresses a design of multi-epitope saRNA vaccines targeting Marbug virus – an infection of major global health concern. This study’s use of multi-epitope candidate antigen design, which is a concept that is gaining traction in immunology and vaccine development, as well as the use of self-amplifying RNA technology makes the research well innovative and needed. While the study objectives are clearly stated, the methodology appropriate and well-detailed, the results adequately supported by the data, and the conclusions justified; the following comments below if properly addressed would greatly improve the quality of the work.

Comments

1) In the Abstract, the statement that “Furthermore, MB-saVaxI and MB- saVaxII were effectively cloned into Escherichia coli,……” should be specified if this clonig was in vitro or in silico (predicted).

2) On page 9 last line, the authors should please define CFR on first use

3) Page 10, 3rd to 6th line, authors report 100% fatality rate, and later contradict with a 24% to 88% mortality rate range for the disease. Please resolve.

4) The background is well written, however, not much was discussed about the leading vaccine candidates currently in clinical trials, including the Sabin’s Marburg vaccine candidate phase II trial underway in Uganda and Kenya, and the United States. The authors might want to review the leading Marburg vaccine candidates and highlight gaps and limitations which the candidates (MB-saVaxI and MB-saVaxII) described herein are intended to overcome.

5) Method’s sections “2.3. Linear B cell (LBC) epitopes identification” and “2.8. Biophysical features evaluation” both describe prediction of Toxicity, allergenicity, and antigenicity. Please clarify if these were evaluated twice, otherwise, kindly keep the description in the relevant section

6) In Results section 3.4, the sentence “The percentage of epitopes….” Describes what percentage? Identity? Please specify.

7) Additionally, the supplementary data-1 tables 1 & 2 have wrongly reported percent identity ranges – e.g in supplementary table 1 for GP, I see percentages as low as 21.05% and as high as 100%, but this is different in the text where the range “94.74% to 89.847% ” reported within the text. Please the authors should kindly check across the entire manuscript to ensure data is correctly reported, matching between text and tables/figures; and appropriately cited.

8) Additionally, the percent identities should be reported in-text in a range that has the smaller percentage coming first, and the larger percentage coming second as the upper limit i.e please reverse the current ranges

9) A separate expanded paragraph detailing the study limitations should be added at the end of the discussion section. This is currently only very narrowly described. Authors should have to include statements explaining how limitations were overcome or not in the current study.

Recommendation

While the manuscript addresses an important topic, the above revisions are needed to improve clarity and ensure methodological and reporting rigor. I recommend minor revisions.

Reviewer #2: This is a good and relevant study addressing an important topic in Marburg virus vaccine development. The authors computationally designed two saRNA vaccine candidates, MB-saVaxI and MB-saVaxII, which showed favorable predicted structural, binding, stability, and immune-response profiles. However, several substantial issues require clarification and improvement. Therefore, I recommend Major Revision before the manuscript can be considered for publication.

**********

what does this mean?). If published, this will include your full peer review and any attached files.

If you choose “no”, your identity will remain anonymous but your review may still be made public.

**Do you want your identity to be public for this peer review?**  For information about this choice, including consent withdrawal, please see our Privacy Policy

Reviewer #1: No

Reviewer #2: No

**********

[NOTE: If reviewer comments were submitted as an attachment file, they will be attached to this email and accessible via the submission site. Please log into your account, locate the manuscript record, and check for the action link "View Attachments". If this link does not appear, there are no attachment files.]

To ensure your figures meet our technical requirements, please review our figure guidelines: https://journals.plos.org/plosone/s/figures

You may also use PLOS’s free figure tool, NAAS, to help you prepare publication quality figures: https://journals.plos.org/plosone/s/figures#loc-tools-for-figure-preparation.

NAAS will assess whether your figures meet our technical requirements by comparing each figure against our figure specifications.

Attachment Submitted filename: Immunoinformatics and ...pdf

# Author comment: Author response to Decision Letter 1

**DOI:** 10.1371/journal.pone.0361136.r002

29 Sep 2026

Response to Reviewers

Thank you to the reviewers for their insightful comments and suggestions, which helped improve the manuscript.

Reviewer #1

1. In the Abstract, the statement that “Furthermore, MB-saVaxI and MB- saVaxII were effectively cloned into Escherichia coli,……” should be specified if this cloning was in vitro or in silico (predicted).

Response: The nature of the cloning procedure has been clarified in the Abstract of the revised manuscript as “Furthermore, MB-saVaxI and MB-saVaxII were effectively cloned in silico into Escherichia coli,……….”

2. On page 9 last line, the authors should please define CFR on first use

Response: The full form of CFR was mentioned in the introduction as “The largest and most lethal outbreak occurred in Angola during 2004-2005, involving 252 cases and resulting in 227 deaths, corresponding to a case fatality rate (CFR) of ~90%, thereby underscoring the extreme lethality of the virus.”

3. Page 10, 3rd to 6th line, authors report 100% fatality rate, and later contradict with a 24% to 88% mortality rate range for the disease. Please resolve.

Response: The apparent discrepancy between the 100% fatality rate reported for the 2025 Tanzania outbreak and the general 24–88% CFR range has been clarified in the Introduction of the revised manuscript as “The most recent outbreak occurred in the United Republic of Tanzania in March 2025, with 10 cases and 10 deaths, corresponding to a 100% case fatality rate (16). The recurring nature of MARV outbreaks underscores the urgent need for improved zoonotic surveillance, rapid diagnostic tools, and coordinated global response mechanisms to mitigate the impact of the deadly disease (8). Due to its high mortality rate, ranging from 24% to 88% depending on outbreak conditions and case management, the World Health Organization (WHO) designated MARV as a priority pathogen in 2018 (10,17).”

4. The background is well written, however, not much was discussed about the leading vaccine candidates currently in clinical trials, including the Sabin’s Marburg vaccine candidate phase II trial underway in Uganda and Kenya, and the United States. The authors might want to review the leading Marburg vaccine candidates and highlight gaps and limitations which the candidates (MB-saVaxI and MB-saVaxII) described herein are intended to overcome.

Response: The leading Marburg vaccine candidates and their limitations have been discussed in the Discussion section of the revised manuscript as follows: “The most clinically advanced candidate, the Sabin Vaccine Institute's chimpanzee adenovirus type 3-vectored vaccine (cAd3-MARV) encoding the MARV glycoprotein, was safe and immunogenic in a first-in-human phase 1 trial in the United States (NCT03475056), with 95% of participants developing glycoprotein-specific antibodies (1). Nevertheless, as a replication-incompetent viral vector, cAd3-MARV may be difficult to administer repeatedly because pre-existing anti-vector immunity can reduce its effectiveness, particularly in adenovirus-exposed populations. In addition, its clinical efficacy has not yet been demonstrated. Similarly, live-attenuated rVSV∆G-MARV-GP candidates (PHV01, NCT06265012; IAVI's rVSV∆G-MARV-GP, NCT07425821) have entered phase 1 testing (2). However, these replication-competent live viral vaccines may cause reactogenicity and viremia and require strict cold-chain storage. In addition, human correlates of protection remain undefined, and their clinical efficacy has not yet been established (3,4).”

5. Method’s sections “2.3. Linear B cell (LBC) epitopes identification” and “2.8. Biophysical features evaluation” both describe prediction of Toxicity, allergenicity, and antigenicity. Please clarify if these were evaluated twice, otherwise, kindly keep the description in the relevant section

Response: The distinction between epitope-level and vaccine-level evaluation has been clarified in the revised manuscript. Toxicity, allergenicity, and antigenicity were assessed for the predicted linear B cell epitopes in Section 2.3, whereas the same properties were subsequently evaluated for the final vaccine constructs in Section 2.8. Thus, these analyses were performed at two distinct stages rather than as duplicate evaluations.

6. In Results section 3.4, the sentence “The percentage of epitopes….” Describes what percentage? Identity? Please specify.

Response: The type of percentage has been clarified in the Results section (Section 3.4) of the revised manuscript as “The conservancy percentage of epitopes ranges from 94.74% to 89.847% for GP, 100% to 50% for NP, 100% to 88.89% for VP40, and 100% to 75% for VP35 for MB-saVaxI.”

7. Additionally, the supplementary data-1 tables 1 & 2 have wrongly reported percent identity ranges – e.g in supplementary table 1 for GP, I see percentages as low as 21.05% and as high as 100%, but this is different in the text where the range “94.74% to 89.847% ” reported within the text. Please the authors should kindly check across the entire manuscript to ensure data is correctly reported, matching between text and tables/figures; and appropriately cited.

Response: The percentage identity ranges in Supplementary Data 1, Tables 1 and 2 have been carefully checked and corrected in the revised manuscript. The reported values have been cross-checked throughout the manuscript to ensure consistency between the text, tables, and figures, with appropriate citations provided.

8. Additionally, the percent identities should be reported in-text in a range that has the smaller percentage coming first, and the larger percentage coming second as the upper limit i.e please reverse the current ranges.

Response: All percentage identity ranges reported in the revised manuscript have been carefully checked and revised to present the smaller percentage first and the larger percentage as the upper limit.

9. A separate expanded paragraph detailing the study limitations should be added at the end of the discussion section. This is currently only very narrowly described. Authors should have to include statements explaining how limitations were overcome or not in the current study.

Response: The study limitations have been addressed in an expanded paragraph at the end of the Discussion section as, “In light of this, the proposed vaccine structure will probably maintain stability throughout the processes of entry into the host, transcription, and expression. Although the findings were favorable, this study focused exclusively on the immunoinformatics process. This process has limitations, including inaccurate structural predictions and a lack of chronic immunity predictions. Furthermore, all findings were generated through computational predictions, and no experimental validation was performed. Therefore, laboratory and animal studies are needed to confirm the immunogenicity and safety of MB-saVaxI and MB-saVaxII. Likewise, epitope prediction depends on the algorithms and databases used, which may not fully represent all HLA types in the global population. To address this bias, only epitopes with high conservancy were retained. Moreover, molecular dynamics simulations were performed over a limited timescale and may not capture long-term structural behavior. Overall, the stability and expression of the designed saRNA constructs were assessed only in-silico. These limitations were partly addressed through the use of validated tools and strict selection criteria. However, experimental studies are required before clinical translation. Further research, including preclinical and clinical studies, will be essential to precisely verify the immunogenicity, efficacy, and stability of these vaccines.”

Reviewer #2

1. This is a good and relevant study addressing an important topic in Marburg virus vaccine development. The authors computationally designed two saRNA vaccine candidates, MB-saVaxI and MB-saVaxII, which showed favorable predicted structural, binding, stability, and immune-response profiles. However, several substantial issues require clarification and improvement. Therefore, I recommend Major Revision before the manuscript can be considered for publication.

Response: The reviewer’s positive evaluation of the study and constructive comments are sincerely appreciated. All concerns raised have been carefully addressed, and the manuscript has been substantially revised, with the corresponding changes incorporated throughout the revised version.

Attachment Submitted filename: Response to Reviewers.docx

# Peer review: Decision Letter 1

**DOI:** 10.1371/journal.pone.0361136.r003

**Authors:** Mohammad Faezi Ghasemi

7 Oct 2026

Immunoinformatics and Reverse Vaccinology Design of Self-Amplifying RNA Vaccine Against Marburg Virus with Structural Dynamics and Immune Simulation Analysis

PONE-D-26-11532R1

Dear Dr. Mahdeen,

We’re pleased to inform you that your manuscript has been judged scientifically suitable for publication and will be formally accepted for publication once it meets all outstanding technical requirements.

Within one week, you’ll receive an e-mail detailing the required amendments. When these have been addressed, you’ll receive a formal acceptance letter and your manuscript will be scheduled for publication.

An invoice will be generated when your article is formally accepted. Please note, if your institution has a publishing partnership with PLOS and your article meets the relevant criteria, all or part of your publication costs will be covered. Please make sure your user information is up-to-date by logging into Editorial Manager at Editorial Manager® and clicking the ‘Update My Information' link at the top of the page. For questions related to billing, please contact billing support.

If your institution or institutions have a press office, please notify them about your upcoming paper to help maximize its impact. If they’ll be preparing press materials, please inform our press team as soon as possible -- no later than 48 hours after receiving the formal acceptance. Your manuscript will remain under strict press embargo until 2 pm Eastern Time on the date of publication. For more information, please contact onepress@plos.org.

Kind regards,

Mohammad Faezi Ghasemi, Ph.D

Academic Editor

PLOS One

Additional Editor Comments (optional):

Reviewers' comments:

Reviewer's Responses to Questions

**Comments to the Author**

Reviewer #1: All comments have been addressed

Reviewer #2: All comments have been addressed

**********

2. Is the manuscript technically sound, and do the data support the conclusions?

Reviewer #1: Yes

Reviewer #2: Yes

**********

3. Has the statistical analysis been performed appropriately and rigorously?

Reviewer #1: N/A

Reviewer #2: Yes

**********

4. Have the authors made all data underlying the findings in their manuscript fully available?

The PLOS Data policy

Reviewer #1: Yes

Reviewer #2: Yes

**********

5. Is the manuscript presented in an intelligible fashion and written in standard English?

Reviewer #1: No

Reviewer #2: Yes

**********

Reviewer #1: (No Response)

Reviewer #2: All of the points raised have been adequately and satisfactorily addressed. Following confirmation by the editorial team, I have no further concerns regarding the manuscript, and in my opinion, it is suitable for acceptance.

**********

what does this mean?). If published, this will include your full peer review and any attached files.

If you choose “no”, your identity will remain anonymous but your review may still be made public.

**Do you want your identity to be public for this peer review?**  For information about this choice, including consent withdrawal, please see our Privacy Policy

Reviewer #1: No

Reviewer #2: **Yes:**  Mehran Mahooti

**********

# Editor report: Acceptance letter

**DOI:** 10.1371/journal.pone.0361136.r004

**Authors:** Mohammad Faezi Ghasemi

PONE-D-26-11532R1

PLOS One

Dear Dr. Mahdeen,

I'm pleased to inform you that your manuscript has been deemed suitable for publication in PLOS One. Congratulations! Your manuscript is now being handed over to our production team.

At this stage, our production department will prepare your paper for publication. This includes ensuring the following:

* All references, tables, and figures are properly cited

* All relevant supporting information is included in the manuscript submission,

* There are no issues that prevent the paper from being properly typeset

You will receive further instructions from the production team, including instructions on how to review your proof when it is ready. Please keep in mind that we are working through a large volume of accepted articles, so please give us a few days to review your paper and let you know the next and final steps.

Lastly, if your institution or institutions have a press office, please let them know about your upcoming paper now to help maximize its impact. If they'll be preparing press materials, please inform our press team within the next 48 hours. Your manuscript will remain under strict press embargo until 2 pm Eastern Time on the date of publication. For more information, please contact onepress@plos.org.

You will receive an invoice from PLOS for your publication fee after your manuscript has reached the completed accept phase. If you receive an email requesting payment before acceptance or for any other service, this may be a phishing scheme. Learn how to identify phishing emails and protect your accounts at https://explore.plos.org/phishing.

If we can help with anything else, please email us at customercare@plos.org.

Thank you for submitting your work to PLOS One and supporting open access.

Kind regards,

PLOS One Editorial Office Staff

on behalf of

Dr. Mohammad Faezi Ghasemi

Academic Editor

PLOS One
